# *Pseudomonas aeruginosa* modulates alginate biosynthesis and type VI secretion system in two critically ill COVID-19 patients

**DOI:** 10.1186/s13578-022-00748-z

**Published:** 2022-02-09

**Authors:** Jiuxin Qu, Zhao Cai, Xiangke Duan, Han Zhang, Hang Cheng, Shuhong Han, Kaiwei Yu, Zhaofang Jiang, Yingdan Zhang, Yang Liu, Fang Bai, Yingxia Liu, Lei Liu, Liang Yang

**Affiliations:** 1 Department of Clinical Laboratory, Shenzhen Third People’s Hospital, Second Hospital Affiliated to Southern University of Science and Technology, Guangdong Provincial Clinical Research Center for Infectious Diseases (Tuberculosis), National Clinical Research Center for Infectious Diseases, Shenzhen, 518000 Guangdong China; 2 School of Medicine, Southern University of Science and Technology, Shenzhen, 518055 China; 3 Medical Research Center, Southern University of Science and Technology Hospital, Shenzhen, 518055 China; 4 School of Biological Sciences, Nankai University, Tianjin, 300071 China; 5 Shenzhen Key Laboratory of Pathogen and Immunity, State Key Discipline of Infectious Disease, Shenzhen Third People’s Hospital, Second Hospital Affiliated to Southern University of Science and Technology, Shenzhen, 518112 China; 6 Shenzhen Key Laboratory for Gene Regulation and Systems Biology, Southern University of Science and Technology, Shenzhen, 518055 China

**Keywords:** *Pseudomonas aeruginosa*, COVID-19, Bacterial superinfection, Type VI Secretion System, Biofilm

## Abstract

**Background:**

COVID-19 pneumonia has caused huge impact on the health of infected patients and associated with high morbidity and mortality. Shift in the lung microbial ecology upon such viral infection often worsens the disease and increases host susceptibility to superinfections. Bacterial superinfection contributes to the aggravation of COVID-19 and poses a great challenge to clinical treatments. An in-depth investigation on superinfecting bacteria in COVID-19 patients might facilitate understanding of lung microenvironment post virus infections and superinfection mechanism.

**Results:**

We analyzed the adaptation of two pairs of *P. aeruginosa* strains with the same MLST type isolated from two critical COVID-19 patients by combining sequencing analysis and phenotypic assays. Both *P. aeruginosa* strains were found to turn on alginate biosynthesis and attenuate type VI secretion system (T6SS) during short-term colonization in the COVID-19 patients, which results in excessive biofilm formation and virulence reduction-two distinct markers for chronic infections. The macrophage cytotoxicity test and intracellular reactive oxygen species measurement confirmed that the adapted *P. aeruginosa* strains reduced their virulence towards host cells and are better to escape from host immune clearance than their ancestors.

**Conclusion:**

Our study suggests that SARS-CoV-2 infection can create a lung environment that allow rapid adaptive evolution of bacterial pathogens with genetic traits suitable for chronic infections.

**Supplementary Information:**

The online version contains supplementary material available at 10.1186/s13578-022-00748-z.

## Background

Coronavirus Disease 2019 (COVID-19) induced by Severe Acute Respiratory Syndrome Coronavirus 2 (SARS-CoV-2) has caused over 4.7 million death globally as reported by WHO at Sep 2021. SARS-CoV-2 induces cytokine storm and hyperinflammation resulting in respiratory dysfunction and necrosis of epithelial cells of the lungs [1, 2]. Such histological changes in the lung tissues may result in dysbiosis of lung microbiome and give rise to secondary bacterial infection in COVID-19 patients [3]. Several groups of researchers have emphasized that microbial superinfection indeed occurs in COVID-19 patients, especially in severe cases, exacerbating disease progress and making difficulties to clinical treatments [4–7]. Nosocomial bacterial pathogens are risk factors contributing to virus superinfections which can lead to increased death of critically ill COVID-19 patients [8, 9]. The common superinfecting bacteria reported include *Mycoplasma pneumonia*, *Staphylococcus aureus*, *Klebsiella *spp., *Acinetobacter baumannii, Haemophilus influenzae*, *Pseudomonas aeruginosa* and etc. [10]. Bacteremia cases caused by *Pseudomonas* were also being reported [11].

*P. aeruginosa* is identified as a top superinfecting bacteria with SARS-CoV-2 in the Second Affiliated Hospital of Southern University of Science and Technology, China [12]. *P. aeruginosa* is a notorious nosocomial infection pathogen causing fatal infections in immunocompromised patients with diseases like cystic fibrosis, ventilator-associated pneumonia (VAP), catheter-associated infections and burn wounds [13, 14]. Patients with severe COVID-19 requiring ECMO had a very high rate of late-onset VAP, which is frequently caused by *P. aeruginosa*, with multiple recurrences and difficulties eradicating the pathogen from the lung [15]. This bacterium causes infections through different mechanisms, including secretion of a variety of virulence factors and biofilm formation [13]. *P. aeruginosa* engages diverse virulence mechanisms including quorum sensing systems, protein secretion systems, exopolysaccharides etc. for competition in the polymicrobial environments and escaping the host immune attack [13]. Longitudinal analysis of convergent evolution of *P. aeruginosa* clinical isolates from chronic lung infections showed that remodeling of biofilm formation and virulence factor is essential for host adaptation [16, 17]. Mutations in genes like *mucA, vgrG, lasR, rpoN* and *pvdS* significantly impair quorum sensing systems, protein secretion systems and biosynthesis of virulence factors, and allow *P. aeruginosa* to escape from host immune clearance in patients with lung diseases such as cystic fibrosis and ventilator-associated pneumonia [16–19]. Among the protein secretion systems, the type VI secretion system (T6SS) is particularly important for *P. aeruginosa* to compete with other microbial species and impair host cells [20]. Analysis of these evolutionary traits could contribute to the development of therapeutic measures to treat infections caused by *P. aeruginosa*.

Currently, characterization of bacterial adaptive evolution in COVID-19 patients is rare, which might limit our understanding of the lung microenvironment post SARS-CoV-2 infection. Although a recent study reported the change of *P. aeruginosa* in critically ill COVID-9 patients, it mainly focused on the evolution of its antimicrobial susceptibility over time [21]. To address this, we sequenced and compared the genomes and transcriptomes of four *P. aeruginosa* isolates sampled longitudinally from two critically ill COVID-19 patients, two isolates from each patient, to investigate its adaptation and pathogenesis patterns. The isolates collected from different patients are of the same sequence typing (ST type), indicating the possible occurrence of hospital-acquired *P. aeruginosa* infection. Interestingly, the later *P. aeruginosa* isolates form excessive biofilm by increasing alginate biosynthesis and developing mucoid phenotype, which are usually selected after long-term colonization in the lung environment. More notably, we demonstrated that *P. aeruginosa* attenuates its T6SS, especially HSI-II T6SS, to suppress its virulence and escape host clearance during superinfection. Our study suggests that SARS-CoV-2 infection can create a lung environment that allow rapid adaptive evolution of bacterial pathogens with genetic traits suitable for chronic infections.

## Results

### Post viral *P. aeruginosa* infection in the respiratory track of COVID-19 patients

During routine screening of respiratory samples (sputum samples and bronchioalveolar lavage fluids) of COVID-19 patients, we discovered that two patients were colonized by *P. aeruginosa*. These two patients were living in the same ward in the department of Infectious Diseases. Clinical data of the two patients indicated that they were diagnosed to have bacterial pneumonia in addition to COVID-19 pneumonia. In total, four *P. aeruginosa* isolates were collected from respiratory samples, sputum samples or bronchioalveolar lavage fluids (BALF) samples of the two patients and named as LYSZa2, LYSZa3, LYSZa5 and LYSZa6 respectively. Among which, LYSZa2 (sputum) and LYSZa3 (BALF) were isolated from patient 1 on day 12 (the day of ventilation) and day 15 of hospitalization, while LYSZa5 (BALF) and LYSZa6 (sputum) were isolated from patient 2 on day 17 (10 days after ventilation) and day 32 of hospitalization. We defined LYSZa2 and LYSZa5 as the ancestry isolates while LYSZa3 and LYSZa6 as the progeny isolates. To investigate epidemiological link between these *P. aeruginosa* isolates, genomes of all isolates were sequences by using Illumina HiSeq platform. Multi-Locus Sequence Typing (MLST) analysis indicated that four isolates collected from these two patients are of the same type, *P. aeruginosa* ST1074, suggesting that these *P. aeruginosa* isolates could be hospital-acquired. To find out if *P. aeruginosa* is the dominant superinfecting species in these COVID-19 patients causing post-viral bacterial infection, we assessed the microbiome of the sputum samples using metagenomics sequencing analysis. Results of taxonomic classification and species abundance analysis indicated that *P. aeruginosa* is the most abundant species in the microbiome of all four sputum samples (Additional file 1: Fig. S1A and B). Alpha diversity analysis indicated that there is no significant difference in the composition of microbiome between the progeny isolates and the ancestor isolates (Additional file 1: Fig. S1C). Since *P. aeruginosa* is well known to engage adaptive evolution during colonization in the respiratory track [16, 17], we then focused on these isolates to analyze their adaptive evolutionary traits during colonization in COVID-19 environment. Genomes of the first isolate of each patient, LYSZa2 and LYSZa5, were further sequenced using Pacific Biosciences RSII sequencer for further analysis.

### Characterization of genomes, phylogeny and antibiotic resistance of *P. aeruginosa* isolates

Circular genomes of LYSZa2 and LYSZa5 were assembled while the genome sizes are 6, 638, 980 bp and 6, 638, 990 bp, with 66.2% of GC respectively. Rearrangements were checked and manually curated after pairwise comparison of the two genomes. As predicted by PGAP, LYSZa2 genome contains 6183 genes among which 6046 features encode for proteins and 86 encode for RNAs, while LYSZa5 genome contains 6183 genes among which 6045 features encode for proteins and 86 encodes for RNAs. These two genomes are almost identical, which again suggests a hospital acquired infection and indicates that they are of same clone.

Both of the *P. aeruginosa* genomes contain the same antimicrobial resistance genes predicted by ResFinder, including *crpP*, *aph(3')-IIb*, *catB7*, *blaOXA-50*, *blaPAO*, and *fosA* against fluoroquinolone, aminoglycoside, phenicol, beta-lactam and fosfomycin drugs. No difference in antimicrobial genes was identified between the ancestry and the progeny isolates. According to the clinical information, patient 1 (LYSZa2&3) received a series of antimicrobials until the progeny *P. aeruginosa* was isolated, including moxifloxacin or piperacillin–tazobactam, or meropenem; while patient 2 (LYSZa5&6) received a series of antimicrobials until the progeny *P. aeruginosa* was isolated, including moxifloxacin or cefoperazone–sulbactam, or meropenem combined with vancomycin. In vitro antimicrobial resistance of the isolates was tested using antibiotics including ceftazidime, piperacillin, cefoperazone/sulbactam, imipenem, aztreonam and levofloxacin according to the antibiotic susceptibility guidelines provided by CLSI. Obvious differences in resistance were observed between the ancestry and the progeny isolates. LYSZa5 is sensitive to ceftazidime, piperacillin, cefoperazone/sulbactam, imipenem and aztreonam while LYSZa6 becomes intermediate or resistance to these antibiotics after 15 days of evolution probably due to the upregulation of *mexAB-oprM* genes encoding for efflux pump (Additional file 3: Table S1). This increase in the expression of these efflux pump genes was probably due to the downregulation of their transcriptional regulator, *nalC* gene (Additional file 9: Table S7). Antibiotic treatment is probably a driver for such evolution. However, no obvious change in resistance to these drugs was observed between LYSZa2 and LYSZa3 probably due to the short evolving time (Additional file 3: Table S1).

**Fig. 1:**
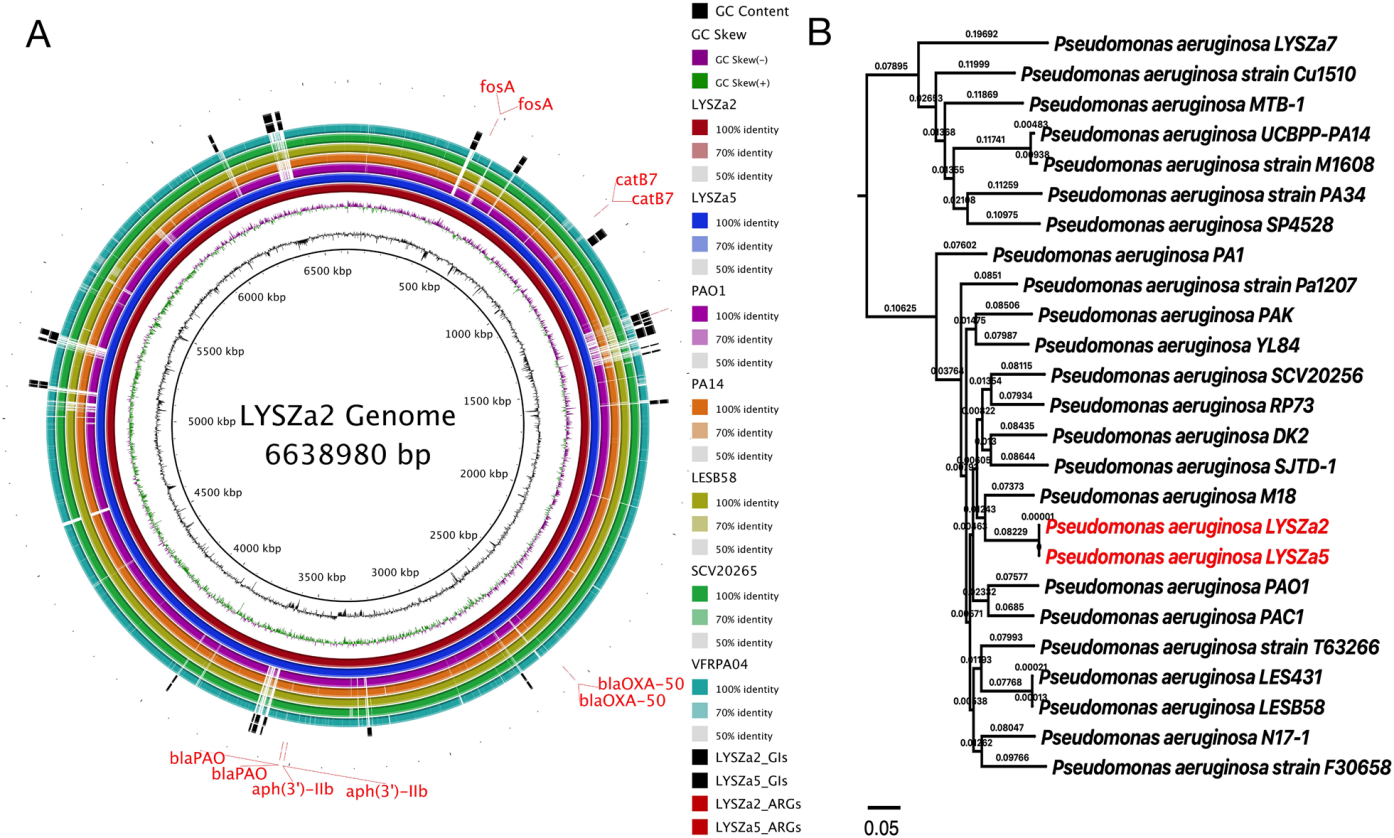
Ring Plot and Phylogenetic tree of LYSZa2 and LYSZa5. **A** Ring plot of LYSZa2 and LYSZa5 comparing with other *P. aeruginosa* genomes. From the innermost, ring 1: GC content (black); ring 2: GC skew (purple/green); ring 3: LYSZa2(dark red); ring 4: LYSZa5(navy); ring 5: PAO1 lab reference strain (violet); ring 6: PA14 clinical virulent strain (orange); ring 7: LESB58 hypervirulent clinical strain from CF patient (olive); ring 8: SCV20265 small colony variant strain (light green); ring 9: VRFPA04 multidrug resistant strain from keratitis patient(cyan); ring 10 and 11: GIs on LYSZa2 and LYSZa5 (black); ring 12 and 13: ARGs on LYSZa2 and LYSZa5 (red); **B** Phylogenetic tree constructed using LYSZa2 and LYSZa5 genomes and 23 other *P. aeruginosa* genomes based on core genome alignment, LYSZa2 and LYSZa5 are highlighted in red

Genomic islands (GIs) on LYSZa2 and LYSZa5 genomes were predicted by IslandViewer4 (Additional file 4: Table S2 Additional file 5: TableS3). In total, 35 GIs on LYSZa2 and 36 GIs on LYSZa5 were predicted by at least one prediction method. Genomes of LYSZa2 and LYSZa5 were compared with genomes of five other *P. aeruginosa* strains including PAO1 reference strain and four virulence strains, PA14, LESB58, SCV20265 and VFRPA04 (Fig. 1A). Most of the GIs predicted are specific to LYSZa2 and LYSZa5 genomes (Fig. 1A). Genes in these GIs are involved in transcriptional regulation, DNA restriction-modification, DNA repair, toxin-antitoxin and secretion systems, showing that these GIs maybe important for *P. aeruginosa* survival and virulence during superinfection with SARS-CoV-2.

We then traced the origin of these isolates by constructing phylogenetic tree using core genomes of LYSZa2 and LYSZa5 with 22 other clinical or environmental *P. aeruginosa* genomes selected from NCBI/Pseudomonas genome database (Additional file 6: Table S4) and one SARS-CoV-2 superinfecting strain published by our group recently, *P. aeruginosa* LYSZa7[22]. As seen from the phylogenetic tree (Fig. 1B), LYSZa2 and LYSZa5 are closed related without evolutionary distance between them, and are phylogenetically approximate to the PAO1 reference strain and the hypervirulent isolate LESB58 from a CF patient, rather than the hypervirulent PA14 strain.

Single Nucleotide Polymorphism (SNP) and other genome modifying events were assessed between the ancestry isolates and the progeny isolates respectively using PAO1 as reference (Additional file 7: Table S5). 90 genomic modifying events including nucleotide variation, insertion, deletion and replacement (Additional file 7: Table S5) were identified in LYSZa3 as compared to LYSZa2. 93 of such genomic modifying events (Additional file 7: Table S5) were identified in LYSZa6 as compared to LYSZa5. Common mutations were found between LYSZa3 and LYSZa6 on genes related to type VI secretion system and iron transport when compared with their ancestors, respectively (Table 1). Such observation indicated that *P. aeruginosa* undergoes adaptive evolution in COVID-19 patients to survive and modulate its virulence during the post virus infection. We then performed RNA sequencing to evaluate the changes at transcriptional level during *P. aeruginosa* evolution.

**Table 1:** Common genetic mutations identified between LYSZa3 and LYSZa6

		LYSZa3 vs LYSZa2				LYSZa6 vs LYSZa5			
Gene	Product	Type	Amino acid change	Coding region change in longest transcript	Non-synonymous	Type	Amino acid change	Coding region change in longest transcript	Non-synonymous
PA0041	Hemagglutinin	SNV		5803C > T	No	SNV		5803C > T	No
PA0259	Type 6 lipase adaptor, Tla3	SNV		333A > G	No	SNV		333A > G	No
		MNV		330_331delinsTT	No	MNV		330_331delinsTT	No
		SNV		327 T > G	No	SNV		327 T > G	No
		SNV		324 T > A	No	SNV		324 T > A	No
		MNV	Ser107Asn	320_321delinsAT	Yes	MNV	Ser107Asn	320_321delinsAT	Yes
		SNV		315 T > C	No	SNV		315 T > C	No
		SNV	Asp105Asn	313G > A	Yes	SNV	Asp105Asn	313G > A	Yes
		SNV		303G > A	No	SNV		303G > A	No
*fiuA*	Ferrichrome receptor FiuA	SNV		1854C > T	No	SNV		1854C > T	No
		SNV		1842G > C	No	SNV		1842G > C	No
		SNV		1830G > C	No	SNV		1830G > C	No
		SNV	Ile608Met	1824C > G	Yes	SNV	Ile608Met	1824C > G	Yes
		SNV	Ile608Leu	1822A > C	Yes	SNV	Ile608Leu	1822A > C	Yes
		SNV		1812G > A	No	SNV		1812G > A	No
		SNV		1800G > C	No	SNV		1800G > C	No
		SNV		1797G > A	No	SNV		1797G > A	No
		SNV		1794G > C	No	SNV		1794G > C	No
		SNV		1792C > T	No	SNV		1792C > T	No
		Deletion	Ser597fs	1790delC	Yes	Deletion	Ser597fs	1790delC	Yes
		SNV	Ser596Arg	1788 T > A	Yes	SNV	Ser596Arg	1788 T > A	Yes
		Insertion	Ser596fs	1786dupA	Yes	Insertion	Ser596fs	1786dupA	Yes
		SNV	Thr595Asn	1784C > A	Yes	SNV	Thr595Asn	1784C > A	Yes
		MNV		1780_1781delinsTC	No	MNV		1780_1781delinsTC	No
		Insertion	Asn593fs	1778_1779insGG	Yes	Insertion	Asn593fs	1778_1779insGG	Yes
		MNV	Met592_Asn593delinsIleTyr	1776_1777delinsAT	Yes	MNV	Met592_Asn593delinsIleTyr	1776_1777delinsAT	Yes
		Replacement	Met592fs	1773delinsCG	Yes	Replacement	Met592fs	1773delinsCG	Yes
PA4514	Iron transport outer membrane receptor	SNV	Thr193Ile	578C > T	Yes	SNV	Thr193Ile	578C > T	Yes
		Replacement	Asp190fs	569_570delinsG	Yes	Replacement	Asp190fs	569_570delinsG	Yes
		SNV	Gln189Leu	566A > T	Yes	SNV	Gln189Leu	566A > T	Yes
		Insertion	Gln189fs	564dupG	Yes	Insertion	Gln189fs	564dupG	Yes
PA5088	Type VI secretion lipase immunity protein, Tli5b3	SNV	Gly181Ser	541G > A	Yes	SNV	Gly181Ser	541G > A	Yes
		SNV		528G > C	No	SNV		528G > C	No

Full list of genetic mutations is included in Additional file 7: Table S5

### Increase in alginate biosynthesis of *P. aeruginosa* during adaptation in COVID-19 patients

**Fig. 2:**
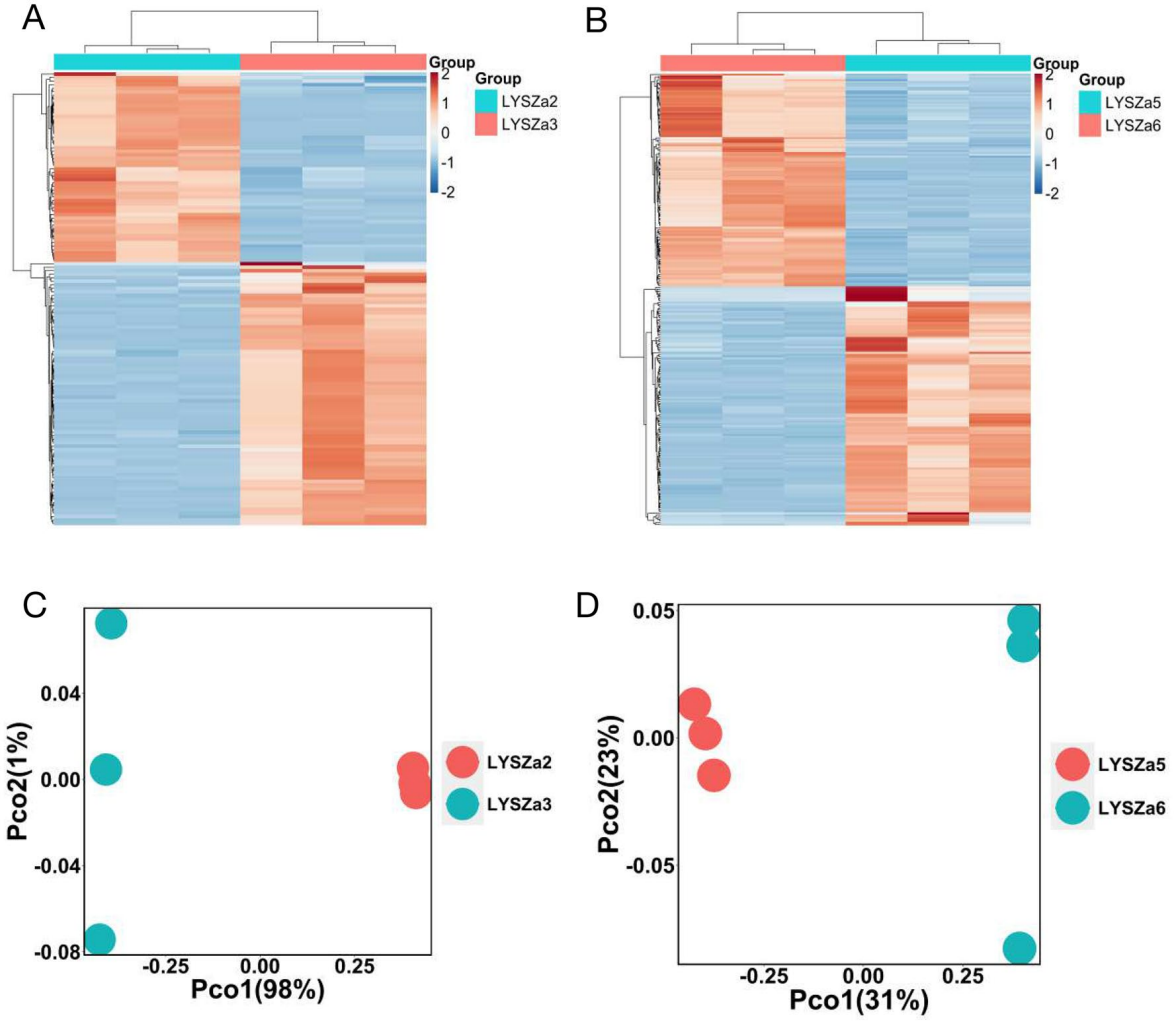
Heatmaps and PCoA plots of DEGs between the ancestry isolates and the progeny isolates. **A** Heatmap of significant DEGs in LYSZa3 compared to LYSZa2; **B** heatmap of significant DEGs in LYSZa6 compared to LYSZa5; **C** PCoA plots of LYSZa3 group and LYSZa2 group based on DEGs using Bray Curtis dissimilarity; **D** PCoA plots of LYSZa6 group and LYSZa5 group based on DEGs using Bray Curtis dissimilarity

To further elucidate the *P. aeruginosa* adaptation during colonization in the COVID-19 patients, we performed RNA sequencing analysis of these isolates using Illumina HiSeq platform and focused on discovering the differential expressed genes between the ancestry and progeny isolates longitudinally. Moreover, we also compared the differential gene expression between the progeny cells of the two patients to learn the parallel changes of *P. aeruginosa* in different hosts. Based on the filtering criteria of fold change ≥ 4, adjusted p-value < 0.05 and base mean ≥ 20, the expression of 129 genes were differentially regulated in LYSZa3 as compared to LYSZa2, among which 75 were upregulated and others were downregulated (Additional file 8: Table S6). In LYSZa6 as compared to LYSZa5, 242 genes were differentially expressed, among which 114 were upregulated and others were downregulated (Additional file 9: Table S7). Heatmaps illustrating these differentially expressed genes (DEGs) display distinctive transcriptomic profiles between the progeny isolates and the ancestry isolates (Fig. 2A and B). We further clustered the isolates using Principal Coordinates Analysis (PCoA) based on Bray Curtis dissimilarity to check the differences between different groups. As seen from PCoA plots (Fig. 2C and D), LYSZa2 group and LYSZa5 group separated clearly from LYSZa3 group and LYSZa6 group respectively, indicating that the ancestry isolates and the progeny isolates possess distinct physiology. 74 common genes in total were found to be differentially regulated in both progeny isolates, LYSZa3 and LYSZa6 (Additional file 10: Table S8), indicating parallel changes evolved from same *P. aeruginosa* clone in different hosts during superinfection with SARS-CoV-2 virus.

**Fig. 3:**
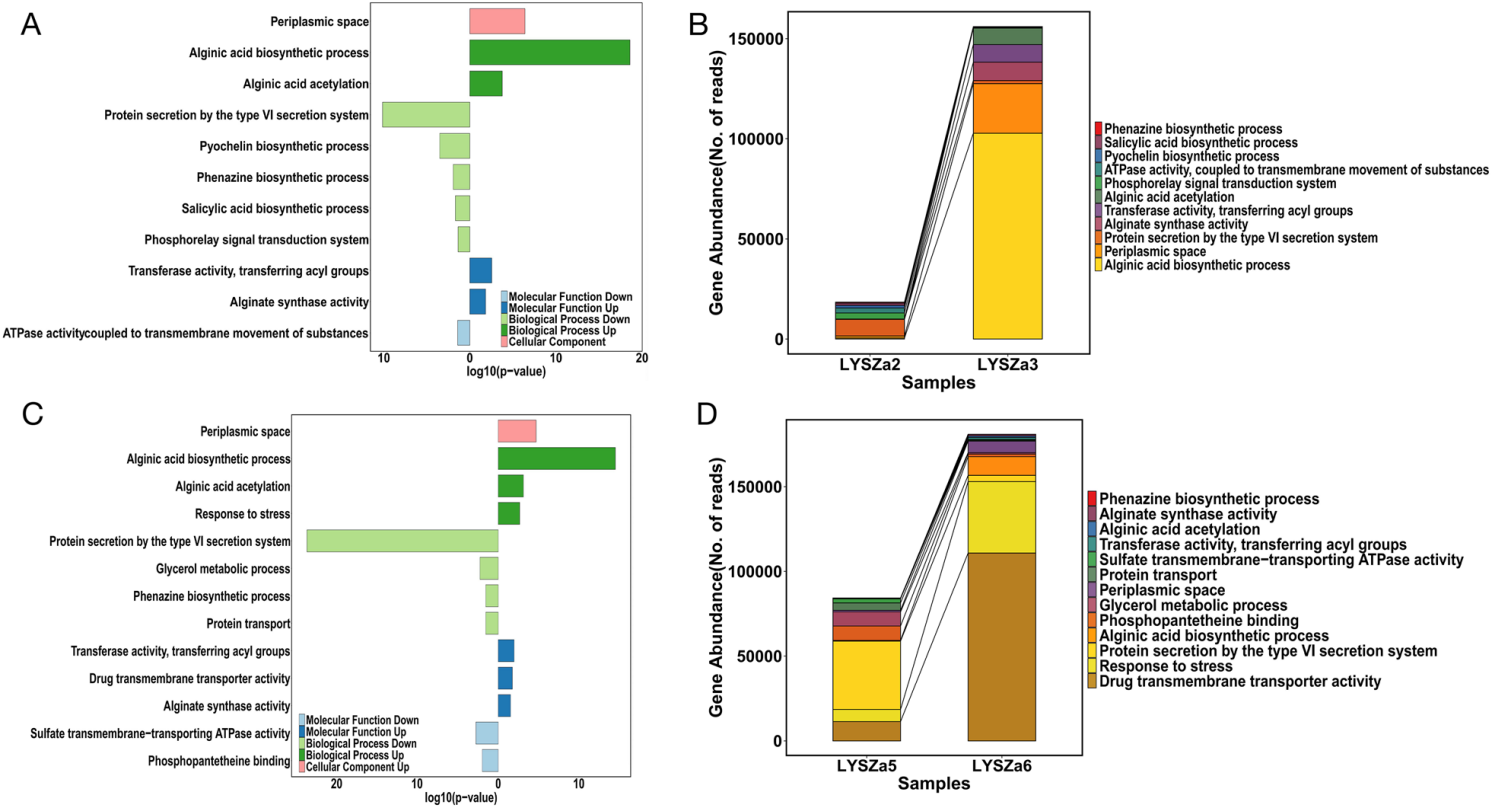
GO enrichment analysis and variation in gene abundance in each GO. **A** GO enriched in LYSZa3 compared to LYSZa2, bars towards right-hand side showing genes assigned to the functions were upregulated, bars towards left-hand side showing genes assigned to the functions were downregulated, up- and down-regulation are indicated in the legend as well, p-value < 0.05; **B** Variation in gene abundance involved in the enriched GO functions listed in A in LYSZa2 and LYSZa3; **C** GO enriched in LYSZa6 compared to LYSZa5, bars towards right-hand side showing genes assigned to the functions were upregulated, bars towards left-hand side showing genes assigned to the functions were downregulated, up- and down-regulation are indicated in the legend as well, p-value < 0.05; **D** Variation in gene abundance involved in the enriched GO functions listed in C in LYSZa5 and LYSZa6

**Fig. 4:**
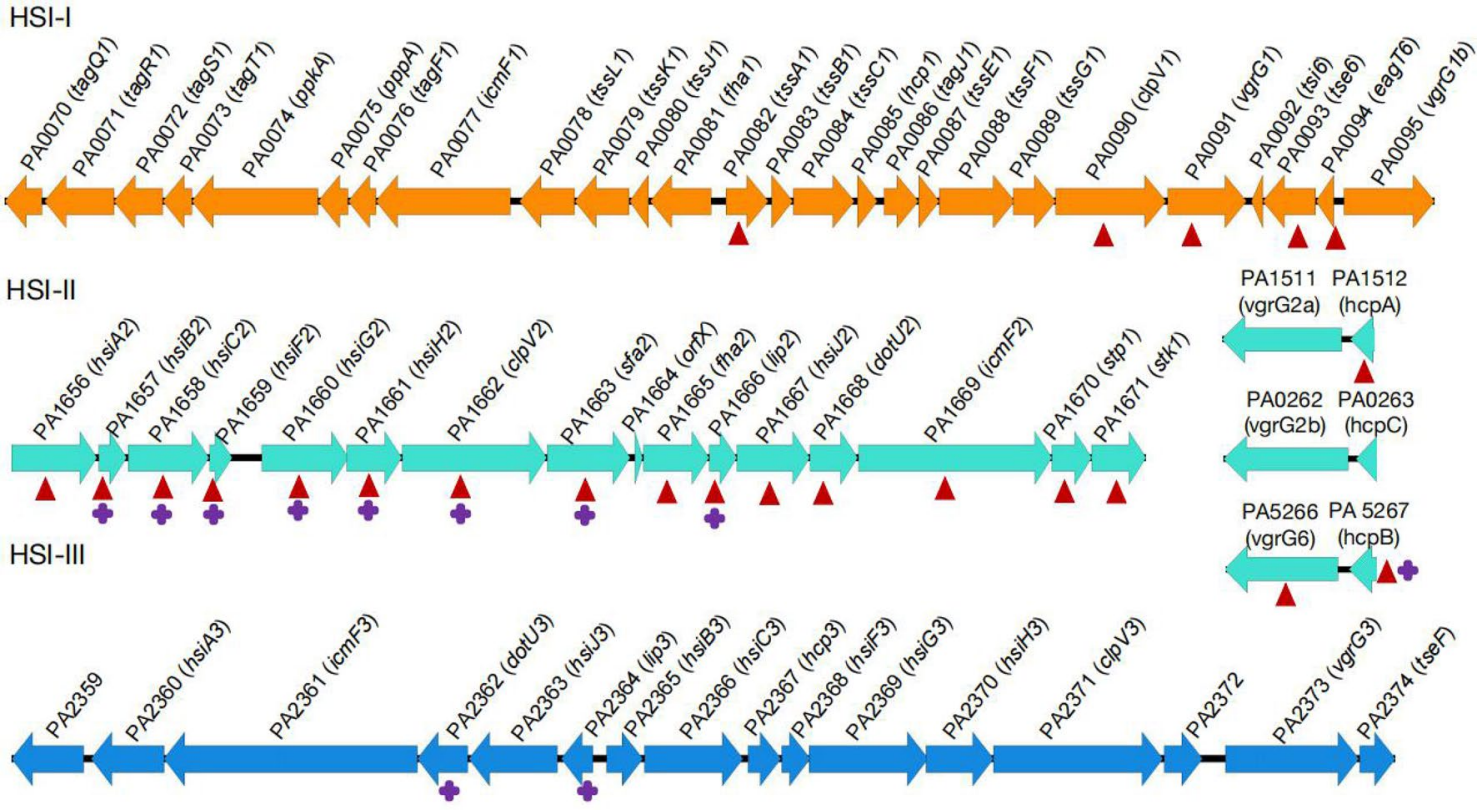
Schematic figure of HSI-I, HSI-II and HSI-III gene clusters. Genes downregulated in LYSZa3 are denoted by purple crosses; genes downregulated in LYSZa6 are denoted by red triangles. Gene name was obtained from Pseudomonas Genome Database [49]

We next performed Gene Ontology (GO) enrichment analysis using the DEGs to identify highly enriched functions in the progeny isolates (Fig. 3). 11 GO classes consisting of 7 biological processes, 3 molecular functions and 1 cellular component were enriched in LYSZa3 as compared to LYSZa2 (Fig. 3A). Whereas in LYSZa6 compared to LYSZa5, 13 GO classes consisting of 7 biological processes, 5 molecular functions and 1 cellular component were highly enriched (Fig. 3C). Among all of these enriched functions in both the progeny isolates, most significantly enriched biological processes are alginic acid biosynthetic process and protein secretion by the type VI secretion system (T6SS). All of 12 genes assigned to alginic acid biosynthetic process (*algD*, *algX*, *algA*, *algE*, *algF*, *algL*, *alg44*, *algJ*, *algK*, *alg8*, *algI*, *algG*) were upregulated in LYSZa3 for 16.89 to 1091.52 folds (Table 2). The expression of same genes also increased significantly in LYSZa6 for 4.45 to 138.79 folds (Table 3). Eight genes assigned to protein secretion by T6SS, PA1657 (*hsiB2*), PA1658 (*hsiC2*), PA1659 (*hsiF2*), PA1660 (*hsiG2*), PA1661 (*hsiH2*), PA1662 (*clpV2*), PA1663 (*sfa2*), PA1666 (*lip2*), were all significantly downregulated in LYSZa3 for 4.46 to 6.47 folds (Table 2). In LYSZa6, beside the same eight genes mentioned above, seven other genes assigned to protein secretion by T6SS were also downregulated for 4.46 to 17.11 folds, including PA1656 (*hsiA2*), PA1665 (*fha2*), PA1667 (*hsiJ2*), PA1668 (*dotU2*), PA1669 (*icmF2*), *stk1* and *stp1* (Table 3). Besides these genes assigned to T6SS by GO enrichment analysis, the expression of several other genes involved in T6SS, *hcpB*, *lip3* (PA2364), and *dotU3* (PA2362), were also decreased in LYSZa3 (Table 2). While the expression of 15 other T6SS genes significantly decreased in LYSZa6, including *clpV1*, PA3904 (PAAR4), PA2702 (*tse2*), PA2774 (*tse4*), PA2775 (*tsi4*), *vgrG1*, PA0093 (*tse6*), PA3905 (*tecT*), PA0082 (*tssA1*), PA2703 (*tsi2*), PA0094 (*eagT6*), PA3484 (*tse3*), PA5266 (*vgrG6*), *hcpA* and *hcpB* (Table 3). *P. aeruginosa* carries three sets of T6SS systems, HSI-I, HSI-II and HSI-III respectively, to regulate its virulence to bacterial neighbors and host cells. T6SS is a phage-like structure injecting exotoxins to neighboring cells and consists of several key structural components including sheath and baseplate components encoded by *tss*/*hsi* genes, spike proteins encoded by *vgrG* genes, syringe proteins encoded by *hcp* genes, ATPases encoded by *clpV* genes and etc. As illustrated in Fig. 4, the downregulated genes in both progeny isolates, especially those of LYSZa6, are mostly involved in HSI-II gene cluster with several others scattered on another two gene clusters. The higher number of DEGs observed in LYSZa6 as compared to LYSZa3 is likely due to the longer evolving time of LYSZa6 within the host. The changes in the gene abundance assigned to different GO functions are illustrated in Fig. 3B and D. There was a dramatic increase in the abundance of genes involved in alginic acid biosynthetic process and significant decrease in the abundance of genes involved in protein secretion by T6SS in the progeny isolates. Such observation conveyed excessive biosynthesis of alginate and suppression of T6SS in the progeny isolates during colonization. Besides T6SS, T3SS is another important determinant for *P. aeruginosa* virulence to infect host cells. The results of RNA-Seq indicated that most of the T3SS genes expressed at low levels in all four isolates without significant differences between the progeny isolates and the ancestor isolates (data not shown). To confirm such observation, we selected few genes encoding for different structural components of T6SS (*vgrG1* for needle tip, *hcpA* for secreted protein, *hsiC2* for sheath component and *clpV2* for ATPase) and T3SS (*popD* for translocation apparatus, *pscF* for needle filament and *pscN* for ATPase complex) for RT-PCR examination. The results of RT-PCR tests (Additional file 2: Fig. S2) showed that the expression level of T6SS genes indeed decreased in the progeny isolates while the expression of T3SS genes remained low and insignificantly differentiated between the progeny and the ancestor. Though the decrease in expression of *pscF* gene in LYSZa6 needed to be further studied, such results suggested that T3SS plays minimal role in regulating the virulence of the isolates towards host cells.

**Table 2:** Selected DEGs in LYSZa3 compared to LYSZa2 related to alginate biosynthesis and type VI secretion system

Gene	Product name	Base mean	Fold change	Adj.p-value	LYSZa2 Mean	LYSZa3 Mean
*algD*	GDP-mannose 6-dehydrogenase AlgD	27,877.94	1091.52	0.00E+00	61.33	50,065.00
*algX*	Alginate biosynthesis protein AlgX	1967.25	256.42	2.31E−236	18.33	3478.67
*algA*	Phosphomannose isomerase/guanosine 5'-diphospho-D-mannose pyrophosphorylase	5853.40	238.02	0.00E+00	59.00	10,382.00
*algE*	Alginate production outer membrane protein AlgE precursor	2204.36	221.80	3.29E−252	24.00	3889.33
*algF*	Alginate *o*-acetyltransferase AlgF	1648.75	211.59	3.00E−242	18.67	2924.00
*algL*	Poly(beta-d-mannuronate) lyase precursor AlgL	2134.58	182.28	4.64E−245	28.00	3766.33
*alg44*	Alginate biosynthesis protein Alg44	1855.41	162.63	1.64E−285	27.33	3260.33
*algJ*	Alginate *o*-acetyltransferase AlgJ	1033.36	154.76	2.12E−191	16.00	1783.33
*algK*	alginate biosynthetic protein AlgK precursor	1171.78	145.20	2.18E−233	19.33	2043.00
*alg8*	Alginate biosynthesis protein Alg8	2758.04	133.33	0.00E+00	49.67	4939.33
*algI*	Alginate *o*-acetyltransferase AlgI	1457.21	59.29	1.83E−259	58.33	2501.67
*algG*	Alginate-c5-mannuronan-epimerase AlgG	1682.20	16.89	1.73E−125	226.00	2793.33
*hcpB*	Secreted protein Hcp	107.95	− 4.46	1.17E−20	215.67	36.00
PA1663	Sfa2	305.43	− 4.46	2.74E−47	603.33	100.00
PA1666	Lip2	159.33	− 4.50	2.97E−24	319.00	52.67
PA2362	DotU3	22.49	− 4.54	4.95E−06	44.00	7.00
PA1660	HsiG2	346.81	− 4.63	1.56E−59	686.67	110.33
PA1662	clpV2	1014.01	− 4.63	3.41E−44	2020.67	318.00
PA1661	HsiH2	246.25	− 4.65	1.19E−39	488.33	77.67
PA1659	HsiF2	163.52	− 5.59	1.38E−34	334.00	43.00
PA1657	HsiB2	842.21	− 6.21	6.69E−109	1743.67	204.33
PA1658	HsiC2	1839.66	− 6.47	4.92E−141	3824.67	437.00
PA2364	Lip3	32.22	− 9.20	6.01E−13	70.33	5.67

Filtering criteria are fold change ≧ 4, adjusted p-value < 0.05 and base mean ≧ 20. Full list of DEGs is included in Additional file 8: Table S6

**Table 3:** Selected DEGs in LYSZa6 compared to LYSZa5 related to alginate biosynthesis and type VI secretion system

Gene	Product name	Base mean	Fold change	Adj p-value	LYSZa2 Mean	LYSZa3 Mean
*algD*	GDP-mannose 6-dehydrogenase AlgD	2956.90	138.79	2.47E–170	39.33	6375.00
*algX*	Alginate biosynthesis protein AlgX	271.80	45.97	1.05E–75	10.67	585.67
*algL*	Poly(beta-d-mannuronate) lyase precursor AlgL	283.61	44.85	1.98E–74	11.33	609.00
*algJ*	Alginate o-acetyltransferase AlgJ	118.03	32.97	3.93E–33	6.33	248.67
*algE*	Alginate production outer membrane protein AlgE precursor	254.48	25.19	1.11E–61	18.33	535.33
*algA*	Phosphomannose isomerase/guanosine 5'-diphospho-d-mannose pyrophosphorylase	481.88	21.52	9.22E–64	39.00	1000.67
*algF*	Alginate *o*-acetyltransferase AlgF	152.00	18.68	7.50E–42	14.33	314.67
*alg8*	Alginate biosynthesis protein Alg8	292.51	14.91	1.09E–59	34.67	599.33
*alg44*	Alginate biosynthesis protein Alg44	195.56	14.43	3.38E–61	23.67	403.00
*algK*	Alginate biosynthetic protein AlgK precursor	135.65	10.35	6.37E–39	21.67	272.67
*algI*	Alginate *o*-acetyltransferase AlgI	218.14	8.40	7.47E–47	43.00	427.33
*algG*	Alginate-c5-mannuronan-epimerase AlgG	356.62	4.45	4.15E–53	121.33	644.33
*clpV1*	ClpV1	5315.67	− 4.08	7.48E–33	7854.33	2309.33
PA3904	PAAR4	1457.06	− 4.17	3.79E–33	2178.00	631.67
PA2702	Tse2	280.90	− 4.35	2.50E–29	419.33	117.33
PA2774	Tse4	296.61	− 4.37	4.85E–26	461.67	122.33
PA2775	Tsi4	133.81	− 4.71	7.24E–19	212.67	52.33
*vgrG1*	VgrG1	2637.35	− 4.74	1.56E–50	3969.33	1016.67
PA0093	Tse6	947.79	− 4.91	3.88E-–46	1469.00	360.00
PA3905	Type VI effector chaperone for Tox-Rease, TecT	1073.56	− 4.95	1.10E–53	1671.00	403.00
PA0082	TssA1	1449.46	− 5.32	1.76E–55	2265.00	515.00
PA2703	Tsi2	98.26	− 5.56	5.99E–24	156.67	33.00
PA0094	EagT6	251.06	− 5.79	1.43E–38	401.67	82.00
PA3484	Tse3	544.56	− 5.86	1.50E–54	869.33	174.33
*stk1*	Stk1	263.85	− 5.90	1.38E–56	423.00	84.67
PA5266	VgrG6	396.48	− 6.81	6.50E–47	654.33	113.33
PA1667	HsiJ2	678.54	− 6.99	2.41E–100	1119.67	188.67
PA1656	HsiA2	3257.03	− 7.01	4.84E–80	5349.67	911.33
PA1662	clpV2	2538.08	− 8.98	3.88E–108	4332.33	564.33
PA1661	HsiH2	693.51	− 9.91	1.55E–110	1186.67	142.00
PA1666	Lip2	340.97	− 10.48	2.48E–72	589.00	65.67
*stp1*	Stp1	327.95	− 10.61	1.67E–70	562.67	62.67
*hcpA*	Secreted protein Hcp	72.34	− 10.75	7.25E–26	125.33	13.67
PA1665	Fha2	840.73	− 10.96	1.12E–108	1456.67	155.33
PA1660	HsiG2	907.03	− 11.05	1.78E–125	1551.67	167.33
PA1669	IcmF2	2052.74	− 11.08	6.20E–107	3547.33	375.67
*hcpB*	Secreted protein Hcp	204.27	− 11.30	8.81E–47	358.00	36.67
PA1668	DotU2	487.58	− 11.30	1.39E–105	836.33	88.00
PA1663	Sfa2	883.17	− 12.92	4.47E–74	1556.67	139.33
PA3334	Acp3	236.04	− 13.79	1.82E–73	415.67	35.33
PA1657	HsiB2	2539.11	− 14.39	1.32E–112	4445.00	366.00
PA1659	HsiF2	480.65	− 17.06	4.25E–89	843.33	59.00
PA1658	HsiC2	5746.91	− 17.11	4.82E–66	10,179.00	695.67

Filtering criteria are fold change ≧ 4, adjusted p-value < 0.05 and base mean ≧ 20. Full list of DEGs is included in Additional file 9: Table S7

**Fig. 5:**
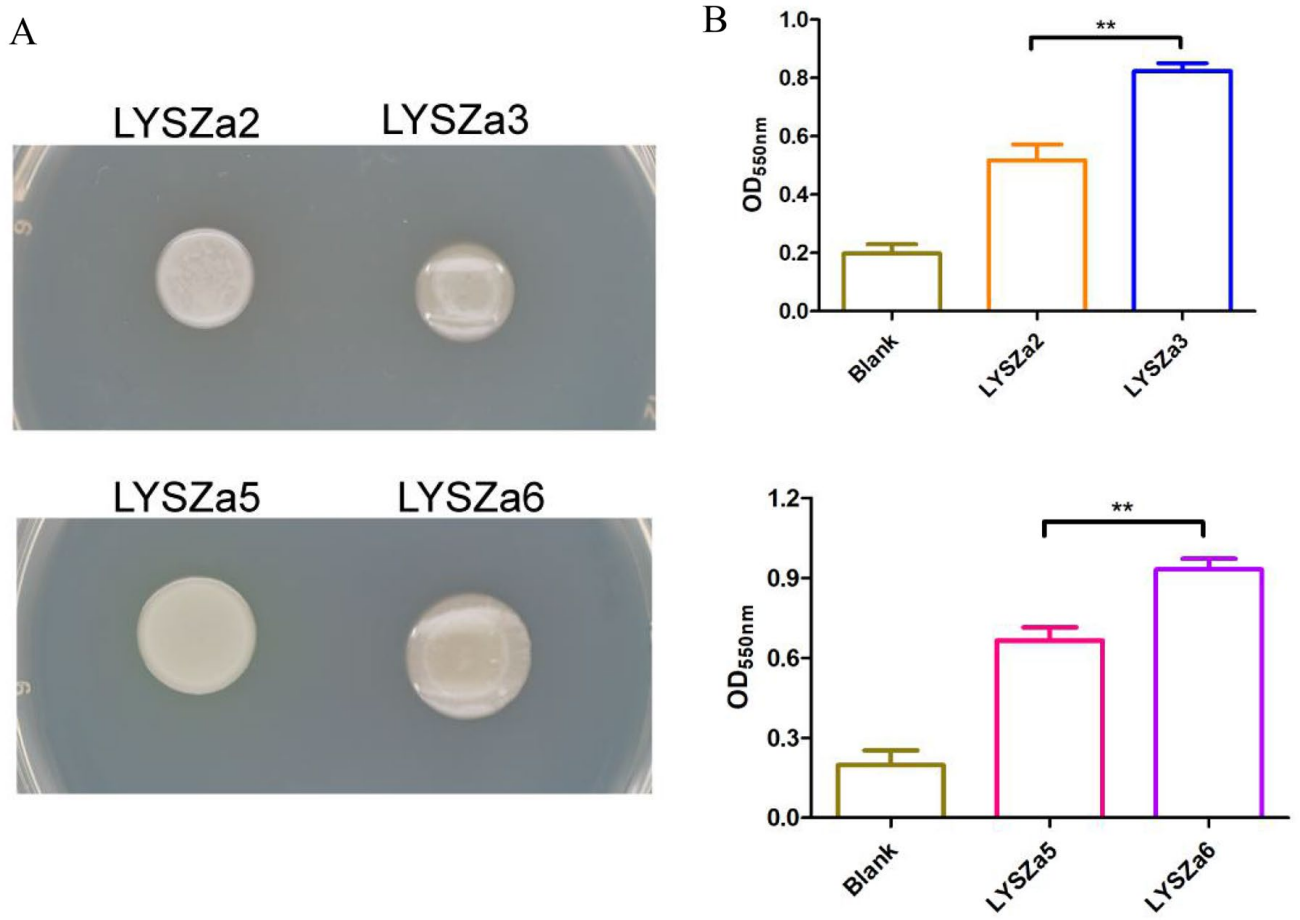
Phenotypic analysis. **A** Colony morphologies of the isolates; **B** Biofilm quantification tests by crystal violet staining, **p-value < 0.05; Upper panel: biofilm quantification test of LYSZa2 and LYSZa3, lower panel: biofilm quantification test of LYSZa5 and LYSZa6

In accordance with the gene expression analysis, the progeny isolates produce excessive alginate and exhibit more mucoid phenotype after in vitro cultivation on agar plates (Fig. 5A). As alginate plays an important role in biofilm architecture and development [23], we thus quantified biofilm formation of the isolates in vitro by crystal violet staining method. As seen from Fig. 5B, aligned with gene expression data, an increase in the biofilm formation was observed from LYSZa3 and LYSZa6 compared to LYSZa2 and LYSZa5 respectively.

### Suppression of T6SS of *P. aeruginosa* during adaptation in COVID-19 patients

T6SS is used as a weapon by *P. aeruginosa* to compete with neighboring bacteria cells and to combat with the host cells in order to gain surviving advantage in the polymicrobial environment in the respiratory system. Suppression in T6SS would reduce its advantage to outcompete other bacteria and attenuate its virulence to escape from host immune clearance. We thus performed in vitro bacterial competition test and ex vivo cytotoxicity assay of the isolates to confirm the changes in the anti-prokaryotic and anti-eukaryotic capability between the ancestry isolates and the progeny isolates.

**Fig. 6:**
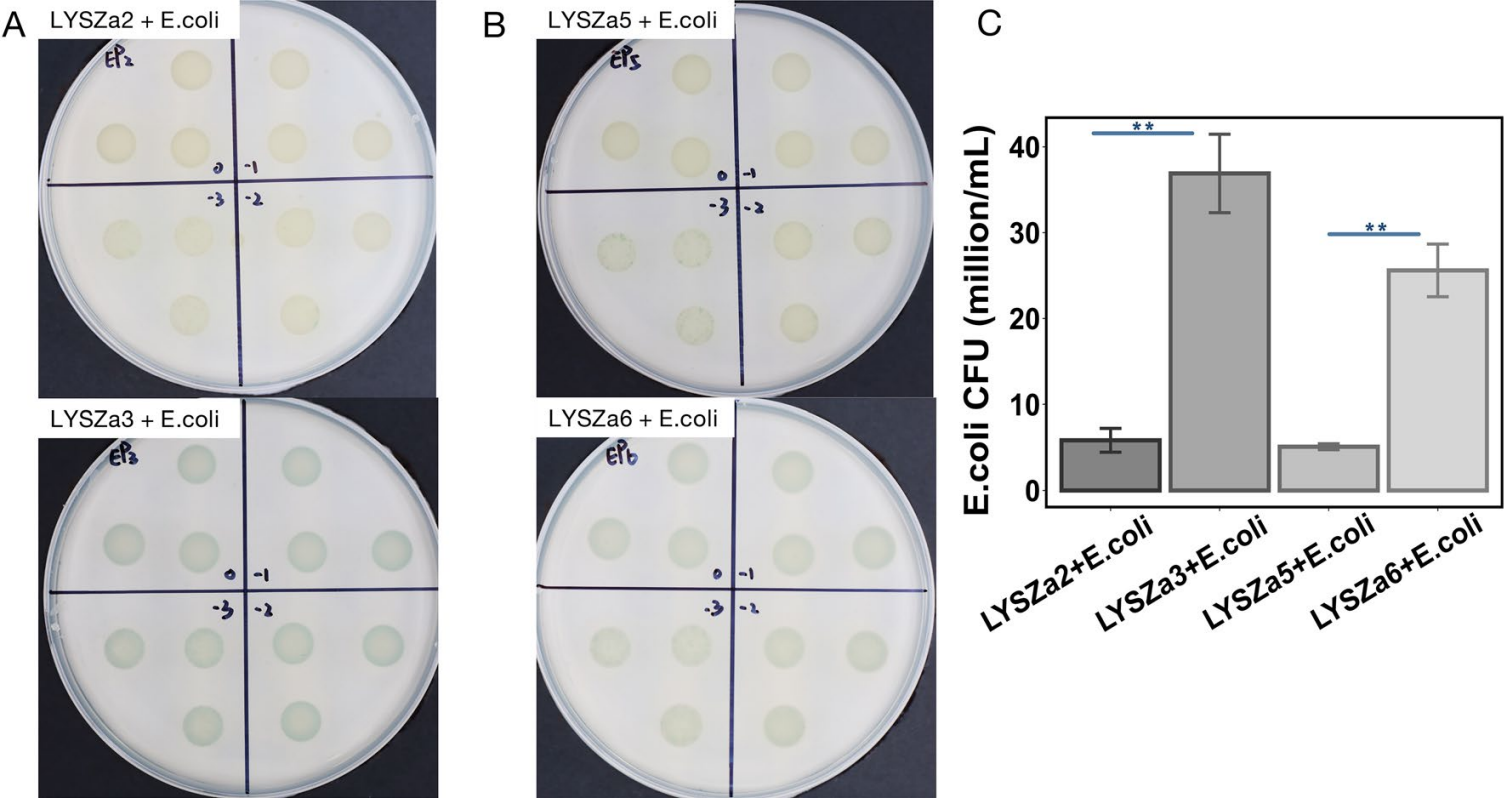
Bacterial competition assay between the isolates and *E. coli.* Blue pigment intensity indicates the survival of *E. coli.* **A** Upper panel: competition between LYSZa2 and *E. coli*; lower panel: competition between LYSZa3 and *E. coli*, number in each section denotes the dilution factor, ranged from 10^0^ to 10^–3^; **B** Upper panel: competition between LYSZa5 and *E. coli*; lower panel: competition between LYSZa6 and *E. coli*, number in each section denotes the dilution factor, ranged from 10^0^ to 10^–3^; **C** Counts of *E. coli* CFU left in each competition, **p-value < 0.05

We firstly examined the capability of the *P. aeruginosa* isolates in killing the model pray organism *E. coli* through bacterial competition assay. The *P. aeruginosa* isolates were mixed and cultured with *E. coli/placZ* at 1:1 ratio respectively. The mixtures were diluted to 10^–3^ while triplicates of each dilution were then spotted and cultured on plates containing X-gal to test the killing efficiency (Fig. 6A and B). *E. coli/pLacZ* strain was able to digest X-gal and appeared as blue colonies on plates. Survival of *E. coli* was determined both qualitatively by visualizing the intensity of blue pigment of the colonies and quantitatively by counting the blue *E. coli* CFU left. As observed from Fig. 6A and B, almost no trace of blue pigment could be seen from the mixture of *E. coli* and the ancestry *P. aeruginosa* isolates, LYSZa2 and LYSZa5. More intensive blue colonies were observed from the mixed cultures of *E. coli* and the progeny isolates, LYSZa3 and LYSZa6, indicating disadvantage of the progeny isolates to outcompete *E. coli* compared to their respective ancestry isolates*.* Same trend was also observed from quantitative tests illustrated in Fig. 6C where higher numbers of *E. coli* CFU were left after coculturing with LYSZa3 and LYSZa6 comparing with those culturing with LYSZa2 and LYSZa5 respectively. Such results indicated a decrease in the bactericidal activity of the progeny isolates during bacterial competition consequently due to the inhibition of T6SS.

**Fig. 7:**
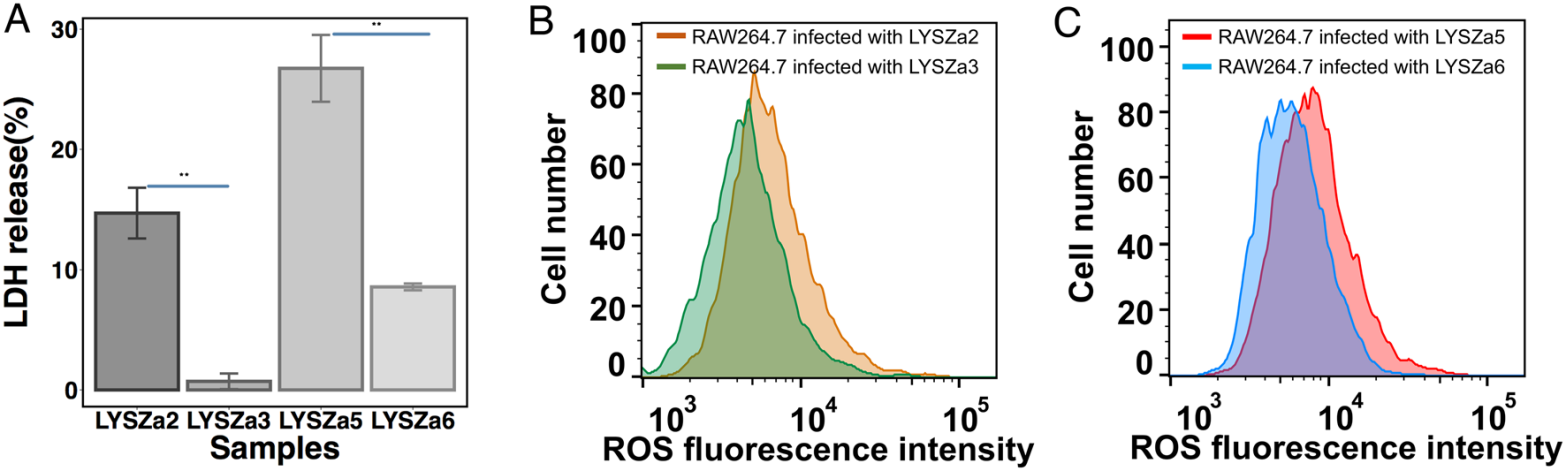
Macrophage cytotoxicity and ROS production of the ancestry isolates and the progeny isolates. **A** The cytotoxicity effect against macrophage cells of LYSZa2, LYSZa3, LYSZa5 and LYSZa6 is illustrated by the percent of LDH released, **p-value < 0.05. **B** The intracellular ROS levels in RAW264.7 cells at 3 h after phagocytosis of LYSZa2, LYSZa3 and LYSZa5, LYSZa6 (**C**)

We then performed ex vivo macrophage killing assay to assess the *P. aeruginosa* cytotoxicity against the eukaryotic cells. Cultures of the *P. aeruginosa* isolates were added to infect RWA264.7 macrophages individually. Relative lactate dehydrogenase (LDH) release was measured to determine the death of macrophages. As illustrated in Fig. 7A, LDH released by macrophages infected by LYSZa3 was much lower than that of LYSZa2. Similar results were observed from LYSZa5 and LYSZa6 where macrophages infected by LYSZa5 released a significant higher level of LDH comparing to LYSZa6. Thus, both LYSZa3 and LYSZa6 strains possess weaker cytotoxicity comparing to their ancestral isolates. Macrophages play an important role in the defense against the invading pathogens in the host, the reactive oxygen species (ROS) produced by macrophages within phagolysosome contributes to the destruction and clearance of pathogens [24]. Next, we have measured the intracellular ROS production in macrophages after 3 h infection of LYSZa2, LYSZa3, LYSZa5 and LYSZa6. We found that the ROS level within the macrophages which infected with the progeny strains were lower than the ancestor strains (Fig. 7B and C). Such results suggested that these progeny isolates possess attenuated virulence towards their eukaryotic hosts after short period of adaptation in the COVID-19 patients.

## Discussion

It is well known that secondary bacterial infection after virus (e.g. influenza) infection will further increase morbidity and mortality due to virus infection. Even though a lot of knowledge regarding the lung immunology and SARS-CoV-2 virus pathogenesis has been provided recently, the increased occurrence of illness and death due to bacterial superinfection and its complications indicated that there is a much underestimated and neglected influence of bacterial superinfection on the disease progression in COVID-19 patients. In this study, we isolated the ancestor and the progeny isolate pairs of hospital-acquired *P. aeruginosa* strains from two critically ill COVID-19 patients to investigate its adaptive evolution during superinfection with SARS-CoV-2 virus. Our results suggested that *P. aeruginosa* upregulates its alginate biosynthesis and downregulates its T6SS to persist in the respiratory system and reduce its virulence to escape from host clearance for longer colonization. To the best of our knowledge, this is the first study describing the adaptive evolution of *P. aeruginosa* superinfection within the COVID-19 patients, which suggests that SARS-CoV-2 virus infection could provide a lung environment that allows rapid change of bacterial pathogens with genetic traits suitable for chronic infections.

The *P. aeruginosa* isolates collected from the two patients belongs to the same MLST type. In addition, the two patients lived in the same ward during hospitalization, they are therefore hospital-acquired *P. aeruginosa* strain. Genomic sequencing analysis revealed that the genomes of the isolates carry specific GIs for DNA repair and protein secretion systems, and ARGs for multiple drug classes. Common genomic modification events identified between the progeny isolates and the ancestral isolates indicated the genomic changes in genes related to T6SS, *tla3* and *tli5b3*. Through differential gene expression analysis, we found that genes involved in alginate biosynthesis were upregulated greatly while genes involved in T6SS protein secretion system were significantly downregulated. Alginate is an essential EPS component converting non-mucoid *P. aeruginosa* to mucoid phenotype, and is important for biofilm structure and antimicrobial resistance [23]. Clinical *P. aeruginosa* strains overproducing alginate isolated from CF patients enhanced biofilm formation leading to the aggravation of disease conditions and poor prognosis [25, 26]. Moreover, excessive biosynthesis of alginate in *P. aeruginosa* promotes its superinfection with other pathogens such as *S. aureus* and *B. cenocepacia* and increases their persistence in CF infections [27, 28]. Overproduction of alginate and increased biofilm formation were observed in the progeny strains in this study, inferring the potential mechanism adopted by *P. aeruginosa* in COVID-19 patients to enhance colonization and promote superinfection. As mucoid *P. aeruginosa* upon overexpression of alginate recalcitrating against antibiotics is well described [23, 29, 30], thick alginate layer is also probably a key contributing factor to the increase in the antimicrobial resistance of the progeny isolate observed. Excessive alginate may also facilitate coexistence of *P. aeruginosa* and other superinfecting pathogens for secondary bacterial infection, and alter the microecological environment in the respiratory system of COVID-19 patients. Further analysis is needed for deeper investigation on the role of alginate in the microbial community in COVID-19 patients.

A more notable observation is the attenuation of T6SS in the *P. aeruginosa* isolates during short term evolution in COVID-19 patients. The expression of genes involved in T6SS were significantly downregulated in both the progeny isolates. *P. aeruginosa* T6SS functions as a phage-like toxin delivery apparatus to transport toxic effectors into surrounding competing bacterial cells and host cells to gain survival advantages and infect host cells [31]. *P. aeruginosa* carries three types of T6SS, being HSI-I, HSI-II, and HSI-III respectively. HSI-I is dedicated to inter-bacterial competition while HSI-II and HSI-III associate with both anti-prokaryotic and anti-eukaryotic functions [32, 33]. All of these three gene clusters contain *hcp*, *clpV*, *vgrG*, *tss/hsi* genes and other genes including *ppkA, pppA, tag, sfa*, *fha,lip, dotU, icmF*, *stp* and *stk* genes for the full function of T6SS apparatus (Fig. 4) [31, 34]. In both of the *P. aeruginosa* progeny isolates, especially in LYSZa6, the expression of most of HSI-II genes and associated genes were downregulated significantly (Fig. 4). Such results indicated a general inhibition of HSI-II T6SS in the *P. aeruginosa* isolates during adaptation and evolution in the patients to adapt to COVID-19 environment. As HSI-II T6SS associates with anti-bacterial and anti-host functions, we tested both the bacterial killing capacity and macrophage cytotoxicity of the isolates. In addition to T6SS, T3SS is another key virulence regulating factor in *P. aeruginosa*, translocating exotoxins to infect host cells. Our RNA-Seq and RT-PCR results suggested a low and unchanged T3SS activity in all the isolates, indicating minimal contribution from T3SS to the attenuation of virulence of the progeny isolates to host cells. Moreover, previous study has indicated that *P. aeruginosa* T3SS associates with its planktonic phenotype and acute infection while T6SS is associated with biofilm phenotype and chronic infection [35]. Our results showed *P. aeruginosa* forms more biofilm through upregulation of alginate biosynthesis and reduced virulence through attenuation of T6SS. These results confirmed the decrease in the virulence to both neighboring bacteria and host cells of the progeny isolates due to T6SS attenuation. Previous studies had demonstrated that *P. aeruginosa* abrogated its T6SS to adapt to the host environment by genetic modification on genes such as *vgrG* and *vgrG4* during chronic infection of cystic fibrosis [17, 36]. Here we showed that *P. aeruginosa* attenuated its T6SS at transcriptional level to gain fitness and survival advantages during acute infection of COVID-19 pneumonia.

The attenuation of T6SS in *P. aeruginosa* may give opportunities to other bacterial species to thrive in the host lung. A recent research showed that *P. aeruginosa* abrogating its T6SS could be outcompeted by coinfecting *B. cenocepaica* in CF patients in an age- and T6SS-dependent manner and predisposing host to superinfection of *B. cenocepacia* [37]. Thus, *P. aeruginosa* attenuating its T6SS in COVID-19 patients could probably play a pivotal role in coexisting with other pathogenic strains in the polymicrobial environment and promoting subsequent infection in COVID-19 patients. However, metagenomics analysis of the sputum of the two patients in which the *P. aeruginosa* isolate LYSZa2, LYSZa3, LYSZa5, LYSZa6 were sampled indicated that there is no change in the microbial diversity during the short colonization period of *P. aeruginosa* (Additional file 1: Fig. S1). Moreover, *P. aeruginosa* adaptation described in this study is rather disease-specific since it adapts to the lung environments differently in other disease such as cystic fibrosis and ventilated associated pneumonia. Previous studies have shown that *P. aeruginosa* altered its quorum sensing systems and cell wall component (*lasR, mpl*) to reduce its virulence during adaptation in the airway of VAP patients[16]; while this bacterium slowly adapts to the airway of CF patients by genetic mutations in its *mucA*, *algU*, *vgrG* and etc. genes for chronic infection [17, 38]. To the best of our knowledge, the attenuation of *P. aeruginosa* HSI-II T6SS gene expression in COVID-19 patients has not been reported in other studies. Moreover, isolates collected from different patients exhibited similar gene expression pattern. Thus, *P. aeruginosa* adaptation described in this study is rather disease-specific. Our results suggest that the attenuation of T6SS in *P. aeruginosa* in COVID-19 patients might gain some advantage in escaping from the host immune clearance, which needs further exploration.

In this study, we have also examined the microbiome in the lungs of the two COVID-19 patients enrolled in this study. Result of metagenomics analysis indicated that *P. aeruginosa* is the most abundant species in the lungs, followed by *Ralstonia pickettii*, *Cellvibrio japonicus*, *Botrytis cinerea* and *S. aureus*. The difference in the respiratory microbial composition between COVID-19 patients with and without VAP is insignificant while the dominating species are *Klebsiella spp., P. aeruginosa*, *S. maltophilia*, *S. aureus* etc. [39]. A study conducted on the core microbiome in the airways of patients with different pulmonary diseases including chronic obstructive pulmonary disease(COPD), exacerbated chronic obstructive pulmonary disease (ECOPD), sarcoidosis and interstitial lung diseases(ILD) showed that the most abundant taxa in each disease is *Reyranella*, *Serratia, Serratia* and *Haemophilus* respectively. Other dominating taxa include *Prevotella*, *Escherichia_Shigella*, *Streptococcus*, *Pseudomonas* and etc. with varied abundance in different diseases [40]. Another recent study analyzing the core microbiome in the airways of cystic fibrosis patients showed that *S. aureus*, *P. aeruginosa*, *Rothia mucilaginosa*, and *Prevotella melaninogenica* are the most abundant species [41]. By comparing the core airway microbiomes, the pattern of microbial composition in SARS-CoV-2 infected environment is distinct from those of other diseases. Moreover, there are researches focusing on the physiological environment in the respiratory systems of critically ill COVID-19 patients. Critically ill COVID-19 patient would develop symptoms like ground glass opacities, interstitial thickening, and coarse reticular pattern and so on in the lungs. SARS-CoV-2 virus binds to angiotensin converting enzyme 2 (ACE2) and alters renin-angiotensin system activities, thus leading to enhanced inflammation (increased levels of VEGF, IP-10/CXCL10, MCP-3/CCL7, IL-6, IL-1, ROS etc.) and development of pulmonary fibrosis [42]. The high neutrophil to lymphocyte ratio leads to the generation of high levels of ROS, which induces host immunopathological response and worsens the conditions of the critically ill COVID-19 patients [43]. In our previous study, we have found that COVID-19 patients infected with *P. aeruginosa* express higher levels of IL-6, CRP and PCT [22]. IL­6 is a key factor of the cytokine storm of critically ill COVID-19 patients and increased level of IL-6 associates with higher death rate of the patients [44, 45]. Studies on cystic fibrosis revealed that the levels of proinflammatory cytokines including IL-1, TNF, IL-6 and IL-8 are higher in CF patients while level of IL-10 is lower comparing to healthy individuals [46]. However, IL-6 levels in the sputum of patients with advanced CF lung disease was extremely low but normal systemic IL-6 production was found [44]. Another study found that early isolate of *P. aeruginosa* from the respiratory system of CF patients could induce the expression of IL-1β but minimal expression of TNF, IL-6 and IL-8; while inversely, chronic isolates could induce higher expression of TNF, IL-6 and IL-8 and low IL-1β production [47]. Moreover, measurement of circulating and sputum cytokines from CF patients with chronic *P. aeruginosa* infection revealed that higher levels of IL-1 receptor antagonist (IRAP), IL-lα, IL-1β, IL-6 and TNF-α could be detected from sputum, while very little circulating cytokines were detected [48]. In a mouse model with *P. aeruginosa* VAP, the expression of ICAM and VCAM in the lungs and TNF-α, IL-1β, and IL-6 levels in BALF of wild type mice increased significantly after being infected, while TNF-α is a key regulator of the lung injury caused by *P. aeruginosa* VAP [49]. Thus, comparing with other diseases like CF and VAP, COVID-19 patients retain a distinctive physiological environment in the respiratory system. Considering this together with the results of the present study, SARS-CoV-2 infections can create a specific lung environment that allows rapid evolution and selection of *P. aeruginosa* variants with chronic adapted phenotypes.

### Conclusions

In summary, we demonstrated that *P. aeruginosa* is able to colonize and evolve in SARS-CoV-2 infected environment by altering its alginate biosynthesis and T6SS gene expression, which might enhance biofilm formation, increase antimicrobial resistance and reduce host immune attack during the COVID-19 patients. Our study suggests that post SARS-CoV-2 bacterial superinfection allows the development of bacterial chronic colonizers, which can potentially spread in patients with chronic lung diseases.

## Methods

### Isolate collection, bacterial strains and growth media

Four isolates of *P. aeruginosa* were collected at two different time points longitudinally from sputum samples or BALF of two critically ill COVID-19 patients respectively during routine clinical tests. Isolates collected from patient 1 were named as LYSZa2 and LYSZa3 while isolates collected from patient 2 were named as LYSZa5 and LYSZa6. Luria–Bertani (LB) broth and ABTGC medium supplemented with 10% Tryptic Soy Broth (TSB) were used for growing cultures. ABTGC medium consists of 0.1% MgCl_2_, 0.1% CaCl_2_, 0.1% FeCl_3,_ 0.2% glucose, 0.2% casamino acids and 10% A10 medium which is made of 15.1 mM (NH_4_)_2_SO_4_, 33.7 mM Na_2_HPO_4_·2H_2_O, 22 mM KH_2_PO_4_ and 0.05 mM NaCl.

### Antimicrobial susceptibility tests

Antimicrobial susceptibility of the isolates to various antibiotics, including ceftazidime, piperacillin, cefoperazone/sulbactam, imipenem, aztreonam, and levofloxacin, were performed using the Kirby-Bauer disc-diffusion method. Diameters of inhibition zone was measured using a Vernier caliper. Susceptibility was determined according to CLSI 2019 [50]. Results of antibiotic susceptibility tests were listed in Additional file 3: Table S1.

### Biofilm formation assay

The isolates were cultured in LB broth at 37 °C for overnight. The overnight cultures were diluted to OD_600nm_ 0.01 in fresh LB broth. 100 µL of the diluted cultures were loaded into 96-well plate in triplicates and incubated for 24 h at 37 °C statically allowing the formation of biofilm. After removing spent media, biofilms were washed carefully with ddH_2_O for two times. Biofilms were then stained by 125 µL of 0.1% crystal violet (CV) with 15 min incubation at room temperature. CV stain in the wells was discarded while stained biofilms were washed twice thoroughly with ddH_2_O and air-dried. Biofilms were then dissolved into 125 µL of 30% acetic acid and quantified relatively by measuring OD_550nm_ values on a Tecan infinity pro200 microplate reader.

### Genome extraction and sequencing

The isolates were cultured in LB broth at 37 °C to early stationary phase. For Illumina sequencing, genomic DNA of each isolates was extracted using AxyPerp Bacterial Genomic DNA Miniprep Kit (Corning, New York, USA) following manufacturer’s protocol. PCR-free libraries were constructed using VAHTSTM PCR-Free DNA Library Prep Kit for Illumina® (Vazyme, China) following standard protocol. VAHTSTM DNA Adapters for Illumina® (Vazyme, China) was used to tag adaptor to purified fragments. Quality of the libraries were assessed by Agilent Technologies 2100 Bioanalyzer and qPCR. Paired-end DNA sequencing was then performed on Illumina HiSeq X platform with read length of 150 bp. For PacBio sequencing, genomes of LYSZa2 and LYSZa5 were extracted using Mabio Bacterial DNA Extraction Mini Kits (Mabio) according to manufacturer’s protocol. DNA fragmentation was done using G-tubes (Covaris). SMRTbell DNA template libraries were prepared according to the manufacturer’s specification (PacBio, Menlo Park, USA). DNA sequencing was then performed on Pacific Biosciences RSII sequencer (PacBio, Menlo Park, USA).

### Transcriptome extraction and sequencing

The isolates were cultured in triplicates in LB broth at 37 °C to early stationary phase. Magen HiPure Universal RNA Mini kits (MCBio, China) was used to extract total RNA following the manufacturer’s protocol. Extracted RNA was quantified using Qubit 2.0 (Thermo Fisher Scientific, MA, USA) and Nanodrop One (Thermo Fisher Scientific, MA, USA). Quality of RNA samples was assessed by Agilent 2100 system (Agilent Technologies, Waldbron, Germany). RNA libraries were constructed according to standard protocol using NEB Next® Ultra™ Directional RNA Library Prep Kit for Illumina® (New England Biolabs, MA, USA). Ribosomal RNA depletion was carried out using Ribo-zero rRNA Removal Kit. cDNA was synthesized using NEB Next First Strand Synthesis Reaction Buffer. Paired-end RNA sequencing was performed on Illumina NovaSeq 6000 platform with read length of 150 bp.

### Sequencing data analysis

Genomic Illumina sequencing reads were imported to CLC Genomics Workbench 20 (Qiagen) and preprocessed using Adaptor trimming function to remove failed reads and adaptor sequences. Clean reads were then assembled into contigs using De Novo Assembly module of CLC Genomics Workbench 20 with default parameters. Single nucleotide polymorphism was detected using ‘Resequencing’ module of CLC Genomics Workbench 20 based on frequency of more than 80% using *P. aeruginosa* PAO1 genome (NC_002516.1) as reference. PacBio sequencing reads were assembled into draft genome using HGAP4 pipeline of SMRT Link software v9.0 with default settings. Rearrangements of draft genomes were checked using Mauve software v.2.4.0 by PROGRESSIVEMAUVE alignment mode [51]. Multilocus sequence typing (MLST) was performed using MLST service available on the Center of Genomic of Epidermiology (CGE) webserver [52]. Identification of antimicrobial resistance genes was performed using ResFinder service on CGE webserver based on 85% of identity and 60% of minimal length [53]. Phylogenetic tree was constructed using Parsnp under libMUSCLE alignment mode using draft genomes of the isolates and other strains to construct the core genome tree [54]. Genomic islands on LYSZa2 and LYSZa5 genomes were predicted using IslandViewer 4 webservice using *P. aeruginosa* as reference strain [55]. Circular plot was constructed using BLAST Ring Image Generator using the genomes of *P. aeruginosa* isolates and other *P. aeruginosa* strains downloaded from NCBI database [56]. Genomic loci were visualized using Easyfig package using default setting [57]. Gene names and locus tags labelled on the genomic loci were obtained from Pseudomonas Genome Database [58]. Prediction of protein functions of the genes was performed using NCBI Prokaryotic Genome Annotation Pipeline (PGAP) [59].

RNA sequences were pre-processed, mapped to *P. aeruginosa* PAO1 reference genome and analyzed using RNA analysis module of CLC Genomics Workbench 20 (Qiagen) with default parameters. Total read counts of each sample were normalized and compared using DESeq2 R package. Differentially expressed genes (DEGs) were selected based on absolute fold change > 4, adjusted p-value < 0.05 and base mean > 20 [60]. GO enrichment analysis of DEGs was performed on DAVID bioinformatics database v6.8 [61]. PCoA plot and heatmap were drawn using Vegan, ggplot2, and pheatmap packages in R 4.0.0.

### Bacterial competition assay

Competition between *P. aeruginosa* and *Escherichia coli* was carried out following the steps described previously by Hachani et al. [62]. Briefly, the isolates were cultured overnight at 37 °C on LB agar plates. *E. coli/pLacZ* strain was grown on LB agar plates containing 40 mg/mL of 5-bromo-4-chloro-indolyl-β-d-galactopyranoside (X-gal) at 37 °C for overnight and appeared blue in colour. Single colony of each strain was subcultured in TSB medium and grown under agitation for overnight. Appropriate volume of each overnight culture was taken to make final OD600nm = 1 and centrifuged to collect cells. Cell pellets were resuspended in 100 µL TSB medium while 10 µL of each suspension was spotted and incubated on LB agar plate for 5 h at 37 °C. 30 µL of cell suspension of each *P. aeruginosa* strain was taken and mixed gently with 30 µL of *E. coli* cell suspensions. 20 µL of mixed cell cultures were spotted onto LB agar plate and incubated for 5 h at 37 °C. Each bacterial spot was scraped from LB agar plates and resuspended in 1 mL of TSB medium. Resuspensions of bacterial spots were diluted to 10^–3^ serially in tenfold dilutions. These serial cell dilutions were then spotted in triplicates onto LB agar plates containing 40 mg/mL X-gal and incubated for 8 h for killing *E. coli*. 100 µL of 10^–3^ dilution of each mixed bacterial spot were plated onto LB agar plates containing 40 mg/mL X-gal for CFU counting of *E. coli* for quantitating killing effect.

### Cytotoxicity assay

The cytotoxicity of the isolates was assayed by using murine RAW 264.7 macrophages. RAW macrophages were grown in 24-well plates in Dulbecco modified Eagle medium (DMEM), GlutaMAX, sodium pyruvate, and phenol red supplemented with 10% FBS. Prior to infection, confluent RAW cells were washed twice with sterile PBS and incubated in DMEM medium. Log-phase cultures were washed with sterile PBS twice, and resuspended in DMEM medium devoid of FBS. Macrophage cells were infected with the isolates respectively at a Multiplicity of Infection (MOI) of 20 at 37 ℃ in 5% CO_2_ incubator. After 3 h infection, the culture supernatants were collected for detecting lactate dehydrogenase (LDH) activities. The LDH activities were detected by using commercially LDH cytotoxicity kit (YAESEN Bio) according to standard procedure.

### ROS production measurement

ROS production level in RAW264.7 cells cultured for 4 h after phagocytosis was detected by Reactive Oxygen Species Assay Kit (Beyotime). Briefly, RAW264.7 cells were infected with the isolates respectively at a Multiplicity of Infection (MOI) of 20 at 37 ℃ in 5% CO_2_ incubator. After 3 h infection, cells were washed with PBS and incubated in DMEM medium without FBS and DCFH-DA (final concentration 10 μM) reagent at 37 °C for 20 min, as per the manufacturer’s protocols. The cells were washed with PBS and harvested. The stained cells were analysed by using a Cytoflex S flow cytometer (Beckman). All samples were assayed with lasers emitting at 488 nm, and the fluorescence was collected by 530/30 nm bandpass filter. The flow cytometric data were analysed using FlowJo software (BD Biosciences).

### Metagenomics analysis

Nucleic acid of 4 sputum samples (two different time points of two critically ill COVID-19 patients) was extracted and complementary DNA (cDNA) was generated from RNA template by reverse transcription. DNA libraries were constructed through DNA‐fragmentation, end‐repair, adapter‐ligation, and PCR amplification. Qualified libraries were sequenced by BGISEQ‐50 platform [63]. High‐quality sequencing data were generated by removing low‐quality and short (length < 35 bp) reads, followed by removal of human host sequences mapped to the human reference genome (hg19) using Burrows-Wheeler Alignment [64]. The detailed information of metagenomics data was displayed in Additional file 11: Table S9. The remaining clean data were assembled using the MEGAHIT (version 1.2.9) with default parameters. The open reading frames (ORFs) prediction was then conducted for assembled contigs using Prokka (version 1.12). CD-HIT (version 1.12) was used to cluster genes from each sample based on the parameters (identity > 95%, coverage > 90%). We aligned high-quality reads against the gene catalog using Salmon v1.2.1 (identity cutoff ≥ 95%) and calculated the corresponding relative abundance of each gene. The taxonomic composition were classified by Kraken 2 [65] by aligning to three Microbial Genome Databases, consisting of 14,459 viruses, 62,319 bacteria, and 1587 fungi. Averages and standard deviations were computed using the base function in R 3.6.2. Venn diagrams were drawn with the Venn Diagram package, while heatmaps were generated using the pheatmap package by R 3.6.2. The α-diversity based on Shannon index on the species in each sample was calculated to evaluate the species diversity by R 3.6.2. Principal Coordinates Analysis (PCoA) was plotted based on Bray–Curtis dissimilarity to compare the species composition of the samples using Vegan package on R 3.6.2.

### RT-PCR

RNA was extracted from the four *P. aeruginosa* isolates using Qiagen RNeasy Mini Kit according to the manufacturer's protocol. After measuring RNA concentration, RNA was quickly converted to cDNA through reverse transcription following the standard steps of HiScript III All-in-one RT SuperMix Perfect for qPCR (Vazyme). RT-PCR was performed using a PowerUpTM SYBRTM Green Master Mix (Thermofisher) according to the manufacturer's protocol. Primers and probes were designed to target *P. aeruginosa* (GCA_000006765.1 ASM676v1). Primers used here are listed in Additional file 12: Table S10. Samples of total DNA from *P*. *aeruginosa* were diluted to 10 ng/μL. 10 µL of the total PCR volume was used according to the manufacturer’s protocol. The following PCR protocol was used: one cycle at 50 °C for 2 min and 95 °C for 2 min, followed by 45 cycles at 95 °C for 15 s, 57 °C for 15 s and 72 °C for 60 s. LightCycler 96 Software (Roche) was used for data analysis.

## Supplementary Information

**Additional file 1: Figure S1**. Metagenomic analysis of the sputum of the two patients in which *P. aeruginosa* isolates LYSZa2, LYSZa3, LYSZa5, and LYSZa6 were sampled indicated that there is no change in the microbial diversity. **(A)** Box plot showing variation in the abundance of top 20 species as determined by read abundance. **(B)** Stacked bar plot indicating the top 20 species in the four sputum samples as determined by taxonomic analysis. **(C)** Alpha diversity indicated by Shannon index within the two groups (A: Ancestor samples including LYSZa2 and LYSZa5; P: progeny samples including LYSZa3 and LYSZa6) and the statistics between the groups. The same letter means that there is no significant difference between the groups (Adjust p > 0.1).

**Additional file 2: Figure S2.** RT-PCR results of T3SS and T6SS genes. (A) RT-PCR results of the expression of selected T3SS and T6SS genes in LYSZa2 and LYSZa3; (B) RT-PCR results of the expression of selected T3SS and T6SS genes in LYSZa5 and LYSZa6. **: p-value < 0.01.

**Additional file 3: Table S1.** In vitro antimicrobial susceptibility tests of the isolates.

**Additional file 4: Table S2.** Predicted genomic islands on LYSZa2 genome by IslandViewer4 with at least one prediction method.

**Additional file 5: Table S3.** Predicted genomic islands on LYSZa5 genome by IslandViewer4 with at least one prediction method.

**Additional file 6: Table S4.** Genomic accession numbers of *P. aeruginosa* strains selected for phylogenetic tree construction.

**Additional file 7: Table S5.** Single nucleotide polymorphism (SNP) and other genome modifying events identified between the ancestry isolates and the progeny isolates using PAO1 as reference. *SNV* Single nucleotide variation, *MNV* Multi-nucleotide variation.

**Additional file 8: Table S6.** Full list of DEGs in LYSZa3 comparing to LYSZa2 selected based on the criteria of fold change ≧ 4, adjusted p-value < 0.05 and base mean ≧  20.

**Additional file 9: Table S7.** Full list of DEGs in LYSZa6 comparing to LYSZa5 selected based on the criteria of fold change ≧ 4, adjusted p-value < 0.05 and base mean ≧ 20.

**Additional file 10: Table S8.** Common DEGs identified in LYSZa3 and LYSZa6. Filtering criteria are fold change ≧ 4, adjusted p-value < 0.05 and base mean ≧ 20.

**Additional file 11: Table S9.** Detailed information of metagenomics data of the 4 respiratory samples.

**Additional file 12: Table S10.** Primers used for RT-PCR tests.

## Data Availability

All Illumina sequencing data used in this study could be found from BioProject No. PRJNA706783 and assembled genomes of *P. aeruginosa* LYSZa2 and *P. aeruginosa* LYSZa5 could be found from BioProject No. PRJNA712958 and PRJNA712961 on NCBI. The BGISEQ sequencing metagenomics data could be found from BioProject No. PRJNA772037.
