# Supplementary figures and images for: Pseudomonas aeruginosa modulates alginate biosynthesis and type VI secretion system in two critically ill COVID-19 patients
Source: Cell Biosci. 2022 Feb 9;12:14. doi: 10.1186/s13578-022-00748-z (PMC8827185; doi:10.1186/s13578-022-00748-z)

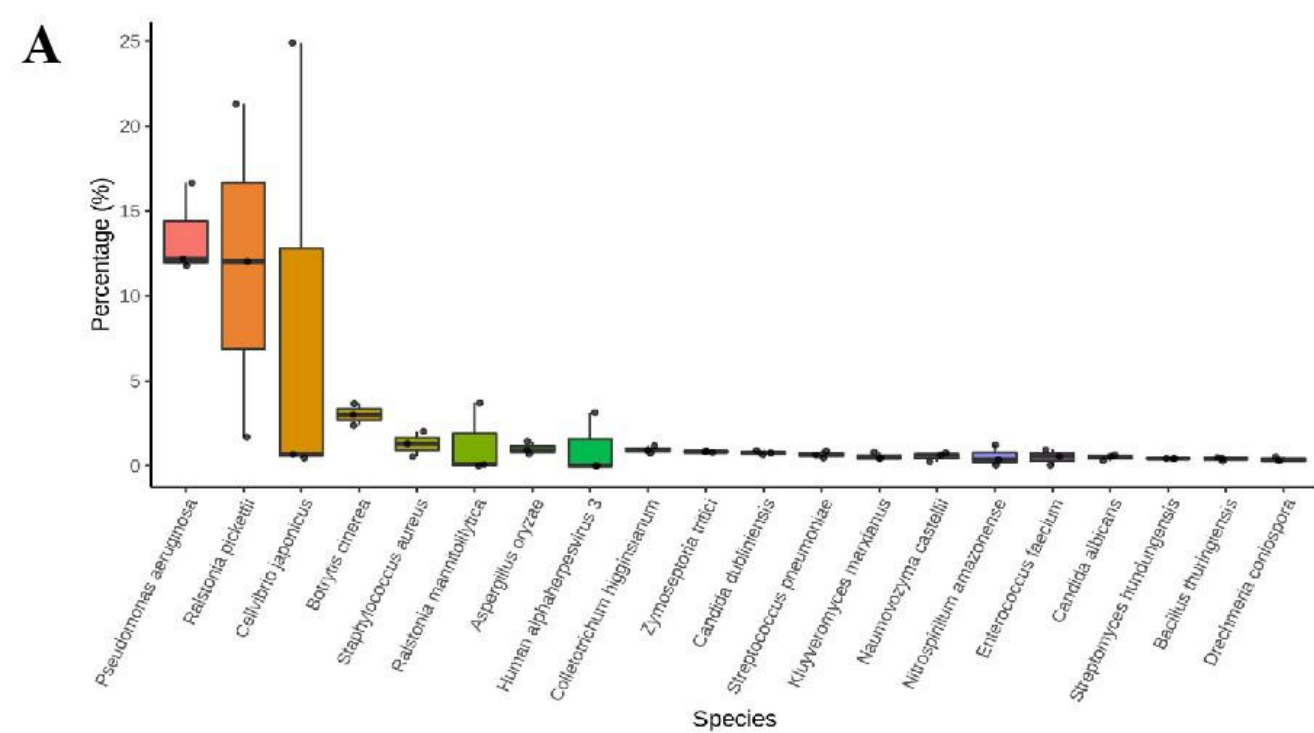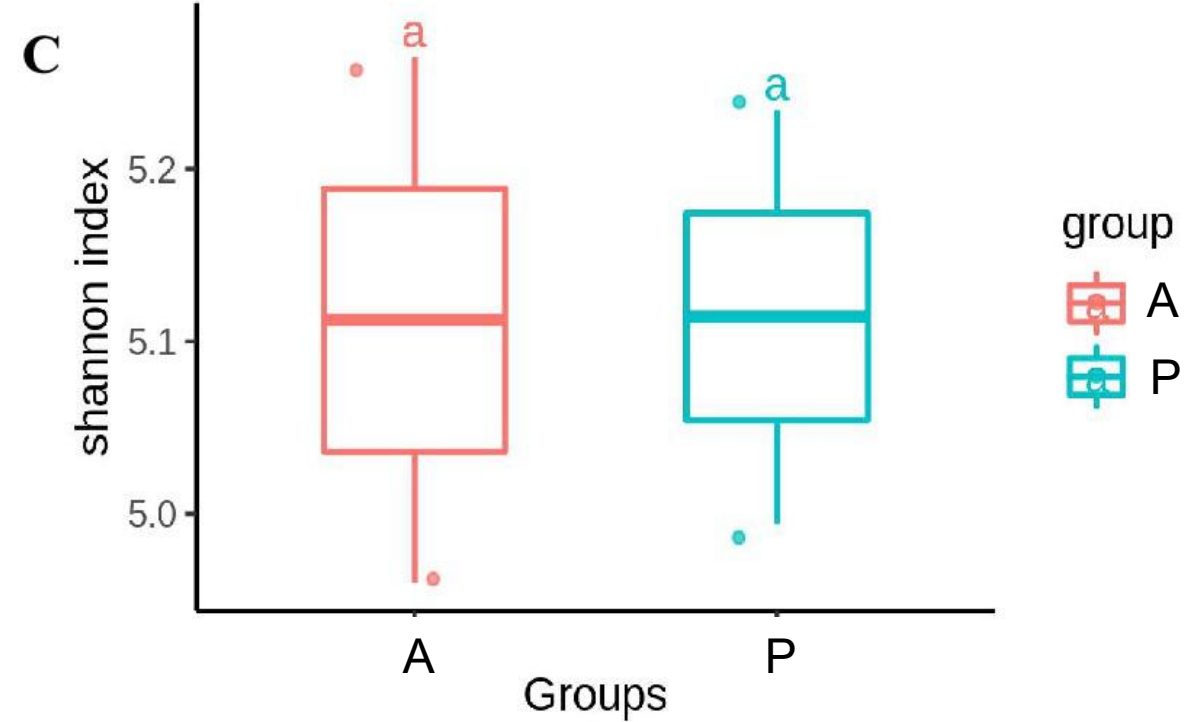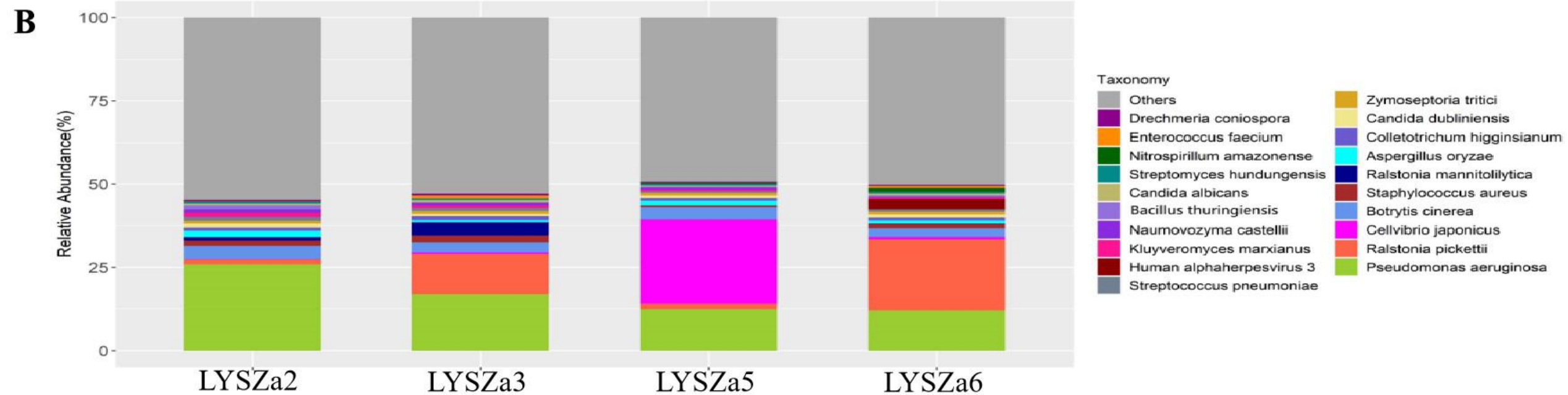

Supplement: Supplementary file 1 — Additional file 1: Figure S1. Metagenomic analysis of the sputum of the two patients in which P. aeruginosa isolates LYSZa2, LYSZa3, LYSZa5, and LYSZa6 were sampled indicated that there is no change in the microbial diversity. (A) Box plot showing variation in the abundance of top 20 species as determined by read abundance. (B) Stacked bar plot indicating the top 20 species in the four sputum samples as determined by taxonomic analysis. (C) Alpha diversity indicated by Shannon index within the two groups (A: Ancestor samples including LYSZa2 and LYSZa5; P: progeny samples including LYSZa3 and LYSZa6) and the statistics between the groups. The same letter means that there is no significant difference between the groups (Adjust p > 0.1). [file 13578_2022_748_MOESM1_ESM.pdf]

A

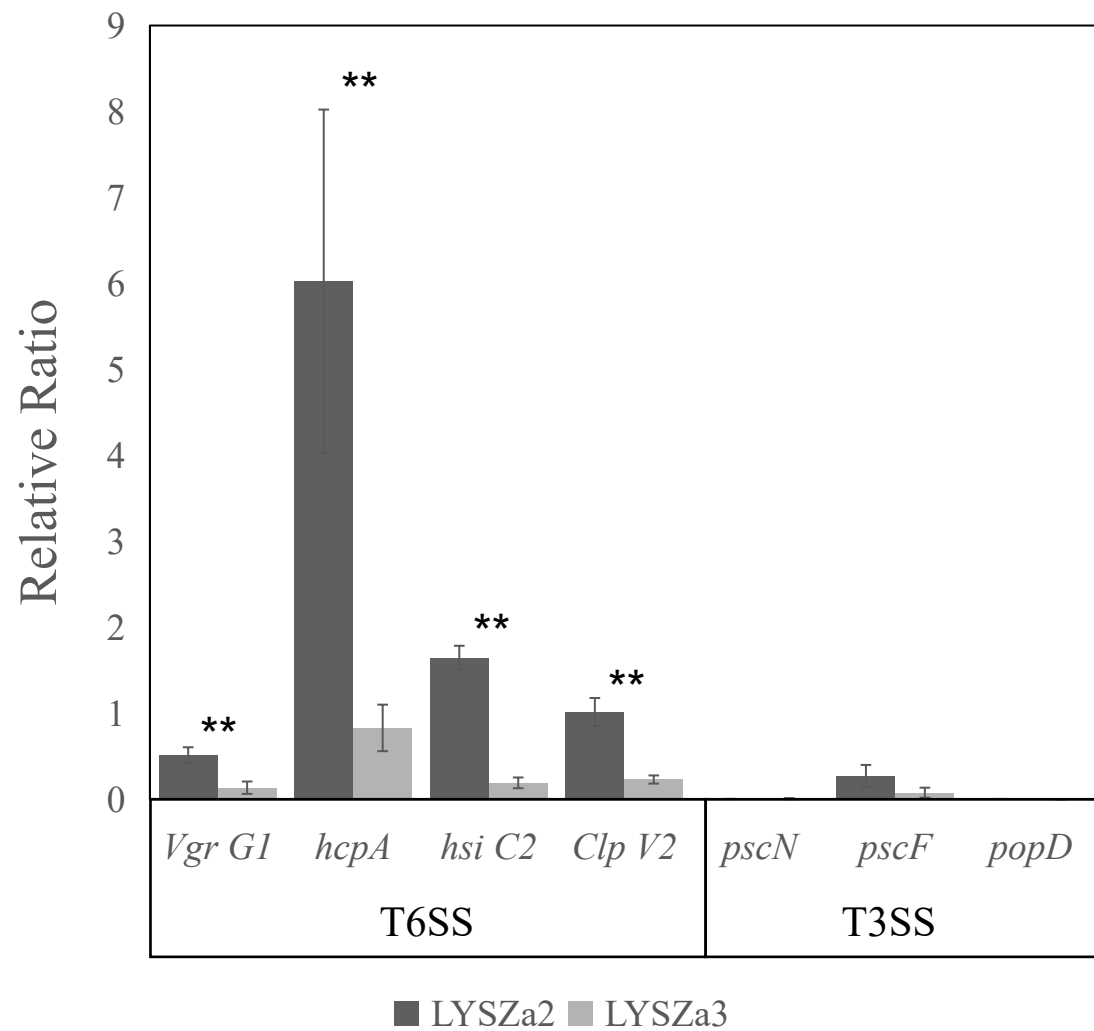

B

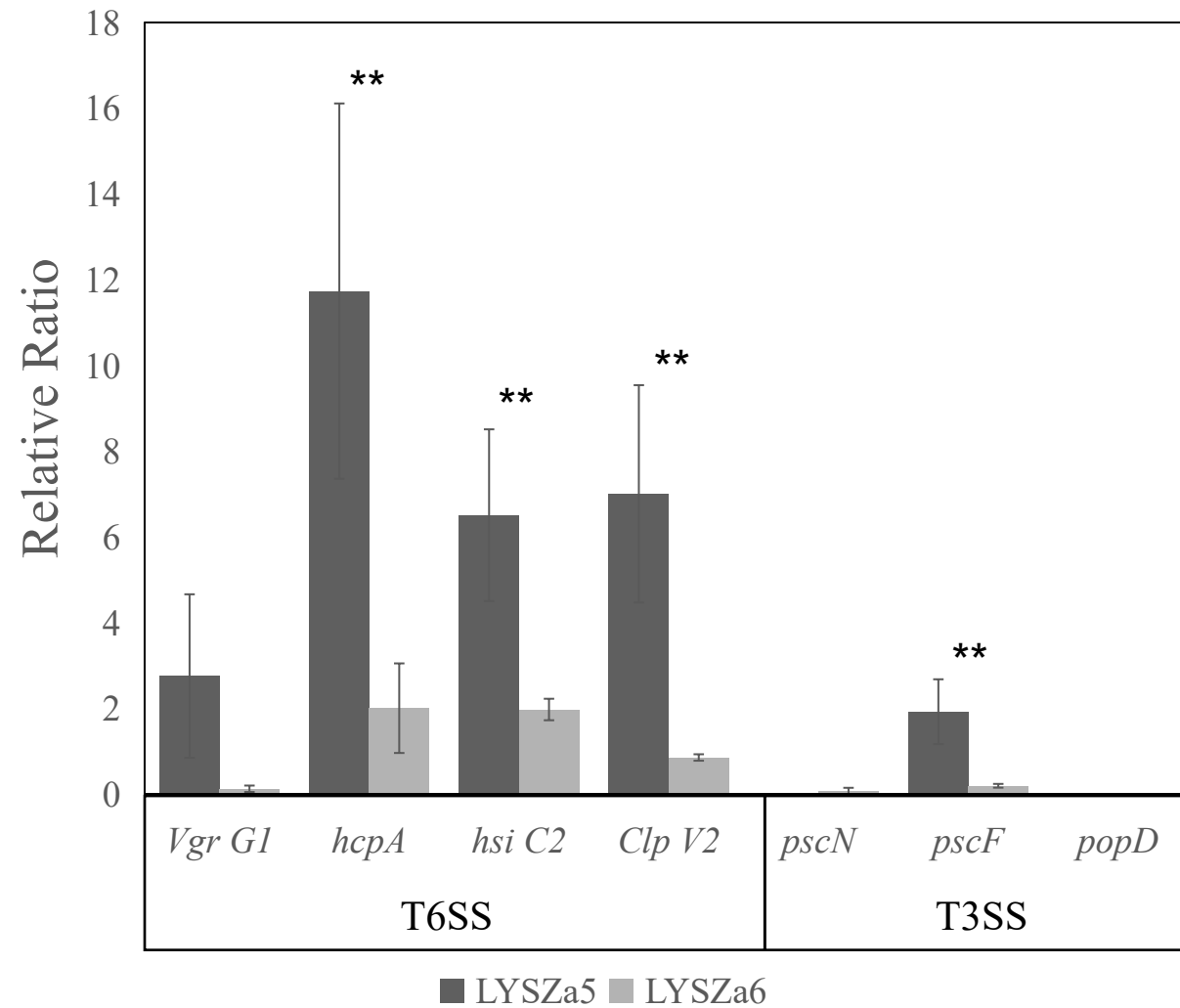

Supplement: Supplementary file 2 — Additional file 2: Figure S2. RT-PCR results of T3SS and T6SS genes. (A) RT-PCR results of the expression of selected T3SS and T6SS genes in LYSZa2 and LYSZa3; (B) RT-PCR results of the expression of selected T3SS and T6SS genes in LYSZa5 and LYSZa6. **: p-value < 0.01. [file 13578_2022_748_MOESM2_ESM.pdf]
