# Supplementary material for: Pseudomonas aeruginosa modulates alginate biosynthesis and type VI secretion system in two critically ill COVID-19 patients
Source: Cell Biosci. 2022 Feb 9;12:14. doi: 10.1186/s13578-022-00748-z (PMC8827185; doi:10.1186/s13578-022-00748-z)
Supplement: Supplementary file 3 — Additional file 3: Table S1. In vitro antimicrobial susceptibility tests of the isolates. [file 13578_2022_748_MOESM3_ESM.docx]

| **Antibiotics** | **LYSZa5** | **LYSZa6** | **LYSZa2** | **LYSZa3** |
| --- | --- | --- | --- | --- |
| ceftazidime-MIC(ug/ml ) | 2 S | 16 I | 2 S | 2 S |
| ceftazidime-KB | 25mm S | 17mm I | 24mm S | 24mm S |
| piperacillin-MIC(ug/ml ) | 8 S | 32 I | <=4 S | <=4 S |
| piperacillin-KB | 25mm S | 18mm I | 29mm S | 22mm S |
| cefoperazone/sulbactam-KB | 24mm S | 12mm R | 26mm S | 22mm S |
| imipenem-MIC(ug/ml ) | <=1 S | 8 R | <=1 S | <=1 S |
| imipenem-KB | 28mm S | 14mm R | 31mm S | 27mm S |
| aztreonam-MIC(ug/ml ) | 4 S | >16 R | 4 S | 4 S |
| aztreonam-KB | 25mm S | 6mm R | 27mm S | 20mm I |
| levofloxacin-MIC(ug/ml ) | 4 R | 2 I | 4 R | 4 R |
| levofloxacin-KB | 10mm R | 16mm I | 13mm R | 14mm R |

**Table S1.** *In vitro* antimicrobial susceptibility tests of the isolates. S:susceptible,I:intermediate,R:resistant.
