# Supplementary material for: Pseudomonas aeruginosa modulates alginate biosynthesis and type VI secretion system in two critically ill COVID-19 patients
Source: Cell Biosci. 2022 Feb 9;12:14. doi: 10.1186/s13578-022-00748-z (PMC8827185; doi:10.1186/s13578-022-00748-z)
Supplement: Supplementary file 4 — Additional file 4: Table S2. Predicted genomic islands on LYSZa2 genome by IslandViewer4 with at least one prediction method. [file 13578_2022_748_MOESM4_ESM.docx]

| **Island start** | **Island end** | **Gene start** | **Gene end** | **Strand** | **Product** |
| --- | --- | --- | --- | --- | --- |
| 436783 | 456906 | 436783 | 439674 | 1 | Ribonucleotide reductase of class Ia (aerobic), alpha subunit (EC 1.17.4.1) |
| 436783 | 456906 | 439937 | 441184 | 1 | Ribonucleotide reductase of class Ia (aerobic), beta subunit (EC 1.17.4.1) |
| 436783 | 456906 | 441619 | 443574 | -1 | hypothetical protein |
| 436783 | 456906 | 443574 | 445970 | -1 | hypothetical protein |
| 436783 | 456906 | 445987 | 446814 | -1 | Uncharacterized protein MSMEG_1245 |
| 436783 | 456906 | 446792 | 448525 | -1 | hypothetical protein |
| 436783 | 456906 | 448715 | 450253 | -1 | hypothetical protein |
| 436783 | 456906 | 450246 | 451154 | -1 | hypothetical protein |
| 436783 | 456906 | 453067 | 454095 | -1 | hypothetical protein |
| 436783 | 456906 | 454539 | 455735 | -1 | Mobile element protein |
| 436783 | 456906 | 456352 | 456906 | 1 | Prophage antirepressor |
| 609080 | 626249 | 608230 | 609096 | -1 | MaoC-like dehydratase |
| 609080 | 626249 | 609080 | 611227 | -1 | Acetyl-CoA synthetase (ADP-forming) alpha and beta chains, putative |
| 609080 | 626249 | 611337 | 612488 | -1 | Sterol carrier protein IgrF |
| 609080 | 626249 | 612485 | 612796 | -1 | conserved protein associated with acetyl-CoA C-acyltransferase |
| 609080 | 626249 | 613029 | 613829 | 1 | Transcriptional regulator, IclR family |
| 609080 | 626249 | 613869 | 614768 | -1 | Glycosyl transferase, family 2 |
| 609080 | 626249 | 615105 | 616790 | 1 | hypothetical protein |
| 609080 | 626249 | 617123 | 617857 | 1 | hypothetical protein |
| 609080 | 626249 | 618360 | 618605 | -1 | hypothetical protein |
| 609080 | 626249 | 618967 | 619905 | 1 | 5'-nucleotidase (EC 3.1.3.5) |
| 609080 | 626249 | 619909 | 620643 | -1 | hypothetical protein |
| 609080 | 626249 | 620636 | 621829 | -1 | Retron-type RNA-directed DNA polymerase (EC 2.7.7.49) |
| 609080 | 626249 | 622727 | 623437 | -1 | Phosphoribosylaminoimidazole-succinocarboxamide synthase (EC 6.3.2.6) |
| 609080 | 626249 | 623466 | 624176 | -1 | Metal-dependent hydrolases of the beta-lactamase superfamily I |
| 609080 | 626249 | 624163 | 625353 | -1 | Outer membrane beta-barrel assembly protein BamC |
| 609080 | 626249 | 625371 | 626249 | -1 | 4-hydroxy-tetrahydrodipicolinate synthase (EC 4.3.3.7) |
| 615229 | 622542 | 615105 | 616790 | 1 | hypothetical protein |
| 615229 | 622542 | 617123 | 617857 | 1 | hypothetical protein |
| 615229 | 622542 | 618360 | 618605 | -1 | hypothetical protein |
| 615229 | 622542 | 618967 | 619905 | 1 | 5'-nucleotidase (EC 3.1.3.5) |
| 615229 | 622542 | 619909 | 620643 | -1 | hypothetical protein |
| 615229 | 622542 | 620636 | 621829 | -1 | Retron-type RNA-directed DNA polymerase (EC 2.7.7.49) |
| 962334 | 991818 | 961792 | 962337 | -1 | Extracytoplasmic function (ECF) sigma factor VreI |
| 962334 | 991818 | 962334 | 963050 | -1 | Outer membrane TonB-dependent transducer VreA of trans-envelope signaling system |
| 962334 | 991818 | 963216 | 963536 | 1 | hypothetical protein in cluster with VreARI signaling system |
| 962334 | 991818 | 963654 | 964250 | -1 | Heme oxygenase HemO, associated with heme uptake |
| 962334 | 991818 | 964436 | 965107 | 1 | RecA/RadA recombinase |
| 962334 | 991818 | 965115 | 966530 | 1 | DNA polymerase IV-like protein ImuB |
| 962334 | 991818 | 966527 | 969601 | 1 | Error-prone repair homolog of DNA polymerase III alpha subunit (EC 2.7.7.7) |
| 962334 | 991818 | 975785 | 977014 | 1 | Exonuclease SbcD |
| 962334 | 991818 | 977023 | 980658 | 1 | Exonuclease SbcC |
| 962334 | 991818 | 980680 | 982845 | -1 | Exodeoxyribonuclease V alpha chain (EC 3.1.11.5) |
| 962334 | 991818 | 982842 | 986579 | -1 | Exodeoxyribonuclease V beta chain (EC 3.1.11.5) |
| 962334 | 991818 | 986576 | 990091 | -1 | Exodeoxyribonuclease V gamma chain (EC 3.1.11.5) |
| 962334 | 991818 | 990132 | 990815 | -1 | Lipoate-protein ligase A |
| 962334 | 991818 | 990928 | 991818 | -1 | Permease of the drug/metabolite transporter (DMT) superfamily |
| 1270608 | 1275520 | 1270748 | 1271614 | 1 | ParA-like protein |
| 1270608 | 1275520 | 1271616 | 1272353 | 1 | Orf50 |
| 1270608 | 1275520 | 1272350 | 1272841 | 1 | hypothetical protein |
| 1270608 | 1275520 | 1272838 | 1273500 | 1 | hypothetical protein |
| 1270608 | 1275520 | 1273497 | 1274180 | 1 | hypothetical protein |
| 1270608 | 1275520 | 1274180 | 1274881 | 1 | FIG004780: hypothetical protein in PFGI-1-like cluster |
| 1270608 | 1275520 | 1274878 | 1275585 | 1 | Phage protein |
| 1277124 | 1292950 | 1275784 | 1277127 | 1 | Replicative DNA helicase (DnaB) (EC 3.6.4.12) |
| 1277124 | 1292950 | 1277124 | 1277804 | 1 | hypothetical protein |
| 1277124 | 1292950 | 1278190 | 1278375 | 1 | hypothetical protein |
| 1277124 | 1292950 | 1278365 | 1278892 | 1 | FIG00957722: hypothetical protein |
| 1277124 | 1292950 | 1278889 | 1279146 | 1 | FIG00963725: hypothetical protein |
| 1277124 | 1292950 | 1279139 | 1279378 | 1 | FIG00954464: hypothetical protein |
| 1277124 | 1292950 | 1279371 | 1279604 | 1 | FIG034647: hypothetical protein in PFGI-1-like cluster |
| 1277124 | 1292950 | 1279604 | 1280626 | 1 | Nucleoid-associated protein NdpA |
| 1277124 | 1292950 | 1280623 | 1281513 | 1 | SAM-dependent methyltransferase |
| 1277124 | 1292950 | 1281534 | 1281803 | 1 | FIG00960798: hypothetical protein |
| 1277124 | 1292950 | 1281806 | 1283536 | 1 | Protein with ParB-like nuclease domain in PFGI-1-like cluster |
| 1277124 | 1292950 | 1283564 | 1284331 | 1 | FIG004780: hypothetical protein in PFGI-1-like cluster |
| 1277124 | 1292950 | 1284328 | 1285632 | 1 | FIG141751: hypothetical protein in PFGI-1-like cluster |
| 1277124 | 1292950 | 1285638 | 1286657 | -1 | hypothetical protein |
| 1277124 | 1292950 | 1286712 | 1286882 | 1 | FIG00960543: hypothetical protein |
| 1277124 | 1292950 | 1287039 | 1287767 | 1 | FIG141694: hypothetical protein in PFGI-1-like cluster |
| 1277124 | 1292950 | 1287773 | 1288321 | 1 | Integrase regulator R |
| 1277124 | 1292950 | 1288369 | 1289199 | 1 | hypothetical protein |
| 1277124 | 1292950 | 1289229 | 1289687 | 1 | Single-stranded DNA-binding protein |
| 1277124 | 1292950 | 1289793 | 1289987 | 1 | hypothetical protein |
| 1277124 | 1292950 | 1290515 | 1292434 | 1 | DNA topoisomerase I (EC 5.99.1.2) |
| 1277124 | 1292950 | 1292741 | 1292950 | 1 | Cold shock protein of CSP family |
| 1287696 | 1318768 | 1287039 | 1287767 | 1 | FIG141694: hypothetical protein in PFGI-1-like cluster |
| 1287696 | 1318768 | 1287773 | 1288321 | 1 | Integrase regulator R |
| 1287696 | 1318768 | 1288369 | 1289199 | 1 | hypothetical protein |
| 1287696 | 1318768 | 1289229 | 1289687 | 1 | Single-stranded DNA-binding protein |
| 1287696 | 1318768 | 1289793 | 1289987 | 1 | hypothetical protein |
| 1287696 | 1318768 | 1290515 | 1292434 | 1 | DNA topoisomerase I (EC 5.99.1.2) |
| 1287696 | 1318768 | 1292741 | 1292950 | 1 | Cold shock protein of CSP family |
| 1287696 | 1318768 | 1293172 | 1295061 | 1 | hypothetical protein |
| 1287696 | 1318768 | 1295058 | 1297034 | 1 | hypothetical protein |
| 1287696 | 1318768 | 1297044 | 1297178 | 1 | hypothetical protein |
| 1287696 | 1318768 | 1297590 | 1297880 | -1 | Addiction module antidote protein |
| 1287696 | 1318768 | 1297877 | 1297996 | -1 | Phage-related protein |
| 1287696 | 1318768 | 1298378 | 1299499 | 1 | hypothetical protein |
| 1287696 | 1318768 | 1299499 | 1301208 | 1 | Conjugative transfer protein PilN in PFGI-1-like cluster |
| 1287696 | 1318768 | 1301212 | 1302537 | 1 | hypothetical protein |
| 1287696 | 1318768 | 1302527 | 1303060 | 1 | Conjugative transfer protein PilP in PFGI-1-like cluster |
| 1287696 | 1318768 | 1303069 | 1304649 | 1 | IncI1 plasmid conjugative transfer ATPase PilQ |
| 1287696 | 1318768 | 1304649 | 1305728 | 1 | hypothetical protein |
| 1287696 | 1318768 | 1305750 | 1306280 | 1 | Conjugative transfer protein PilS in PFGI-1-like cluster |
| 1287696 | 1318768 | 1306277 | 1307233 | 1 | Conjugative transfer ATPase PilU in PFGI-1-like cluster |
| 1287696 | 1318768 | 1307226 | 1308605 | 1 | hypothetical protein |
| 1287696 | 1318768 | 1308623 | 1309060 | 1 | Conjugative transfer protein PilM in PFGI-1-like cluster |
| 1287696 | 1318768 | 1309982 | 1310548 | 1 | hypothetical protein |
| 1287696 | 1318768 | 1310806 | 1310940 | 1 | hypothetical protein |
| 1287696 | 1318768 | 1311263 | 1311751 | 1 | FIG051360: Periplasmic protein TonB, links inner and outer membranes |
| 1287696 | 1318768 | 1311889 | 1312047 | 1 | FIG00955915: hypothetical protein |
| 1287696 | 1318768 | 1312121 | 1312318 | 1 | hypothetical protein |
| 1287696 | 1318768 | 1312510 | 1313223 | 1 | hypothetical protein |
| 1287696 | 1318768 | 1313459 | 1313809 | 1 | FIG00960315: hypothetical protein |
| 1287696 | 1318768 | 1313866 | 1314498 | 1 | FIG034376: Hypothetical protein |
| 1287696 | 1318768 | 1314495 | 1314770 | 1 | FIG00957911: hypothetical protein |
| 1287696 | 1318768 | 1314840 | 1315202 | 1 | FIG046709: Hypothetical protein |
| 1287696 | 1318768 | 1315270 | 1315524 | 1 | FIG041301: Hypothetical protein |
| 1287696 | 1318768 | 1315617 | 1316222 | 1 | FIG026997: Hypothetical protein |
| 1287696 | 1318768 | 1316207 | 1316488 | -1 | FIG00960906: hypothetical protein |
| 1287696 | 1318768 | 1316510 | 1317697 | 1 | FIG023873: Plasmid related protein |
| 1287696 | 1318768 | 1317803 | 1320052 | 1 | Superfamily II DNA/RNA helicases, SNF2 family |
| 1323176 | 1331723 | 1323123 | 1323704 | 1 | Soluble lytic murein transglycosylase and related regulatory proteins (some contain LysM/invasin dom |
| 1323176 | 1331723 | 1323701 | 1324201 | 1 | hypothetical protein |
| 1323176 | 1331723 | 1324210 | 1324479 | 1 | COG1088: dTDP-D-glucose 4,6-dehydratase |
| 1323176 | 1331723 | 1324483 | 1326714 | 1 | Coupling protein VirD4, ATPase required for T-DNA transfer |
| 1323176 | 1331723 | 1326714 | 1327460 | 1 | putative membrane protein |
| 1323176 | 1331723 | 1328210 | 1329691 | 1 | Putative DNA helicase |
| 1323176 | 1331723 | 1329823 | 1330932 | 1 | hypothetical protein |
| 1323176 | 1331723 | 1331461 | 1331760 | 1 | Aconitase B |
| 1328210 | 1352420 | 1328210 | 1329691 | 1 | Putative DNA helicase |
| 1328210 | 1352420 | 1329823 | 1330932 | 1 | hypothetical protein |
| 1328210 | 1352420 | 1331461 | 1331760 | 1 | Aconitase B |
| 1328210 | 1352420 | 1331853 | 1332191 | 1 | Candidate type III effector Hop protein |
| 1328210 | 1352420 | 1332278 | 1332427 | 1 | conserved hypothetical protein |
| 1328210 | 1352420 | 1332580 | 1332801 | 1 | FIG00955871: hypothetical protein |
| 1328210 | 1352420 | 1332812 | 1333198 | 1 | FIG00953508: hypothetical protein |
| 1328210 | 1352420 | 1333195 | 1333854 | 1 | hypothetical protein |
| 1328210 | 1352420 | 1333851 | 1334735 | 1 | hypothetical protein |
| 1328210 | 1352420 | 1334719 | 1336224 | 1 | hypothetical protein |
| 1328210 | 1352420 | 1336202 | 1336645 | 1 | putative lipoprotein |
| 1328210 | 1352420 | 1336645 | 1339587 | 1 | Type IV secretory pathway, VirB4 components |
| 1328210 | 1352420 | 1339584 | 1339868 | 1 | hypothetical protein |
| 1328210 | 1352420 | 1339865 | 1340524 | 1 | Protein-disulfide isomerase |
| 1328210 | 1352420 | 1340521 | 1340739 | -1 | FIG00960085: hypothetical protein |
| 1328210 | 1352420 | 1341854 | 1342534 | 1 | hypothetical protein |
| 1328210 | 1352420 | 1342531 | 1343115 | 1 | Transcriptional regulator, TetR family |
| 1328210 | 1352420 | 1343175 | 1343951 | 1 | Oxidoreductase, short-chain dehydrogenase/reductase family |
| 1328210 | 1352420 | 1343966 | 1344532 | 1 | hypothetical protein |
| 1328210 | 1352420 | 1344732 | 1345268 | -1 | Transposase |
| 1328210 | 1352420 | 1345280 | 1345675 | -1 | FIG01213006: toxin |
| 1328210 | 1352420 | 1345672 | 1345923 | -1 | FIG01213332: antitoxin to FIG01213006: toxin |
| 1328210 | 1352420 | 1346105 | 1346671 | 1 | Phage DNA invertase |
| 1328210 | 1352420 | 1346753 | 1347682 | -1 | hypothetical protein |
| 1328210 | 1352420 | 1347682 | 1348830 | -1 | DNA-cytosine methyltransferase (EC 2.1.1.37) |
| 1328210 | 1352420 | 1349648 | 1349755 | -1 | hypothetical protein |
| 1328210 | 1352420 | 1349842 | 1350864 | -1 | Glycosyltransferase involved in cell wall biogenesis |
| 1328210 | 1352420 | 1350944 | 1351594 | -1 | hypothetical protein |
| 1328210 | 1352420 | 1351587 | 1352093 | -1 | hypothetical protein |
| 1328210 | 1352420 | 1352121 | 1352420 | -1 | hypothetical protein |
| 1328210 | 1352420 | 1352417 | 1353307 | -1 | probable DNA repair exonuclease |
| 1341683 | 1349733 | 1341854 | 1342534 | 1 | hypothetical protein |
| 1341683 | 1349733 | 1342531 | 1343115 | 1 | Transcriptional regulator, TetR family |
| 1341683 | 1349733 | 1343175 | 1343951 | 1 | Oxidoreductase, short-chain dehydrogenase/reductase family |
| 1341683 | 1349733 | 1343966 | 1344532 | 1 | hypothetical protein |
| 1341683 | 1349733 | 1344732 | 1345268 | -1 | Transposase |
| 1341683 | 1349733 | 1345280 | 1345675 | -1 | FIG01213006: toxin |
| 1341683 | 1349733 | 1345672 | 1345923 | -1 | FIG01213332: antitoxin to FIG01213006: toxin |
| 1341683 | 1349733 | 1346105 | 1346671 | 1 | Phage DNA invertase |
| 1341683 | 1349733 | 1346753 | 1347682 | -1 | hypothetical protein |
| 1341683 | 1349733 | 1347682 | 1348830 | -1 | DNA-cytosine methyltransferase (EC 2.1.1.37) |
| 1341683 | 1349733 | 1349648 | 1349755 | -1 | hypothetical protein |
| 1373484 | 1378286 | 1371934 | 1373649 | 1 | Efflux ABC transporter, permease/ATP-binding protein |
| 1373484 | 1378286 | 1373998 | 1378251 | 1 | Transposase |
| 1385624 | 1389667 | 1385624 | 1385917 | -1 | hypothetical protein |
| 1385624 | 1389667 | 1386052 | 1386729 | 1 | Thermostable hemolysin delta-VPH |
| 1385624 | 1389667 | 1386719 | 1388185 | 1 | Long-chain-fatty-acid--CoA ligase (EC 6.2.1.3) |
| 1385624 | 1389667 | 1388194 | 1388874 | 1 | Acyl-CoA long-chain enzyme transcriptional related heme family |
| 1385624 | 1389667 | 1388861 | 1389667 | 1 | Oxidoreductase, short-chain dehydrogenase/reductase family |
| 1385624 | 1389667 | 1389664 | 1390305 | 1 | hypothetical protein |
| 1406326 | 1412358 | 1406326 | 1407219 | 1 | Transcriptional regulator, MerR family |
| 1406326 | 1412358 | 1407414 | 1408784 | 1 | Mobile element protein |
| 1406326 | 1412358 | 1408926 | 1409045 | -1 | hypothetical protein |
| 1406326 | 1412358 | 1409159 | 1411081 | 1 | Pyruvate/2-oxoglutarate dehydrogenase complex, dihydrolipoamide acyltransferase (E2) component, and |
| 1406326 | 1412358 | 1411078 | 1412358 | 1 | Integrase |
| 1573989 | 1586729 | 1573989 | 1575293 | -1 | bacteriophage f237 ORF5; Probable coat protein A precursor |
| 1573989 | 1586729 | 1575429 | 1575677 | -1 | coat protein B of bacteriophage Pf1) |
| 1573989 | 1586729 | 1575690 | 1575941 | -1 | Phage protein |
| 1573989 | 1586729 | 1575954 | 1576046 | -1 | Phage protein |
| 1573989 | 1586729 | 1576062 | 1576496 | -1 | Helix destabilizing protein of bacteriophage Pf1 |
| 1573989 | 1586729 | 1577329 | 1577493 | -1 | hypothetical protein |
| 1573989 | 1586729 | 1577501 | 1577731 | -1 | 10.1 kDa protein (ORF 90) |
| 1573989 | 1586729 | 1578424 | 1578555 | 1 | hypothetical protein |
| 1573989 | 1586729 | 1578635 | 1579657 | -1 | Retron-type RNA-directed DNA polymerase (EC 2.7.7.49) |
| 1573989 | 1586729 | 1580279 | 1581124 | -1 | hypothetical protein |
| 1573989 | 1586729 | 1581560 | 1581865 | -1 | Antitoxin HigA |
| 1573989 | 1586729 | 1582484 | 1584712 | 1 | Aerobactin siderophore receptor IutA / Rhizobactin 1021 siderophore outer membrane receptor / Schizo |
| 1573989 | 1586729 | 1584782 | 1585429 | -1 | Carbonic anhydrase, beta class (EC 4.2.1.1) |
| 1573989 | 1586729 | 1585491 | 1586729 | -1 | hypothetical protein |
| 1576961 | 1581446 | 1577329 | 1577493 | -1 | hypothetical protein |
| 1576961 | 1581446 | 1577501 | 1577731 | -1 | 10.1 kDa protein (ORF 90) |
| 1576961 | 1581446 | 1578424 | 1578555 | 1 | hypothetical protein |
| 1576961 | 1581446 | 1578635 | 1579657 | -1 | Retron-type RNA-directed DNA polymerase (EC 2.7.7.49) |
| 1576961 | 1581446 | 1580279 | 1581124 | -1 | hypothetical protein |
| 2670294 | 2677822 | 2670294 | 2673557 | 1 | Putative large exoprotein involved in heme utilization or adhesion of ShlA/HecA/FhaA family |
| 2670294 | 2677822 | 2674102 | 2674803 | 1 | Transposase and inactivated derivatives |
| 2670294 | 2677822 | 2675023 | 2675163 | 1 | hypothetical protein |
| 2670294 | 2677822 | 2675649 | 2675804 | 1 | hypothetical protein |
| 2670294 | 2677822 | 2676372 | 2676566 | 1 | Large exoproteins involved in heme utilization or adhesion |
| 2670294 | 2677822 | 2676800 | 2676955 | -1 | hypothetical protein |
| 2670294 | 2677822 | 2677153 | 2677548 | 1 | hypothetical protein |
| 2670294 | 2677822 | 2677562 | 2677822 | -1 | FIG00954700: hypothetical protein |
| 2670294 | 2677822 | 2677819 | 2679072 | -1 | MgtC family |
| 3236962 | 3251621 | 3236625 | 3236972 | -1 | hypothetical protein |
| 3236962 | 3251621 | 3236962 | 3237576 | -1 | hypothetical protein |
| 3236962 | 3251621 | 3237767 | 3240493 | -1 | hypothetical protein |
| 3236962 | 3251621 | 3240623 | 3240781 | 1 | hypothetical protein |
| 3236962 | 3251621 | 3241171 | 3241308 | 1 | hypothetical protein |
| 3236962 | 3251621 | 3241888 | 3244428 | -1 | hypothetical protein |
| 3236962 | 3251621 | 3244425 | 3247082 | -1 | hypothetical protein |
| 3236962 | 3251621 | 3247149 | 3247919 | -1 | hypothetical protein |
| 3236962 | 3251621 | 3248091 | 3249212 | -1 | Fic domain protein, PA0574 type |
| 3236962 | 3251621 | 3249471 | 3249608 | -1 | Shufflon-specific DNA recombinase |
| 3236962 | 3251621 | 3249936 | 3251621 | -1 | Retron-type RNA-directed DNA polymerase (EC 2.7.7.49) |
| 3244425 | 3251621 | 3241888 | 3244428 | -1 | hypothetical protein |
| 3244425 | 3251621 | 3244425 | 3247082 | -1 | hypothetical protein |
| 3244425 | 3251621 | 3247149 | 3247919 | -1 | hypothetical protein |
| 3244425 | 3251621 | 3248091 | 3249212 | -1 | Fic domain protein, PA0574 type |
| 3244425 | 3251621 | 3249471 | 3249608 | -1 | Shufflon-specific DNA recombinase |
| 3244425 | 3251621 | 3249936 | 3251621 | -1 | Retron-type RNA-directed DNA polymerase (EC 2.7.7.49) |
| 3602645 | 3612745 | 3603081 | 3603224 | 1 | hypothetical protein |
| 3602645 | 3612745 | 3603234 | 3603362 | 1 | Hydrolases of the alpha/beta superfamily |
| 3602645 | 3612745 | 3604432 | 3605178 | -1 | hypothetical protein |
| 3602645 | 3612745 | 3605385 | 3606521 | 1 | hypothetical protein |
| 3602645 | 3612745 | 3606570 | 3606782 | 1 | Transcriptional regulator, AlpA like |
| 3602645 | 3612745 | 3607183 | 3608133 | 1 | hypothetical protein |
| 3602645 | 3612745 | 3608284 | 3610314 | -1 | hypothetical protein |
| 3602645 | 3612745 | 3610417 | 3610620 | 1 | hypothetical protein |
| 3602645 | 3612745 | 3610755 | 3611852 | -1 | hypothetical protein |
| 3602645 | 3612745 | 3611956 | 3613830 | -1 | DNA helicase related protein |
| 3627182 | 3642073 | 3629277 | 3631433 | 1 | hypothetical protein |
| 3627182 | 3642073 | 3631561 | 3634764 | -1 | hypothetical protein |
| 3627182 | 3642073 | 3634777 | 3636192 | -1 | hypothetical protein |
| 3627182 | 3642073 | 3636235 | 3637263 | -1 | transcriptional regulator-like protein |
| 3627182 | 3642073 | 3637439 | 3637558 | -1 | hypothetical protein |
| 3627182 | 3642073 | 3637635 | 3637847 | -1 | hypothetical protein |
| 3627182 | 3642073 | 3637903 | 3638796 | -1 | hypothetical protein |
| 3627182 | 3642073 | 3638796 | 3641357 | -1 | hypothetical protein |
| 3627182 | 3642073 | 3641356 | 3641487 | 1 | hypothetical protein |
| 3627182 | 3642073 | 3641554 | 3642000 | -1 | hypothetical protein |
| 3646925 | 3652191 | 3646925 | 3647197 | -1 | FIG00957676: hypothetical protein |
| 3646925 | 3652191 | 3647985 | 3649940 | -1 | hypothetical protein |
| 3646925 | 3652191 | 3649937 | 3650425 | -1 | hypothetical protein |
| 3646925 | 3652191 | 3650422 | 3651645 | -1 | hypothetical protein |
| 3646925 | 3652191 | 3652063 | 3652191 | -1 | hypothetical protein |
| 3647316 | 3652852 | 3647985 | 3649940 | -1 | hypothetical protein |
| 3647316 | 3652852 | 3649937 | 3650425 | -1 | hypothetical protein |
| 3647316 | 3652852 | 3650422 | 3651645 | -1 | hypothetical protein |
| 3647316 | 3652852 | 3652063 | 3652191 | -1 | hypothetical protein |
| 3647316 | 3652852 | 3652239 | 3653453 | -1 | Integrase |
| 3949127 | 3955770 | 3949127 | 3950803 | 1 | hypothetical protein |
| 3949127 | 3955770 | 3951045 | 3951173 | 1 | O-antigen acetylase |
| 3949127 | 3955770 | 3951443 | 3952405 | 1 | Dehydrogenases with different specificities (related to short-chain alcohol dehydrogenases) |
| 3949127 | 3955770 | 3952512 | 3953618 | 1 | Uncharacterized MFS-type transporter |
| 3949127 | 3955770 | 3953670 | 3954626 | -1 | Transcriptional regulator, AraC family |
| 3949127 | 3955770 | 3954775 | 3955770 | 1 | Putative oxidoreductase YncB |
| 5106917 | 5115465 | 5106525 | 5108000 | 1 | Type I restriction-modification system, DNA-methyltransferase subunit M (EC 2.1.1.72) |
| 5106917 | 5115465 | 5108636 | 5109067 | 1 | putative secreted protein |
| 5106917 | 5115465 | 5109224 | 5110237 | 1 | hypothetical protein |
| 5106917 | 5115465 | 5110237 | 5112435 | 1 | hypothetical protein |
| 5106917 | 5115465 | 5112647 | 5113243 | -1 | hypothetical protein |
| 5106917 | 5115465 | 5113357 | 5113482 | 1 | hypothetical protein |
| 5106917 | 5115465 | 5113528 | 5114439 | -1 | hypothetical protein |
| 5106917 | 5115465 | 5114449 | 5114811 | -1 | hypothetical protein |
| 5106917 | 5115465 | 5114808 | 5114963 | -1 | hypothetical protein |
| 5106917 | 5115465 | 5114960 | 5115187 | -1 | Transcriptional regulator in PFGI-1-like cluster |
| 5117154 | 5129609 | 5117154 | 5117729 | -1 | Transposase InsO for insertion sequence element IS911 |
| 5117154 | 5129609 | 5117756 | 5118064 | -1 | Transposase InsN for insertion sequence element IS911 |
| 5117154 | 5129609 | 5118125 | 5118295 | -1 | hypothetical protein |
| 5117154 | 5129609 | 5118460 | 5119809 | -1 | hypothetical protein |
| 5117154 | 5129609 | 5119806 | 5126102 | -1 | Superfamily I DNA and RNA helicases and helicase subunits |
| 5117154 | 5129609 | 5126117 | 5126755 | -1 | Ribonucleotide reductase of class III (anaerobic), activating protein (EC 1.97.1.4) |
| 5117154 | 5129609 | 5126757 | 5128586 | -1 | Chaperone protein ClpB (ATP-dependent unfoldase) |
| 5117154 | 5129609 | 5128717 | 5129256 | -1 | hypothetical protein |
| 5117154 | 5129609 | 5129331 | 5129609 | -1 | hypothetical protein |
| 5265458 | 5274027 | 5264590 | 5266332 | -1 | hypothetical protein |
| 5265458 | 5274027 | 5266336 | 5267094 | -1 | Cell division protein FtsK |
| 5265458 | 5274027 | 5267106 | 5267306 | -1 | hypothetical protein |
| 5265458 | 5274027 | 5267313 | 5268212 | -1 | Recombinational DNA repair protein RecT (prophage associated) |
| 5265458 | 5274027 | 5268225 | 5269133 | -1 | phage-related protein |
| 5265458 | 5274027 | 5269144 | 5269353 | -1 | hypothetical protein |
| 5265458 | 5274027 | 5269350 | 5269571 | -1 | FIG00955112: hypothetical protein |
| 5265458 | 5274027 | 5269555 | 5269701 | -1 | Phage protein |
| 5265458 | 5274027 | 5270181 | 5270552 | -1 | Carbon storage regulator |
| 5265458 | 5274027 | 5270588 | 5270800 | -1 | Phage protein |
| 5265458 | 5274027 | 5272759 | 5273424 | -1 | Phage repressor |
| 5267106 | 5270800 | 5267106 | 5267306 | -1 | hypothetical protein |
| 5267106 | 5270800 | 5267313 | 5268212 | -1 | Recombinational DNA repair protein RecT (prophage associated) |
| 5267106 | 5270800 | 5268225 | 5269133 | -1 | phage-related protein |
| 5267106 | 5270800 | 5269144 | 5269353 | -1 | hypothetical protein |
| 5267106 | 5270800 | 5269350 | 5269571 | -1 | FIG00955112: hypothetical protein |
| 5267106 | 5270800 | 5269555 | 5269701 | -1 | Phage protein |
| 5267106 | 5270800 | 5270181 | 5270552 | -1 | Carbon storage regulator |
| 5267106 | 5270800 | 5270588 | 5270800 | -1 | Phage protein |
| 5277941 | 5289274 | 5277692 | 5278234 | 1 | Phage terminase, small subunit |
| 5277941 | 5289274 | 5278218 | 5279471 | 1 | Phage terminase, large subunit |
| 5277941 | 5289274 | 5279471 | 5279668 | 1 | hypothetical protein |
| 5277941 | 5289274 | 5279671 | 5280990 | 1 | hypothetical protein |
| 5277941 | 5289274 | 5281004 | 5282734 | 1 | hypothetical protein |
| 5277941 | 5289274 | 5282738 | 5284015 | 1 | hypothetical protein |
| 5277941 | 5289274 | 5284019 | 5284468 | 1 | hypothetical protein |
| 5277941 | 5289274 | 5284484 | 5285578 | 1 | hypothetical protein |
| 5277941 | 5289274 | 5285589 | 5286179 | 1 | hypothetical protein |
| 5277941 | 5289274 | 5286264 | 5286665 | 1 | hypothetical protein |
| 5277941 | 5289274 | 5286662 | 5287000 | 1 | FIG01047296: hypothetical protein |
| 5277941 | 5289274 | 5286997 | 5287482 | -1 | Phage-associated homing endonuclease |
| 5277941 | 5289274 | 5287540 | 5287944 | 1 | hypothetical protein |
| 5277941 | 5289274 | 5287941 | 5288315 | 1 | hypothetical protein |
| 5277941 | 5289274 | 5288330 | 5289325 | 1 | hypothetical protein |
| 5286264 | 5304422 | 5286264 | 5286665 | 1 | hypothetical protein |
| 5286264 | 5304422 | 5286662 | 5287000 | 1 | FIG01047296: hypothetical protein |
| 5286264 | 5304422 | 5286997 | 5287482 | -1 | Phage-associated homing endonuclease |
| 5286264 | 5304422 | 5287540 | 5287944 | 1 | hypothetical protein |
| 5286264 | 5304422 | 5287941 | 5288315 | 1 | hypothetical protein |
| 5286264 | 5304422 | 5288330 | 5289325 | 1 | hypothetical protein |
| 5286264 | 5304422 | 5289322 | 5289939 | 1 | hypothetical protein |
| 5286264 | 5304422 | 5289939 | 5292425 | 1 | hypothetical protein |
| 5286264 | 5304422 | 5292422 | 5292889 | 1 | Phage protein |
| 5286264 | 5304422 | 5292951 | 5293364 | 1 | Phage protein |
| 5286264 | 5304422 | 5293369 | 5293776 | 1 | Phage peptidoglycan hydrolase (ACLAME 427) |
| 5286264 | 5304422 | 5293748 | 5296471 | 1 | Phage tail tip, host specificity protein J |
| 5286264 | 5304422 | 5296532 | 5298211 | 1 | Phage protein |
| 5286264 | 5304422 | 5298224 | 5300299 | 1 | hypothetical protein |
| 5286264 | 5304422 | 5300344 | 5300973 | 1 | pyocin R2_PP, lytic enzyme |
| 5286264 | 5304422 | 5300970 | 5301338 | 1 | Phage protein |
| 5286264 | 5304422 | 5301335 | 5301598 | 1 | Phage protein |
| 5286264 | 5304422 | 5301634 | 5301897 | 1 | Phage protein |
| 5286264 | 5304422 | 5301901 | 5302413 | -1 | Phage protein |
| 5286264 | 5304422 | 5302839 | 5304422 | -1 | Rhodanese-related sulfurtransferase, 4 domains |
| 5286264 | 5304422 | 5304419 | 5305024 | -1 | Cysteine dioxygenase (EC 1.13.11.20) |
| 5290458 | 5300359 | 5289939 | 5292425 | 1 | hypothetical protein |
| 5290458 | 5300359 | 5292422 | 5292889 | 1 | Phage protein |
| 5290458 | 5300359 | 5292951 | 5293364 | 1 | Phage protein |
| 5290458 | 5300359 | 5293369 | 5293776 | 1 | Phage peptidoglycan hydrolase (ACLAME 427) |
| 5290458 | 5300359 | 5293748 | 5296471 | 1 | Phage tail tip, host specificity protein J |
| 5290458 | 5300359 | 5296532 | 5298211 | 1 | Phage protein |
| 5290458 | 5300359 | 5298224 | 5300299 | 1 | hypothetical protein |
| 5290458 | 5300359 | 5300344 | 5300973 | 1 | pyocin R2_PP, lytic enzyme |
| 6161962 | 6173690 | 6161962 | 6163857 | 1 | hypothetical protein |
| 6161962 | 6173690 | 6164036 | 6165175 | 1 | Catalase-like heme-binding protein |
| 6161962 | 6173690 | 6165283 | 6165912 | -1 | hypothetical protein |
| 6161962 | 6173690 | 6165884 | 6166144 | -1 | Transposase |
| 6161962 | 6173690 | 6166421 | 6166723 | 1 | sulfatase |
| 6161962 | 6173690 | 6166737 | 6167219 | -1 | hypothetical protein |
| 6161962 | 6173690 | 6167312 | 6167776 | -1 | hypothetical protein |
| 6161962 | 6173690 | 6167773 | 6168201 | -1 | hypothetical protein |
| 6161962 | 6173690 | 6168194 | 6168754 | -1 | RNA polymerase ECF-type sigma factor |
| 6161962 | 6173690 | 6168905 | 6169429 | -1 | hypothetical protein |
| 6161962 | 6173690 | 6169439 | 6169846 | -1 | Ubiquinol-cytochrome C reductase, cytochrome B subunit (EC 1.10.2.2) |
| 6161962 | 6173690 | 6169986 | 6171581 | 1 | hypothetical protein |
| 6161962 | 6173690 | 6171952 | 6172713 | -1 | Mobile element protein |
| 6161962 | 6173690 | 6172821 | 6173690 | -1 | LysR-family transcriptional regulator PtxE, associated with phosphonate utilization |
| 6161962 | 6173690 | 6173684 | 6174694 | -1 | Phosphonate dehydrogenase (EC 1.20.1.1) |
| 6354604 | 6363720 | 6354604 | 6354825 | -1 | FIG00955622: hypothetical protein |
| 6354604 | 6363720 | 6355343 | 6355606 | -1 | Phage protein |
| 6354604 | 6363720 | 6355642 | 6355905 | -1 | Phage protein |
| 6354604 | 6363720 | 6356002 | 6356304 | 1 | hypothetical protein |
| 6354604 | 6363720 | 6356301 | 6356519 | 1 | Phage protein |
| 6354604 | 6363720 | 6356477 | 6356869 | -1 | Phage protein |
| 6354604 | 6363720 | 6356866 | 6357495 | -1 | pyocin R2_PP, lytic enzyme |
| 6354604 | 6363720 | 6357939 | 6359195 | -1 | hypothetical protein |
| 6354604 | 6363720 | 6359260 | 6360930 | -1 | Phage protein |
| 6354604 | 6363720 | 6360991 | 6363720 | -1 | Phage tail tip, host specificity protein J |
| 6354604 | 6363720 | 6363692 | 6364099 | -1 | Phage peptidoglycan hydrolase (ACLAME 427) |
| 6359260 | 6365058 | 6359260 | 6360930 | -1 | Phage protein |
| 6359260 | 6365058 | 6360991 | 6363720 | -1 | Phage tail tip, host specificity protein J |
| 6359260 | 6365058 | 6363692 | 6364099 | -1 | Phage peptidoglycan hydrolase (ACLAME 427) |
| 6359260 | 6365058 | 6364104 | 6364466 | -1 | Phage protein |
| 6359260 | 6365058 | 6364579 | 6365058 | -1 | Phage protein |
| 6359260 | 6365058 | 6365055 | 6367559 | -1 | Phage tail, tail length tape-measure protein H |
| 6371721 | 6383065 | 6371746 | 6372246 | -1 | hypothetical protein |
| 6371721 | 6383065 | 6372721 | 6373041 | -1 | FIG01061788: hypothetical protein |
| 6371721 | 6383065 | 6373022 | 6373282 | -1 | Orf7 |
| 6371721 | 6383065 | 6373785 | 6374972 | -1 | Phage major capsid protein |
| 6371721 | 6383065 | 6374969 | 6375859 | -1 | Phage head, head-tail preconnector protease C |
| 6371721 | 6383065 | 6375991 | 6376722 | -1 | Phage portal protein |
| 6371721 | 6383065 | 6377246 | 6377410 | -1 | FIG00963137: hypothetical protein |
| 6371721 | 6383065 | 6377407 | 6379098 | -1 | Phage terminase, large subunit |
| 6371721 | 6383065 | 6379100 | 6379324 | -1 | Orf1 |
| 6371721 | 6383065 | 6379693 | 6380007 | -1 | Phage-associated homing endonuclease |
| 6371721 | 6383065 | 6380007 | 6380168 | -1 | Phage protein |
| 6371721 | 6383065 | 6380161 | 6380286 | -1 | Phage protein |
| 6371721 | 6383065 | 6380286 | 6380537 | -1 | Phage protein |
| 6371721 | 6383065 | 6380537 | 6380821 | -1 | Phage holin |
| 6371721 | 6383065 | 6380814 | 6381218 | -1 | Phage holin; Phage membrane protein STY1040 |
| 6371721 | 6383065 | 6381957 | 6382643 | -1 | hypothetical protein |
| 6371721 | 6383065 | 6382714 | 6383031 | -1 | Phage protein |
| 6371721 | 6383065 | 6383031 | 6383672 | -1 | Phage recombination protein NinG |
| 6375991 | 6381218 | 6375991 | 6376722 | -1 | Phage portal protein |
| 6375991 | 6381218 | 6377246 | 6377410 | -1 | FIG00963137: hypothetical protein |
| 6375991 | 6381218 | 6377407 | 6379098 | -1 | Phage terminase, large subunit |
| 6375991 | 6381218 | 6379100 | 6379324 | -1 | Orf1 |
| 6375991 | 6381218 | 6379693 | 6380007 | -1 | Phage-associated homing endonuclease |
| 6375991 | 6381218 | 6380007 | 6380168 | -1 | Phage protein |
| 6375991 | 6381218 | 6380161 | 6380286 | -1 | Phage protein |
| 6375991 | 6381218 | 6380286 | 6380537 | -1 | Phage protein |
| 6375991 | 6381218 | 6380537 | 6380821 | -1 | Phage holin |
| 6375991 | 6381218 | 6380814 | 6381218 | -1 | Phage holin; Phage membrane protein STY1040 |
| 6395402 | 6411361 | 6395422 | 6396207 | 1 | hypothetical protein |
| 6395402 | 6411361 | 6396500 | 6396718 | 1 | hypothetical protein |
| 6395402 | 6411361 | 6397122 | 6397247 | 1 | hypothetical protein |
| 6395402 | 6411361 | 6397526 | 6397900 | 1 | Phage protein |
| 6395402 | 6411361 | 6397897 | 6398751 | 1 | Phage protein |
| 6395402 | 6411361 | 6398748 | 6399053 | 1 | Phage protein |
| 6395402 | 6411361 | 6399050 | 6399163 | 1 | hypothetical protein |
| 6395402 | 6411361 | 6399160 | 6399519 | 1 | hypothetical protein |
| 6395402 | 6411361 | 6399516 | 6399653 | 1 | Phage protein |
| 6395402 | 6411361 | 6399804 | 6400601 | 1 | Integrase |
| 6395402 | 6411361 | 6400598 | 6400924 | 1 | Phage protein |
| 6395402 | 6411361 | 6400967 | 6402031 | 1 | TolA protein |
| 6395402 | 6411361 | 6402172 | 6402792 | 1 | hypothetical protein |
| 6395402 | 6411361 | 6402796 | 6403422 | 1 | Phage exonuclease (EC 3.1.11.3); Putative phage-encoded enzyme involved in integration-recombination |
| 6395402 | 6411361 | 6403419 | 6403754 | 1 | Phage protein |
| 6395402 | 6411361 | 6403765 | 6403923 | 1 | Phage protein |
| 6395402 | 6411361 | 6403942 | 6404145 | 1 | Phage protein |
| 6395402 | 6411361 | 6404117 | 6404287 | 1 | Phage protein |
| 6395402 | 6411361 | 6404284 | 6405432 | 1 | Phage protein |
| 6395402 | 6411361 | 6405929 | 6406450 | 1 | Phage protein |
| 6395402 | 6411361 | 6406443 | 6406733 | 1 | Phage protein |
| 6395402 | 6411361 | 6406898 | 6407044 | 1 | Phage protein |
| 6395402 | 6411361 | 6407029 | 6407514 | 1 | Phage protein |
| 6395402 | 6411361 | 6407517 | 6407825 | 1 | Phage protein |
| 6395402 | 6411361 | 6408083 | 6408319 | 1 | hypothetical protein |
| 6395402 | 6411361 | 6408316 | 6408810 | 1 | Phage protein |
| 6395402 | 6411361 | 6408807 | 6409187 | 1 | hypothetical protein |
| 6395402 | 6411361 | 6409184 | 6409420 | 1 | Phage protein |
| 6395402 | 6411361 | 6409540 | 6409872 | 1 | Phage protein |
| 6395402 | 6411361 | 6410047 | 6410361 | 1 | hypothetical protein |
| 6395402 | 6411361 | 6411045 | 6411356 | 1 | Phage integrase |
| 6399160 | 6403422 | 6399050 | 6399163 | 1 | hypothetical protein |
| 6399160 | 6403422 | 6399160 | 6399519 | 1 | hypothetical protein |
| 6399160 | 6403422 | 6399516 | 6399653 | 1 | Phage protein |
| 6399160 | 6403422 | 6399804 | 6400601 | 1 | Integrase |
| 6399160 | 6403422 | 6400598 | 6400924 | 1 | Phage protein |
| 6399160 | 6403422 | 6400967 | 6402031 | 1 | TolA protein |
| 6399160 | 6403422 | 6402172 | 6402792 | 1 | hypothetical protein |
| 6399160 | 6403422 | 6402796 | 6403422 | 1 | Phage exonuclease (EC 3.1.11.3); Putative phage-encoded enzyme involved in integration-recombination |
| 6399160 | 6403422 | 6403419 | 6403754 | 1 | Phage protein |
| 6403942 | 6409420 | 6403942 | 6404145 | 1 | Phage protein |
| 6403942 | 6409420 | 6404117 | 6404287 | 1 | Phage protein |
| 6403942 | 6409420 | 6404284 | 6405432 | 1 | Phage protein |
| 6403942 | 6409420 | 6405929 | 6406450 | 1 | Phage protein |
| 6403942 | 6409420 | 6406443 | 6406733 | 1 | Phage protein |
| 6403942 | 6409420 | 6406898 | 6407044 | 1 | Phage protein |
| 6403942 | 6409420 | 6407029 | 6407514 | 1 | Phage protein |
| 6403942 | 6409420 | 6407517 | 6407825 | 1 | Phage protein |
| 6403942 | 6409420 | 6408083 | 6408319 | 1 | hypothetical protein |
| 6403942 | 6409420 | 6408316 | 6408810 | 1 | Phage protein |
| 6403942 | 6409420 | 6408807 | 6409187 | 1 | hypothetical protein |
| 6403942 | 6409420 | 6409184 | 6409420 | 1 | Phage protein |

**Table S2**. Predicted genomic islands on LYSZa2 genome by IslandViewer4 with at least one prediction method.
