# Supplementary material for: Pseudomonas aeruginosa modulates alginate biosynthesis and type VI secretion system in two critically ill COVID-19 patients
Source: Cell Biosci. 2022 Feb 9;12:14. doi: 10.1186/s13578-022-00748-z (PMC8827185; doi:10.1186/s13578-022-00748-z)
Supplement: Supplementary file 5 — Additional file 5: Table S3. Predicted genomic islands on LYSZa5 genome by IslandViewer4 with at least one prediction method. [file 13578_2022_748_MOESM5_ESM.docx]

| **Island start** | **Island end** | **Gene start** | **Gene end** | **Strand** | **Product** |
| --- | --- | --- | --- | --- | --- |
| 643006 | 651815 | 642875 | 644350 | 1 | Type I restriction-modification system, DNA-methyltransferase subunit M (EC 2.1.1.72) |
| 643006 | 651815 | 644986 | 645417 | 1 | putative secreted protein |
| 643006 | 651815 | 645574 | 646587 | 1 | hypothetical protein |
| 643006 | 651815 | 646587 | 648785 | 1 | hypothetical protein |
| 643006 | 651815 | 648997 | 649593 | -1 | hypothetical protein |
| 643006 | 651815 | 649707 | 649832 | 1 | hypothetical protein |
| 643006 | 651815 | 649878 | 650789 | -1 | hypothetical protein |
| 643006 | 651815 | 650799 | 651161 | -1 | hypothetical protein |
| 643006 | 651815 | 651158 | 651313 | -1 | hypothetical protein |
| 643006 | 651815 | 651310 | 651537 | -1 | Transcriptional regulator in PFGI-1-like cluster |
| 653504 | 665959 | 653504 | 654079 | -1 | Transposase InsO for insertion sequence element IS911 |
| 653504 | 665959 | 654106 | 654414 | -1 | Transposase InsN for insertion sequence element IS911 |
| 653504 | 665959 | 654475 | 654645 | -1 | hypothetical protein |
| 653504 | 665959 | 654810 | 656159 | -1 | hypothetical protein |
| 653504 | 665959 | 656156 | 662452 | -1 | Superfamily I DNA and RNA helicases and helicase subunits |
| 653504 | 665959 | 662467 | 663105 | -1 | Ribonucleotide reductase of class III (anaerobic), activating protein (EC 1.97.1.4) |
| 653504 | 665959 | 663107 | 664936 | -1 | Chaperone protein ClpB (ATP-dependent unfoldase) |
| 653504 | 665959 | 665067 | 665606 | -1 | hypothetical protein |
| 653504 | 665959 | 665681 | 665959 | -1 | hypothetical protein |
| 799154 | 810377 | 798821 | 799186 | -1 | C-5 cytosine-specific DNA methylase family protein |
| 799154 | 810377 | 799183 | 800037 | -1 | D12 class N6 adenine-specific DNA methyltransferase |
| 799154 | 810377 | 800177 | 800890 | -1 | Exodeoxyribonuclease X |
| 799154 | 810377 | 800940 | 802682 | -1 | hypothetical protein |
| 799154 | 810377 | 802686 | 803444 | -1 | Cell division protein FtsK |
| 799154 | 810377 | 803456 | 803656 | -1 | hypothetical protein |
| 799154 | 810377 | 803663 | 804562 | -1 | Recombinational DNA repair protein RecT (prophage associated) |
| 799154 | 810377 | 804575 | 805483 | -1 | phage-related protein |
| 799154 | 810377 | 805494 | 805703 | -1 | hypothetical protein |
| 799154 | 810377 | 805700 | 805921 | -1 | FIG00955112: hypothetical protein |
| 799154 | 810377 | 805905 | 806051 | -1 | Phage protein |
| 799154 | 810377 | 806531 | 806902 | -1 | Carbon storage regulator |
| 799154 | 810377 | 806938 | 807150 | -1 | Phage protein |
| 799154 | 810377 | 809109 | 809774 | -1 | Phage repressor |
| 803456 | 807150 | 803456 | 803656 | -1 | hypothetical protein |
| 803456 | 807150 | 803663 | 804562 | -1 | Recombinational DNA repair protein RecT (prophage associated) |
| 803456 | 807150 | 804575 | 805483 | -1 | phage-related protein |
| 803456 | 807150 | 805494 | 805703 | -1 | hypothetical protein |
| 803456 | 807150 | 805700 | 805921 | -1 | FIG00955112: hypothetical protein |
| 803456 | 807150 | 805905 | 806051 | -1 | Phage protein |
| 803456 | 807150 | 806531 | 806902 | -1 | Carbon storage regulator |
| 803456 | 807150 | 806938 | 807150 | -1 | Phage protein |
| 818011 | 836709 | 817354 | 819084 | 1 | hypothetical protein |
| 818011 | 836709 | 819088 | 820365 | 1 | hypothetical protein |
| 818011 | 836709 | 820369 | 820818 | 1 | hypothetical protein |
| 818011 | 836709 | 820834 | 821928 | 1 | hypothetical protein |
| 818011 | 836709 | 821939 | 822529 | 1 | hypothetical protein |
| 818011 | 836709 | 822614 | 823015 | 1 | hypothetical protein |
| 818011 | 836709 | 823012 | 823350 | 1 | FIG01047296: hypothetical protein |
| 818011 | 836709 | 823347 | 823832 | -1 | Phage-associated homing endonuclease |
| 818011 | 836709 | 823890 | 824294 | 1 | hypothetical protein |
| 818011 | 836709 | 824291 | 824665 | 1 | hypothetical protein |
| 818011 | 836709 | 824680 | 825675 | 1 | hypothetical protein |
| 818011 | 836709 | 825672 | 826289 | 1 | hypothetical protein |
| 818011 | 836709 | 826289 | 828775 | 1 | hypothetical protein |
| 818011 | 836709 | 828772 | 829239 | 1 | Phage protein |
| 818011 | 836709 | 829301 | 829714 | 1 | Phage protein |
| 818011 | 836709 | 829719 | 830126 | 1 | Phage peptidoglycan hydrolase (ACLAME 427) |
| 818011 | 836709 | 830098 | 832821 | 1 | Phage tail tip, host specificity protein J |
| 818011 | 836709 | 832882 | 834561 | 1 | Phage protein |
| 818011 | 836709 | 834574 | 836649 | 1 | hypothetical protein |
| 818011 | 836709 | 836694 | 837323 | 1 | pyocin R2_PP, lytic enzyme |
| 822614 | 840772 | 822614 | 823015 | 1 | hypothetical protein |
| 822614 | 840772 | 823012 | 823350 | 1 | FIG01047296: hypothetical protein |
| 822614 | 840772 | 823347 | 823832 | -1 | Phage-associated homing endonuclease |
| 822614 | 840772 | 823890 | 824294 | 1 | hypothetical protein |
| 822614 | 840772 | 824291 | 824665 | 1 | hypothetical protein |
| 822614 | 840772 | 824680 | 825675 | 1 | hypothetical protein |
| 822614 | 840772 | 825672 | 826289 | 1 | hypothetical protein |
| 822614 | 840772 | 826289 | 828775 | 1 | hypothetical protein |
| 822614 | 840772 | 828772 | 829239 | 1 | Phage protein |
| 822614 | 840772 | 829301 | 829714 | 1 | Phage protein |
| 822614 | 840772 | 829719 | 830126 | 1 | Phage peptidoglycan hydrolase (ACLAME 427) |
| 822614 | 840772 | 830098 | 832821 | 1 | Phage tail tip, host specificity protein J |
| 822614 | 840772 | 832882 | 834561 | 1 | Phage protein |
| 822614 | 840772 | 834574 | 836649 | 1 | hypothetical protein |
| 822614 | 840772 | 836694 | 837323 | 1 | pyocin R2_PP, lytic enzyme |
| 822614 | 840772 | 837320 | 837688 | 1 | Phage protein |
| 822614 | 840772 | 837685 | 837948 | 1 | Phage protein |
| 822614 | 840772 | 837984 | 838247 | 1 | Phage protein |
| 822614 | 840772 | 838251 | 838763 | -1 | Phage protein |
| 822614 | 840772 | 839189 | 840772 | -1 | Rhodanese-related sulfurtransferase, 4 domains |
| 822614 | 840772 | 840769 | 841374 | -1 | Cysteine dioxygenase (EC 1.13.11.20) |
| 1698312 | 1710040 | 1698312 | 1700207 | 1 | hypothetical protein |
| 1698312 | 1710040 | 1700386 | 1701525 | 1 | Catalase-like heme-binding protein |
| 1698312 | 1710040 | 1701633 | 1702262 | -1 | hypothetical protein |
| 1698312 | 1710040 | 1702234 | 1702494 | -1 | Transposase |
| 1698312 | 1710040 | 1702771 | 1703073 | 1 | sulfatase |
| 1698312 | 1710040 | 1703087 | 1703569 | -1 | hypothetical protein |
| 1698312 | 1710040 | 1703662 | 1704126 | -1 | hypothetical protein |
| 1698312 | 1710040 | 1704123 | 1704551 | -1 | hypothetical protein |
| 1698312 | 1710040 | 1704544 | 1705104 | -1 | RNA polymerase ECF-type sigma factor |
| 1698312 | 1710040 | 1705255 | 1705779 | -1 | hypothetical protein |
| 1698312 | 1710040 | 1705789 | 1706196 | -1 | Ubiquinol-cytochrome C reductase, cytochrome B subunit (EC 1.10.2.2) |
| 1698312 | 1710040 | 1706336 | 1707931 | 1 | hypothetical protein |
| 1698312 | 1710040 | 1708302 | 1709063 | -1 | Mobile element protein |
| 1698312 | 1710040 | 1709171 | 1710040 | -1 | LysR-family transcriptional regulator PtxE, associated with phosphonate utilization |
| 1698312 | 1710040 | 1710034 | 1711044 | -1 | Phosphonate dehydrogenase (EC 1.20.1.1) |
| 1890954 | 1900070 | 1890954 | 1891175 | -1 | FIG00955622: hypothetical protein |
| 1890954 | 1900070 | 1891693 | 1891956 | -1 | Phage protein |
| 1890954 | 1900070 | 1891992 | 1892255 | -1 | Phage protein |
| 1890954 | 1900070 | 1892352 | 1892654 | 1 | hypothetical protein |
| 1890954 | 1900070 | 1892651 | 1892869 | 1 | Phage protein |
| 1890954 | 1900070 | 1892827 | 1893219 | -1 | Phage protein |
| 1890954 | 1900070 | 1893216 | 1893845 | -1 | pyocin R2_PP, lytic enzyme |
| 1890954 | 1900070 | 1894289 | 1895545 | -1 | hypothetical protein |
| 1890954 | 1900070 | 1895610 | 1897280 | -1 | Phage protein |
| 1890954 | 1900070 | 1897341 | 1900070 | -1 | Phage tail tip, host specificity protein J |
| 1890954 | 1900070 | 1900042 | 1900449 | -1 | Phage peptidoglycan hydrolase (ACLAME 427) |
| 1895610 | 1901408 | 1895610 | 1897280 | -1 | Phage protein |
| 1895610 | 1901408 | 1897341 | 1900070 | -1 | Phage tail tip, host specificity protein J |
| 1895610 | 1901408 | 1900042 | 1900449 | -1 | Phage peptidoglycan hydrolase (ACLAME 427) |
| 1895610 | 1901408 | 1900454 | 1900816 | -1 | Phage protein |
| 1895610 | 1901408 | 1900929 | 1901408 | -1 | Phage protein |
| 1895610 | 1901408 | 1901405 | 1903909 | -1 | Phage tail, tail length tape-measure protein H |
| 1899824 | 1919415 | 1897341 | 1900070 | -1 | Phage tail tip, host specificity protein J |
| 1899824 | 1919415 | 1900042 | 1900449 | -1 | Phage peptidoglycan hydrolase (ACLAME 427) |
| 1899824 | 1919415 | 1900454 | 1900816 | -1 | Phage protein |
| 1899824 | 1919415 | 1900929 | 1901408 | -1 | Phage protein |
| 1899824 | 1919415 | 1901405 | 1903909 | -1 | Phage tail, tail length tape-measure protein H |
| 1899824 | 1919415 | 1903959 | 1904213 | -1 | Orf18 |
| 1899824 | 1919415 | 1904237 | 1904596 | -1 | tail protein |
| 1899824 | 1919415 | 1904606 | 1905127 | -1 | major tail protein, putative |
| 1899824 | 1919415 | 1905567 | 1906049 | -1 | Phage capsid and scaffold (ACLAME 58) |
| 1899824 | 1919415 | 1906042 | 1906659 | -1 | Phage protein |
| 1899824 | 1919415 | 1906656 | 1906841 | -1 | hypothetical protein |
| 1899824 | 1919415 | 1906838 | 1907671 | -1 | Phage protein |
| 1899824 | 1919415 | 1907675 | 1908034 | -1 | gene 66 protein |
| 1899824 | 1919415 | 1908096 | 1908596 | -1 | hypothetical protein |
| 1899824 | 1919415 | 1909071 | 1909391 | -1 | FIG01061788: hypothetical protein |
| 1899824 | 1919415 | 1909372 | 1909632 | -1 | Orf7 |
| 1899824 | 1919415 | 1910135 | 1911322 | -1 | Phage major capsid protein |
| 1899824 | 1919415 | 1911319 | 1912209 | -1 | Phage head, head-tail preconnector protease C |
| 1899824 | 1919415 | 1912341 | 1913072 | -1 | Phage portal protein |
| 1899824 | 1919415 | 1913596 | 1913760 | -1 | FIG00963137: hypothetical protein |
| 1899824 | 1919415 | 1913757 | 1915448 | -1 | Phage terminase, large subunit |
| 1899824 | 1919415 | 1915450 | 1915674 | -1 | Orf1 |
| 1899824 | 1919415 | 1916043 | 1916357 | -1 | Phage-associated homing endonuclease |
| 1899824 | 1919415 | 1916357 | 1916518 | -1 | Phage protein |
| 1899824 | 1919415 | 1916511 | 1916636 | -1 | Phage protein |
| 1899824 | 1919415 | 1916636 | 1916887 | -1 | Phage protein |
| 1899824 | 1919415 | 1916887 | 1917171 | -1 | Phage holin |
| 1899824 | 1919415 | 1917164 | 1917568 | -1 | Phage holin; Phage membrane protein STY1040 |
| 1899824 | 1919415 | 1918307 | 1918993 | -1 | hypothetical protein |
| 1899824 | 1919415 | 1919064 | 1919381 | -1 | Phage protein |
| 1899824 | 1919415 | 1919381 | 1920022 | -1 | Phage recombination protein NinG |
| 1912341 | 1917568 | 1912341 | 1913072 | -1 | Phage portal protein |
| 1912341 | 1917568 | 1913596 | 1913760 | -1 | FIG00963137: hypothetical protein |
| 1912341 | 1917568 | 1913757 | 1915448 | -1 | Phage terminase, large subunit |
| 1912341 | 1917568 | 1915450 | 1915674 | -1 | Orf1 |
| 1912341 | 1917568 | 1916043 | 1916357 | -1 | Phage-associated homing endonuclease |
| 1912341 | 1917568 | 1916357 | 1916518 | -1 | Phage protein |
| 1912341 | 1917568 | 1916511 | 1916636 | -1 | Phage protein |
| 1912341 | 1917568 | 1916636 | 1916887 | -1 | Phage protein |
| 1912341 | 1917568 | 1916887 | 1917171 | -1 | Phage holin |
| 1912341 | 1917568 | 1917164 | 1917568 | -1 | Phage holin; Phage membrane protein STY1040 |
| 1931752 | 1947711 | 1931772 | 1932557 | 1 | hypothetical protein |
| 1931752 | 1947711 | 1932850 | 1933068 | 1 | hypothetical protein |
| 1931752 | 1947711 | 1933472 | 1933597 | 1 | hypothetical protein |
| 1931752 | 1947711 | 1933876 | 1934250 | 1 | Phage protein |
| 1931752 | 1947711 | 1934247 | 1935101 | 1 | Phage protein |
| 1931752 | 1947711 | 1935098 | 1935403 | 1 | Phage protein |
| 1931752 | 1947711 | 1935400 | 1935513 | 1 | hypothetical protein |
| 1931752 | 1947711 | 1935510 | 1935869 | 1 | hypothetical protein |
| 1931752 | 1947711 | 1935866 | 1936003 | 1 | Phage protein |
| 1931752 | 1947711 | 1936154 | 1936951 | 1 | Integrase |
| 1931752 | 1947711 | 1936948 | 1937274 | 1 | Phage protein |
| 1931752 | 1947711 | 1937317 | 1938381 | 1 | TolA protein |
| 1931752 | 1947711 | 1938522 | 1939142 | 1 | hypothetical protein |
| 1931752 | 1947711 | 1939146 | 1939772 | 1 | Phage exonuclease (EC 3.1.11.3); Putative phage-encoded enzyme involved in integration-recombination |
| 1931752 | 1947711 | 1939769 | 1940104 | 1 | Phage protein |
| 1931752 | 1947711 | 1940115 | 1940273 | 1 | Phage protein |
| 1931752 | 1947711 | 1940292 | 1940495 | 1 | Phage protein |
| 1931752 | 1947711 | 1940467 | 1940637 | 1 | Phage protein |
| 1931752 | 1947711 | 1940634 | 1941782 | 1 | Phage protein |
| 1931752 | 1947711 | 1942279 | 1942800 | 1 | Phage protein |
| 1931752 | 1947711 | 1942793 | 1943083 | 1 | Phage protein |
| 1931752 | 1947711 | 1943248 | 1943394 | 1 | Phage protein |
| 1931752 | 1947711 | 1943379 | 1943864 | 1 | Phage protein |
| 1931752 | 1947711 | 1943867 | 1944175 | 1 | Phage protein |
| 1931752 | 1947711 | 1944433 | 1944669 | 1 | hypothetical protein |
| 1931752 | 1947711 | 1944666 | 1945160 | 1 | Phage protein |
| 1931752 | 1947711 | 1945157 | 1945537 | 1 | hypothetical protein |
| 1931752 | 1947711 | 1945534 | 1945770 | 1 | Phage protein |
| 1931752 | 1947711 | 1945890 | 1946222 | 1 | Phage protein |
| 1931752 | 1947711 | 1946397 | 1946711 | 1 | hypothetical protein |
| 1931752 | 1947711 | 1947395 | 1947706 | 1 | Phage integrase |
| 1935510 | 1939772 | 1935400 | 1935513 | 1 | hypothetical protein |
| 1935510 | 1939772 | 1935510 | 1935869 | 1 | hypothetical protein |
| 1935510 | 1939772 | 1935866 | 1936003 | 1 | Phage protein |
| 1935510 | 1939772 | 1936154 | 1936951 | 1 | Integrase |
| 1935510 | 1939772 | 1936948 | 1937274 | 1 | Phage protein |
| 1935510 | 1939772 | 1937317 | 1938381 | 1 | TolA protein |
| 1935510 | 1939772 | 1938522 | 1939142 | 1 | hypothetical protein |
| 1935510 | 1939772 | 1939146 | 1939772 | 1 | Phage exonuclease (EC 3.1.11.3); Putative phage-encoded enzyme involved in integration-recombination |
| 1935510 | 1939772 | 1939769 | 1940104 | 1 | Phage protein |
| 1940292 | 1945770 | 1940292 | 1940495 | 1 | Phage protein |
| 1940292 | 1945770 | 1940467 | 1940637 | 1 | Phage protein |
| 1940292 | 1945770 | 1940634 | 1941782 | 1 | Phage protein |
| 1940292 | 1945770 | 1942279 | 1942800 | 1 | Phage protein |
| 1940292 | 1945770 | 1942793 | 1943083 | 1 | Phage protein |
| 1940292 | 1945770 | 1943248 | 1943394 | 1 | Phage protein |
| 1940292 | 1945770 | 1943379 | 1943864 | 1 | Phage protein |
| 1940292 | 1945770 | 1943867 | 1944175 | 1 | Phage protein |
| 1940292 | 1945770 | 1944433 | 1944669 | 1 | hypothetical protein |
| 1940292 | 1945770 | 1944666 | 1945160 | 1 | Phage protein |
| 1940292 | 1945770 | 1945157 | 1945537 | 1 | hypothetical protein |
| 1940292 | 1945770 | 1945534 | 1945770 | 1 | Phage protein |
| 2612113 | 2632236 | 2612113 | 2615004 | 1 | Ribonucleotide reductase of class Ia (aerobic), alpha subunit (EC 1.17.4.1) |
| 2612113 | 2632236 | 2615267 | 2616514 | 1 | Ribonucleotide reductase of class Ia (aerobic), beta subunit (EC 1.17.4.1) |
| 2612113 | 2632236 | 2616949 | 2618904 | -1 | hypothetical protein |
| 2612113 | 2632236 | 2618904 | 2621300 | -1 | hypothetical protein |
| 2612113 | 2632236 | 2621317 | 2622144 | -1 | Uncharacterized protein MSMEG_1245 |
| 2612113 | 2632236 | 2622122 | 2623855 | -1 | hypothetical protein |
| 2612113 | 2632236 | 2624045 | 2625583 | -1 | hypothetical protein |
| 2612113 | 2632236 | 2625576 | 2626484 | -1 | hypothetical protein |
| 2612113 | 2632236 | 2628397 | 2629425 | -1 | hypothetical protein |
| 2612113 | 2632236 | 2629869 | 2631065 | -1 | Mobile element protein |
| 2612113 | 2632236 | 2631682 | 2632236 | 1 | Prophage antirepressor |
| 2784410 | 2801579 | 2783560 | 2784426 | -1 | MaoC-like dehydratase |
| 2784410 | 2801579 | 2784410 | 2786557 | -1 | Acetyl-CoA synthetase (ADP-forming) alpha and beta chains, putative |
| 2784410 | 2801579 | 2786667 | 2787818 | -1 | Sterol carrier protein IgrF |
| 2784410 | 2801579 | 2787815 | 2788126 | -1 | conserved protein associated with acetyl-CoA C-acyltransferase |
| 2784410 | 2801579 | 2788359 | 2789159 | 1 | Transcriptional regulator, IclR family |
| 2784410 | 2801579 | 2789199 | 2790098 | -1 | Glycosyl transferase, family 2 |
| 2784410 | 2801579 | 2790435 | 2792120 | 1 | hypothetical protein |
| 2784410 | 2801579 | 2792552 | 2793187 | 1 | hypothetical protein |
| 2784410 | 2801579 | 2793690 | 2793935 | -1 | hypothetical protein |
| 2784410 | 2801579 | 2794297 | 2795235 | 1 | 5'-nucleotidase (EC 3.1.3.5) |
| 2784410 | 2801579 | 2795239 | 2795973 | -1 | hypothetical protein |
| 2784410 | 2801579 | 2795966 | 2797159 | -1 | Retron-type RNA-directed DNA polymerase (EC 2.7.7.49) |
| 2784410 | 2801579 | 2798057 | 2798767 | -1 | Phosphoribosylaminoimidazole-succinocarboxamide synthase (EC 6.3.2.6) |
| 2784410 | 2801579 | 2798796 | 2799506 | -1 | Metal-dependent hydrolases of the beta-lactamase superfamily I |
| 2784410 | 2801579 | 2799493 | 2800683 | -1 | Outer membrane beta-barrel assembly protein BamC |
| 2784410 | 2801579 | 2800701 | 2801579 | -1 | 4-hydroxy-tetrahydrodipicolinate synthase (EC 4.3.3.7) |
| 2790559 | 2797872 | 2790435 | 2792120 | 1 | hypothetical protein |
| 2790559 | 2797872 | 2792552 | 2793187 | 1 | hypothetical protein |
| 2790559 | 2797872 | 2793690 | 2793935 | -1 | hypothetical protein |
| 2790559 | 2797872 | 2794297 | 2795235 | 1 | 5'-nucleotidase (EC 3.1.3.5) |
| 2790559 | 2797872 | 2795239 | 2795973 | -1 | hypothetical protein |
| 2790559 | 2797872 | 2795966 | 2797159 | -1 | Retron-type RNA-directed DNA polymerase (EC 2.7.7.49) |
| 3137673 | 3167157 | 3137131 | 3137676 | -1 | Extracytoplasmic function (ECF) sigma factor VreI |
| 3137673 | 3167157 | 3137673 | 3138389 | -1 | Outer membrane TonB-dependent transducer VreA of trans-envelope signaling system |
| 3137673 | 3167157 | 3138555 | 3138875 | 1 | hypothetical protein in cluster with VreARI signaling system |
| 3137673 | 3167157 | 3138993 | 3139589 | -1 | Heme oxygenase HemO, associated with heme uptake |
| 3137673 | 3167157 | 3139775 | 3140446 | 1 | RecA/RadA recombinase |
| 3137673 | 3167157 | 3140454 | 3141869 | 1 | DNA polymerase IV-like protein ImuB |
| 3137673 | 3167157 | 3141866 | 3144940 | 1 | Error-prone repair homolog of DNA polymerase III alpha subunit (EC 2.7.7.7) |
| 3137673 | 3167157 | 3151124 | 3152353 | 1 | Exonuclease SbcD |
| 3137673 | 3167157 | 3152362 | 3155997 | 1 | Exonuclease SbcC |
| 3137673 | 3167157 | 3156019 | 3158184 | -1 | Exodeoxyribonuclease V alpha chain (EC 3.1.11.5) |
| 3137673 | 3167157 | 3158181 | 3161918 | -1 | Exodeoxyribonuclease V beta chain (EC 3.1.11.5) |
| 3137673 | 3167157 | 3161915 | 3165430 | -1 | Exodeoxyribonuclease V gamma chain (EC 3.1.11.5) |
| 3137673 | 3167157 | 3165471 | 3166154 | -1 | Lipoate-protein ligase A |
| 3137673 | 3167157 | 3166267 | 3167157 | -1 | Permease of the drug/metabolite transporter (DMT) superfamily |
| 3445947 | 3455114 | 3446087 | 3446953 | 1 | ParA-like protein |
| 3445947 | 3455114 | 3446955 | 3447692 | 1 | Orf50 |
| 3445947 | 3455114 | 3447689 | 3448180 | 1 | hypothetical protein |
| 3445947 | 3455114 | 3448177 | 3448839 | 1 | hypothetical protein |
| 3445947 | 3455114 | 3448836 | 3449519 | 1 | hypothetical protein |
| 3445947 | 3455114 | 3449519 | 3450220 | 1 | FIG004780: hypothetical protein in PFGI-1-like cluster |
| 3445947 | 3455114 | 3450217 | 3450924 | 1 | Phage protein |
| 3445947 | 3455114 | 3450908 | 3451126 | 1 | FIG00953473: hypothetical protein |
| 3445947 | 3455114 | 3451123 | 3452466 | 1 | Replicative DNA helicase (DnaB) (EC 3.6.4.12) |
| 3445947 | 3455114 | 3452463 | 3453143 | 1 | hypothetical protein |
| 3445947 | 3455114 | 3453529 | 3453714 | 1 | hypothetical protein |
| 3445947 | 3455114 | 3453704 | 3454231 | 1 | FIG00957722: hypothetical protein |
| 3445947 | 3455114 | 3454228 | 3454485 | 1 | FIG00963725: hypothetical protein |
| 3445947 | 3455114 | 3454478 | 3454717 | 1 | FIG00954464: hypothetical protein |
| 3445947 | 3455114 | 3454710 | 3454943 | 1 | FIG034647: hypothetical protein in PFGI-1-like cluster |
| 3445947 | 3455114 | 3454943 | 3455965 | 1 | Nucleoid-associated protein NdpA |
| 3452463 | 3468289 | 3451123 | 3452466 | 1 | Replicative DNA helicase (DnaB) (EC 3.6.4.12) |
| 3452463 | 3468289 | 3452463 | 3453143 | 1 | hypothetical protein |
| 3452463 | 3468289 | 3453529 | 3453714 | 1 | hypothetical protein |
| 3452463 | 3468289 | 3453704 | 3454231 | 1 | FIG00957722: hypothetical protein |
| 3452463 | 3468289 | 3454228 | 3454485 | 1 | FIG00963725: hypothetical protein |
| 3452463 | 3468289 | 3454478 | 3454717 | 1 | FIG00954464: hypothetical protein |
| 3452463 | 3468289 | 3454710 | 3454943 | 1 | FIG034647: hypothetical protein in PFGI-1-like cluster |
| 3452463 | 3468289 | 3454943 | 3455965 | 1 | Nucleoid-associated protein NdpA |
| 3452463 | 3468289 | 3455962 | 3456852 | 1 | SAM-dependent methyltransferase |
| 3452463 | 3468289 | 3456873 | 3457142 | 1 | FIG00960798: hypothetical protein |
| 3452463 | 3468289 | 3457145 | 3458875 | 1 | Protein with ParB-like nuclease domain in PFGI-1-like cluster |
| 3452463 | 3468289 | 3458903 | 3459670 | 1 | FIG004780: hypothetical protein in PFGI-1-like cluster |
| 3452463 | 3468289 | 3459667 | 3460971 | 1 | FIG141751: hypothetical protein in PFGI-1-like cluster |
| 3452463 | 3468289 | 3460977 | 3461996 | -1 | hypothetical protein |
| 3452463 | 3468289 | 3462051 | 3462221 | 1 | FIG00960543: hypothetical protein |
| 3452463 | 3468289 | 3462378 | 3463106 | 1 | FIG141694: hypothetical protein in PFGI-1-like cluster |
| 3452463 | 3468289 | 3463112 | 3463660 | 1 | Integrase regulator R |
| 3452463 | 3468289 | 3463708 | 3464538 | 1 | hypothetical protein |
| 3452463 | 3468289 | 3464568 | 3465026 | 1 | Single-stranded DNA-binding protein |
| 3452463 | 3468289 | 3465132 | 3465326 | 1 | hypothetical protein |
| 3452463 | 3468289 | 3465854 | 3467773 | 1 | DNA topoisomerase I (EC 5.99.1.2) |
| 3452463 | 3468289 | 3468080 | 3468289 | 1 | Cold shock protein of CSP family |
| 3455885 | 3494107 | 3454943 | 3455965 | 1 | Nucleoid-associated protein NdpA |
| 3455885 | 3494107 | 3455962 | 3456852 | 1 | SAM-dependent methyltransferase |
| 3455885 | 3494107 | 3456873 | 3457142 | 1 | FIG00960798: hypothetical protein |
| 3455885 | 3494107 | 3457145 | 3458875 | 1 | Protein with ParB-like nuclease domain in PFGI-1-like cluster |
| 3455885 | 3494107 | 3458903 | 3459670 | 1 | FIG004780: hypothetical protein in PFGI-1-like cluster |
| 3455885 | 3494107 | 3459667 | 3460971 | 1 | FIG141751: hypothetical protein in PFGI-1-like cluster |
| 3455885 | 3494107 | 3460977 | 3461996 | -1 | hypothetical protein |
| 3455885 | 3494107 | 3462051 | 3462221 | 1 | FIG00960543: hypothetical protein |
| 3455885 | 3494107 | 3462378 | 3463106 | 1 | FIG141694: hypothetical protein in PFGI-1-like cluster |
| 3455885 | 3494107 | 3463112 | 3463660 | 1 | Integrase regulator R |
| 3455885 | 3494107 | 3463708 | 3464538 | 1 | hypothetical protein |
| 3455885 | 3494107 | 3464568 | 3465026 | 1 | Single-stranded DNA-binding protein |
| 3455885 | 3494107 | 3465132 | 3465326 | 1 | hypothetical protein |
| 3455885 | 3494107 | 3465854 | 3467773 | 1 | DNA topoisomerase I (EC 5.99.1.2) |
| 3455885 | 3494107 | 3468080 | 3468289 | 1 | Cold shock protein of CSP family |
| 3455885 | 3494107 | 3468511 | 3470400 | 1 | hypothetical protein |
| 3455885 | 3494107 | 3470397 | 3472373 | 1 | hypothetical protein |
| 3455885 | 3494107 | 3472383 | 3472517 | 1 | hypothetical protein |
| 3455885 | 3494107 | 3472929 | 3473219 | -1 | Addiction module antidote protein |
| 3455885 | 3494107 | 3473216 | 3473335 | -1 | Phage-related protein |
| 3455885 | 3494107 | 3473717 | 3474838 | 1 | hypothetical protein |
| 3455885 | 3494107 | 3474838 | 3476547 | 1 | Conjugative transfer protein PilN in PFGI-1-like cluster |
| 3455885 | 3494107 | 3476551 | 3477876 | 1 | hypothetical protein |
| 3455885 | 3494107 | 3477866 | 3478399 | 1 | Conjugative transfer protein PilP in PFGI-1-like cluster |
| 3455885 | 3494107 | 3478408 | 3479988 | 1 | IncI1 plasmid conjugative transfer ATPase PilQ |
| 3455885 | 3494107 | 3479988 | 3481067 | 1 | hypothetical protein |
| 3455885 | 3494107 | 3481089 | 3481619 | 1 | Conjugative transfer protein PilS in PFGI-1-like cluster |
| 3455885 | 3494107 | 3481616 | 3482572 | 1 | Conjugative transfer ATPase PilU in PFGI-1-like cluster |
| 3455885 | 3494107 | 3482565 | 3483944 | 1 | hypothetical protein |
| 3455885 | 3494107 | 3483962 | 3484399 | 1 | Conjugative transfer protein PilM in PFGI-1-like cluster |
| 3455885 | 3494107 | 3485321 | 3485887 | 1 | hypothetical protein |
| 3455885 | 3494107 | 3486145 | 3486279 | 1 | hypothetical protein |
| 3455885 | 3494107 | 3486602 | 3487090 | 1 | FIG051360: Periplasmic protein TonB, links inner and outer membranes |
| 3455885 | 3494107 | 3487228 | 3487386 | 1 | FIG00955915: hypothetical protein |
| 3455885 | 3494107 | 3487460 | 3487657 | 1 | hypothetical protein |
| 3455885 | 3494107 | 3487849 | 3488562 | 1 | hypothetical protein |
| 3455885 | 3494107 | 3488798 | 3489148 | 1 | FIG00960315: hypothetical protein |
| 3455885 | 3494107 | 3489205 | 3489837 | 1 | FIG034376: Hypothetical protein |
| 3455885 | 3494107 | 3489834 | 3490109 | 1 | FIG00957911: hypothetical protein |
| 3455885 | 3494107 | 3490179 | 3490541 | 1 | FIG046709: Hypothetical protein |
| 3455885 | 3494107 | 3490609 | 3490863 | 1 | FIG041301: Hypothetical protein |
| 3455885 | 3494107 | 3490956 | 3491561 | 1 | FIG026997: Hypothetical protein |
| 3455885 | 3494107 | 3491546 | 3491827 | -1 | FIG00960906: hypothetical protein |
| 3455885 | 3494107 | 3491849 | 3493036 | 1 | FIG023873: Plasmid related protein |
| 3455885 | 3494107 | 3493142 | 3495391 | 1 | Superfamily II DNA/RNA helicases, SNF2 family |
| 3498515 | 3507062 | 3498462 | 3499043 | 1 | Soluble lytic murein transglycosylase and related regulatory proteins (some contain LysM/invasin dom |
| 3498515 | 3507062 | 3499040 | 3499540 | 1 | hypothetical protein |
| 3498515 | 3507062 | 3499549 | 3499818 | 1 | COG1088: dTDP-D-glucose 4,6-dehydratase |
| 3498515 | 3507062 | 3499822 | 3502053 | 1 | Coupling protein VirD4, ATPase required for T-DNA transfer |
| 3498515 | 3507062 | 3502053 | 3502799 | 1 | putative membrane protein |
| 3498515 | 3507062 | 3503549 | 3505030 | 1 | Putative DNA helicase |
| 3498515 | 3507062 | 3505162 | 3506271 | 1 | hypothetical protein |
| 3498515 | 3507062 | 3506800 | 3507099 | 1 | Aconitase B |
| 3503549 | 3527759 | 3503549 | 3505030 | 1 | Putative DNA helicase |
| 3503549 | 3527759 | 3505162 | 3506271 | 1 | hypothetical protein |
| 3503549 | 3527759 | 3506800 | 3507099 | 1 | Aconitase B |
| 3503549 | 3527759 | 3507192 | 3507530 | 1 | Candidate type III effector Hop protein |
| 3503549 | 3527759 | 3507617 | 3507766 | 1 | conserved hypothetical protein |
| 3503549 | 3527759 | 3507919 | 3508140 | 1 | FIG00955871: hypothetical protein |
| 3503549 | 3527759 | 3508151 | 3508537 | 1 | FIG00953508: hypothetical protein |
| 3503549 | 3527759 | 3508534 | 3509193 | 1 | hypothetical protein |
| 3503549 | 3527759 | 3509190 | 3510074 | 1 | hypothetical protein |
| 3503549 | 3527759 | 3510058 | 3511563 | 1 | hypothetical protein |
| 3503549 | 3527759 | 3511541 | 3511984 | 1 | putative lipoprotein |
| 3503549 | 3527759 | 3511984 | 3514926 | 1 | Type IV secretory pathway, VirB4 components |
| 3503549 | 3527759 | 3514923 | 3515207 | 1 | hypothetical protein |
| 3503549 | 3527759 | 3515204 | 3515863 | 1 | Protein-disulfide isomerase |
| 3503549 | 3527759 | 3515860 | 3516078 | -1 | FIG00960085: hypothetical protein |
| 3503549 | 3527759 | 3517193 | 3517873 | 1 | hypothetical protein |
| 3503549 | 3527759 | 3517870 | 3518454 | 1 | Transcriptional regulator, TetR family |
| 3503549 | 3527759 | 3518514 | 3519290 | 1 | Oxidoreductase, short-chain dehydrogenase/reductase family |
| 3503549 | 3527759 | 3519305 | 3519871 | 1 | hypothetical protein |
| 3503549 | 3527759 | 3520071 | 3520607 | -1 | Transposase |
| 3503549 | 3527759 | 3520619 | 3521014 | -1 | FIG01213006: toxin |
| 3503549 | 3527759 | 3521011 | 3521262 | -1 | FIG01213332: antitoxin to FIG01213006: toxin |
| 3503549 | 3527759 | 3521444 | 3522010 | 1 | Phage DNA invertase |
| 3503549 | 3527759 | 3522092 | 3523021 | -1 | hypothetical protein |
| 3503549 | 3527759 | 3523021 | 3524169 | -1 | DNA-cytosine methyltransferase (EC 2.1.1.37) |
| 3503549 | 3527759 | 3524987 | 3525094 | -1 | hypothetical protein |
| 3503549 | 3527759 | 3525181 | 3526203 | -1 | Glycosyltransferase involved in cell wall biogenesis |
| 3503549 | 3527759 | 3526283 | 3526933 | -1 | hypothetical protein |
| 3503549 | 3527759 | 3526926 | 3527432 | -1 | hypothetical protein |
| 3503549 | 3527759 | 3527460 | 3527759 | -1 | hypothetical protein |
| 3503549 | 3527759 | 3527756 | 3528646 | -1 | probable DNA repair exonuclease |
| 3517022 | 3525062 | 3517193 | 3517873 | 1 | hypothetical protein |
| 3517022 | 3525062 | 3517870 | 3518454 | 1 | Transcriptional regulator, TetR family |
| 3517022 | 3525062 | 3518514 | 3519290 | 1 | Oxidoreductase, short-chain dehydrogenase/reductase family |
| 3517022 | 3525062 | 3519305 | 3519871 | 1 | hypothetical protein |
| 3517022 | 3525062 | 3520071 | 3520607 | -1 | Transposase |
| 3517022 | 3525062 | 3520619 | 3521014 | -1 | FIG01213006: toxin |
| 3517022 | 3525062 | 3521011 | 3521262 | -1 | FIG01213332: antitoxin to FIG01213006: toxin |
| 3517022 | 3525062 | 3521444 | 3522010 | 1 | Phage DNA invertase |
| 3517022 | 3525062 | 3522092 | 3523021 | -1 | hypothetical protein |
| 3517022 | 3525062 | 3523021 | 3524169 | -1 | DNA-cytosine methyltransferase (EC 2.1.1.37) |
| 3517022 | 3525062 | 3524987 | 3525094 | -1 | hypothetical protein |
| 3548823 | 3553625 | 3547273 | 3548988 | 1 | Efflux ABC transporter, permease/ATP-binding protein |
| 3548823 | 3553625 | 3549337 | 3553590 | 1 | Transposase |
| 3560963 | 3565006 | 3560963 | 3561256 | -1 | hypothetical protein |
| 3560963 | 3565006 | 3561391 | 3562068 | 1 | Thermostable hemolysin delta-VPH |
| 3560963 | 3565006 | 3562058 | 3563524 | 1 | Long-chain-fatty-acid--CoA ligase (EC 6.2.1.3) |
| 3560963 | 3565006 | 3563533 | 3564213 | 1 | Acyl-CoA long-chain enzyme transcriptional related heme family |
| 3560963 | 3565006 | 3564200 | 3565006 | 1 | Oxidoreductase, short-chain dehydrogenase/reductase family |
| 3560963 | 3565006 | 3565003 | 3565644 | 1 | hypothetical protein |
| 3581665 | 3587697 | 3581665 | 3582558 | 1 | Transcriptional regulator, MerR family |
| 3581665 | 3587697 | 3582753 | 3584123 | 1 | Mobile element protein |
| 3581665 | 3587697 | 3584265 | 3584384 | -1 | hypothetical protein |
| 3581665 | 3587697 | 3584498 | 3586420 | 1 | Pyruvate/2-oxoglutarate dehydrogenase complex, dihydrolipoamide acyltransferase (E2) component, and |
| 3581665 | 3587697 | 3586417 | 3587697 | 1 | Integrase |
| 3749328 | 3762068 | 3749328 | 3750632 | -1 | bacteriophage f237 ORF5; Probable coat protein A precursor |
| 3749328 | 3762068 | 3750768 | 3751016 | -1 | coat protein B of bacteriophage Pf1) |
| 3749328 | 3762068 | 3751029 | 3751280 | -1 | Phage protein |
| 3749328 | 3762068 | 3751293 | 3751385 | -1 | Phage protein |
| 3749328 | 3762068 | 3751401 | 3751835 | -1 | Helix destabilizing protein of bacteriophage Pf1 |
| 3749328 | 3762068 | 3752668 | 3752832 | -1 | hypothetical protein |
| 3749328 | 3762068 | 3752840 | 3753070 | -1 | 10.1 kDa protein (ORF 90) |
| 3749328 | 3762068 | 3753763 | 3753894 | 1 | hypothetical protein |
| 3749328 | 3762068 | 3753974 | 3754996 | -1 | Retron-type RNA-directed DNA polymerase (EC 2.7.7.49) |
| 3749328 | 3762068 | 3755618 | 3756463 | -1 | hypothetical protein |
| 3749328 | 3762068 | 3756899 | 3757204 | -1 | Antitoxin HigA |
| 3749328 | 3762068 | 3757823 | 3760051 | 1 | Aerobactin siderophore receptor IutA / Rhizobactin 1021 siderophore outer membrane receptor / Schizo |
| 3749328 | 3762068 | 3760121 | 3760768 | -1 | Carbonic anhydrase, beta class (EC 4.2.1.1) |
| 3749328 | 3762068 | 3760830 | 3762068 | -1 | hypothetical protein |
| 3752300 | 3756785 | 3752668 | 3752832 | -1 | hypothetical protein |
| 3752300 | 3756785 | 3752840 | 3753070 | -1 | 10.1 kDa protein (ORF 90) |
| 3752300 | 3756785 | 3753763 | 3753894 | 1 | hypothetical protein |
| 3752300 | 3756785 | 3753974 | 3754996 | -1 | Retron-type RNA-directed DNA polymerase (EC 2.7.7.49) |
| 3752300 | 3756785 | 3755618 | 3756463 | -1 | hypothetical protein |
| 4845634 | 4853162 | 4845634 | 4848897 | 1 | Putative large exoprotein involved in heme utilization or adhesion of ShlA/HecA/FhaA family |
| 4845634 | 4853162 | 4849442 | 4850143 | 1 | Transposase and inactivated derivatives |
| 4845634 | 4853162 | 4850363 | 4850503 | 1 | hypothetical protein |
| 4845634 | 4853162 | 4850989 | 4851144 | 1 | hypothetical protein |
| 4845634 | 4853162 | 4851712 | 4851906 | 1 | Large exoproteins involved in heme utilization or adhesion |
| 4845634 | 4853162 | 4852140 | 4852295 | -1 | hypothetical protein |
| 4845634 | 4853162 | 4852493 | 4852888 | 1 | hypothetical protein |
| 4845634 | 4853162 | 4852902 | 4853162 | -1 | FIG00954700: hypothetical protein |
| 4845634 | 4853162 | 4853159 | 4854412 | -1 | MgtC family |
| 5412303 | 5426962 | 5411966 | 5412313 | -1 | hypothetical protein |
| 5412303 | 5426962 | 5412303 | 5412917 | -1 | hypothetical protein |
| 5412303 | 5426962 | 5413108 | 5415834 | -1 | hypothetical protein |
| 5412303 | 5426962 | 5415964 | 5416122 | 1 | hypothetical protein |
| 5412303 | 5426962 | 5416512 | 5416649 | 1 | hypothetical protein |
| 5412303 | 5426962 | 5417229 | 5419769 | -1 | hypothetical protein |
| 5412303 | 5426962 | 5419766 | 5422423 | -1 | hypothetical protein |
| 5412303 | 5426962 | 5422490 | 5423260 | -1 | hypothetical protein |
| 5412303 | 5426962 | 5423432 | 5424553 | -1 | Fic domain protein, PA0574 type |
| 5412303 | 5426962 | 5424812 | 5424949 | -1 | Shufflon-specific DNA recombinase |
| 5412303 | 5426962 | 5425277 | 5426962 | -1 | Retron-type RNA-directed DNA polymerase (EC 2.7.7.49) |
| 5419766 | 5426962 | 5417229 | 5419769 | -1 | hypothetical protein |
| 5419766 | 5426962 | 5419766 | 5422423 | -1 | hypothetical protein |
| 5419766 | 5426962 | 5422490 | 5423260 | -1 | hypothetical protein |
| 5419766 | 5426962 | 5423432 | 5424553 | -1 | Fic domain protein, PA0574 type |
| 5419766 | 5426962 | 5424812 | 5424949 | -1 | Shufflon-specific DNA recombinase |
| 5419766 | 5426962 | 5425277 | 5426962 | -1 | Retron-type RNA-directed DNA polymerase (EC 2.7.7.49) |
| 5777986 | 5788086 | 5778422 | 5778565 | 1 | hypothetical protein |
| 5777986 | 5788086 | 5778575 | 5778703 | 1 | Hydrolases of the alpha/beta superfamily |
| 5777986 | 5788086 | 5779773 | 5780519 | -1 | hypothetical protein |
| 5777986 | 5788086 | 5780726 | 5781862 | 1 | hypothetical protein |
| 5777986 | 5788086 | 5781911 | 5782123 | 1 | Transcriptional regulator, AlpA like |
| 5777986 | 5788086 | 5782524 | 5783474 | 1 | hypothetical protein |
| 5777986 | 5788086 | 5783625 | 5785655 | -1 | hypothetical protein |
| 5777986 | 5788086 | 5785758 | 5785961 | 1 | hypothetical protein |
| 5777986 | 5788086 | 5786096 | 5787193 | -1 | hypothetical protein |
| 5777986 | 5788086 | 5787297 | 5789171 | -1 | DNA helicase related protein |
| 5802523 | 5817414 | 5804618 | 5806774 | 1 | hypothetical protein |
| 5802523 | 5817414 | 5806902 | 5810105 | -1 | hypothetical protein |
| 5802523 | 5817414 | 5810118 | 5811533 | -1 | hypothetical protein |
| 5802523 | 5817414 | 5811576 | 5812604 | -1 | transcriptional regulator-like protein |
| 5802523 | 5817414 | 5812780 | 5812899 | -1 | hypothetical protein |
| 5802523 | 5817414 | 5812976 | 5813188 | -1 | hypothetical protein |
| 5802523 | 5817414 | 5813244 | 5814137 | -1 | hypothetical protein |
| 5802523 | 5817414 | 5814137 | 5816698 | -1 | hypothetical protein |
| 5802523 | 5817414 | 5816697 | 5816828 | 1 | hypothetical protein |
| 5802523 | 5817414 | 5816895 | 5817341 | -1 | hypothetical protein |
| 5822266 | 5827532 | 5822266 | 5822538 | -1 | FIG00957676: hypothetical protein |
| 5822266 | 5827532 | 5823326 | 5825281 | -1 | hypothetical protein |
| 5822266 | 5827532 | 5825278 | 5825766 | -1 | hypothetical protein |
| 5822266 | 5827532 | 5825763 | 5826986 | -1 | hypothetical protein |
| 5822266 | 5827532 | 5827404 | 5827532 | -1 | hypothetical protein |
| 5822657 | 5828193 | 5823326 | 5825281 | -1 | hypothetical protein |
| 5822657 | 5828193 | 5825278 | 5825766 | -1 | hypothetical protein |
| 5822657 | 5828193 | 5825763 | 5826986 | -1 | hypothetical protein |
| 5822657 | 5828193 | 5827404 | 5827532 | -1 | hypothetical protein |
| 5822657 | 5828193 | 5827580 | 5828794 | -1 | Integrase |
| 6124467 | 6131110 | 6124467 | 6126143 | 1 | hypothetical protein |
| 6124467 | 6131110 | 6126385 | 6126513 | 1 | O-antigen acetylase |
| 6124467 | 6131110 | 6126783 | 6127745 | 1 | Dehydrogenases with different specificities (related to short-chain alcohol dehydrogenases) |
| 6124467 | 6131110 | 6127852 | 6128958 | 1 | Uncharacterized MFS-type transporter |
| 6124467 | 6131110 | 6129010 | 6129966 | -1 | Transcriptional regulator, AraC family |
| 6124467 | 6131110 | 6130115 | 6131110 | 1 | Putative oxidoreductase YncB |

**Table S3**. Predicted genomic islands on LYSZa5 genome by IslandViewer4 with at least one prediction method.
