# Supplementary material for: Pseudomonas aeruginosa modulates alginate biosynthesis and type VI secretion system in two critically ill COVID-19 patients
Source: Cell Biosci. 2022 Feb 9;12:14. doi: 10.1186/s13578-022-00748-z (PMC8827185; doi:10.1186/s13578-022-00748-z)
Supplement: Supplementary file 6 — Additional file 6: Table S4. Genomic accession numbers of P. aeruginosa strains selected for phylogenetic tree construction. [file 13578_2022_748_MOESM6_ESM.docx]

| **Strain** | **NCBI/GenBank Accession** |
| --- | --- |
| *Pseudomonas aeruginosa LYSZa7* | CP061699.1 |
| *Pseudomonas aeruginosa* DK2 | NC_018080.1 |
| *Pseudomonas aeruginosa* strain F30658 | NZ_CP008857.1 |
| *Pseudomonas aeruginosa strain* Cu1510 | CP013144.1 |
| *Pseudomonas aeruginosa* strain LES431 | NC_023066.1 |
| *Pseudomonas aeruginosa* M18 | NC_017548.1 |
| *Pseudomonas aeruginosa* strain M1608 | NZ_CP008862.2 |
| *Pseudomonas aeruginosa* MTB-1 | NC_023019.1 |
| *Pseudomonas aeruginosa* strain N17-1 | NZ_CP014948.1 |
| *Pseudomonas aeruginosa* PA1 | NC_022808.2 |
| *Pseudomonas aeruginosa* RP73 | NC_021577.1 |
| *Pseudomonas aeruginosa* SCV20265 | NC_023149.1 |
| *Pseudomonas aeruginosa* SJTD-1 | NZ_CP015877.1 |
| *Pseudomonas aeruginosa* strain T63266 | NZ_CP008868.1 |
| *Pseudomonas aeruginosa* UCBPP-PA14 | NC_008463.1 |
| *Pseudomonas aeruginosa* YL84 | NZ_CP007147.1 |
| *Pseudomonas aeruginosa* strain Pa1207 | NZ_CP022001 |
| *Pseudomonas aeruginosa* SP4528 | NZ_CP033439.1 |
| *Pseudomonas aeruginosa* PAK | LR657304.1 |
| *Pseudomonas aeruginosa* strain LESB58 | NC_011770.1 |
| *Pseudomonas aeruginosa* strain PA1 | NZ_NIZN00000000.1 |
| *Pseudomonas aeruginosa* strain PA34 | NZ_CP032552 |
| *Pseudomonas aeruginosa* PAO1 | NC_002516.2 |

**Table S4.** Genomic accession numbers of *P. aeruginosa* strains selected for phylogenetic tree construction.
