# Supplementary material for: Pseudomonas aeruginosa modulates alginate biosynthesis and type VI secretion system in two critically ill COVID-19 patients
Source: Cell Biosci. 2022 Feb 9;12:14. doi: 10.1186/s13578-022-00748-z (PMC8827185; doi:10.1186/s13578-022-00748-z)
Supplement: Supplementary file 7 — Additional file 7: Table S5. Single nucleotide polymorphism (SNP) and other genome modifying events identified between the ancestry isolates and the progeny isolates using PAO1 as reference. SNV Single nucleotide variation, MNV Multi-nucleotide variation. [file 13578_2022_748_MOESM7_ESM.docx]

| Gene | Product | LYSZa3 vs LYSZa2 | | | | LYSZa6 vs LYSZa5 | | | |  |
| --- | --- | --- | --- | --- | --- | --- | --- | --- | --- | --- |
|  |  | Type | Amino acid change | Coding region change in longest transcript | Non-synonymous | Type | Amino acid change | Coding region change in longest transcript | Non-synonymous |  |
| PA0041 | hemagglutinin | SNV | - | 5803C>T | No | SNV | - | 5803C>T | No |  |
| PA0259 | Type 6 lipase adaptor, Tla3 | SNV | - | 333A>G | No | SNV | - | 333A>G | No |  |
| PA0259 | Type 6 lipase adaptor, Tla3 | MNV | - | 330_331delinsTT | No | MNV | - | 330_331delinsTT | No |  |
| PA0259 | Type 6 lipase adaptor, Tla3 | SNV | - | 327T>G | No | SNV | - | 327T>G | No |  |
| PA0259 | Type 6 lipase adaptor, Tla3 | SNV | - | 324T>A | No | SNV | - | 324T>A | No |  |
| PA0259 | Type 6 lipase adaptor, Tla3 | MNV | Ser107Asn | 320_321delinsAT | Yes | MNV | Ser107Asn | 320_321delinsAT | Yes |  |
| PA0259 | Type 6 lipase adaptor, Tla3 | SNV | - | 315T>C | No | SNV | - | 315T>C | No |  |
| PA0259 | Type 6 lipase adaptor, Tla3 | SNV | Asp105Asn | 313G>A | Yes | SNV | Asp105Asn | 313G>A | Yes |  |
| PA0259 | Type 6 lipase adaptor, Tla3 | SNV | - | 303G>A | No | SNV | - | 303G>A | No |  |
| PA0262 | VgrG2b (HSI-II) | - | - | - | - | SNV | - | 480T>C | No |  |
| mexR | multidrug resistance operon repressor MexR | - | - | - | - | SNV | Cys138Trp | 414C>G | Yes |  |
| mexR | multidrug resistance operon repressor MexR | - | - | - | - | SNV | Cys138Gly | 412T>G | Yes |  |
| mexR | multidrug resistance operon repressor MexR | - | - | - | - | SNV | Gln137Glu | 409C>G | Yes |  |
| fiuA | ferrichrome receptor FiuA | SNV | - | 1854C>T | No | SNV | - | 1854C>T | No |  |
| fiuA | ferrichrome receptor FiuA | SNV | - | 1842G>C | No | SNV | - | 1842G>C | No |  |
| fiuA | ferrichrome receptor FiuA | SNV | - | 1839G>A | No | SNV | - | 1830G>C | No |  |
| fiuA | ferrichrome receptor FiuA | SNV | - | 1836C>T | No | SNV | Ile608Met | 1824C>G | Yes |  |
| fiuA | ferrichrome receptor FiuA | SNV | - | 1830G>C | No | SNV | Ile608Leu | 1822A>C | Yes |  |
| fiuA | ferrichrome receptor FiuA | SNV | Ile608Met | 1824C>G | Yes | SNV | - | 1812G>A | No |  |
| fiuA | ferrichrome receptor FiuA | SNV | Ile608Leu | 1822A>C | Yes | SNV | - | 1800G>C | No |  |
| fiuA | ferrichrome receptor FiuA | SNV | - | 1812G>A | No | SNV | - | 1797G>A | No |  |
| fiuA | ferrichrome receptor FiuA | SNV | - | 1800G>C | No | SNV | - | 1794G>C | No |  |
| fiuA | ferrichrome receptor FiuA | SNV | - | 1797G>A | No | SNV | - | 1792C>T | No |  |
| fiuA | ferrichrome receptor FiuA | SNV | - | 1794G>C | No | Deletion | Ser597fs | 1790delC | Yes |  |
| fiuA | ferrichrome receptor FiuA | SNV | - | 1792C>T | No | SNV | Ser596Arg | 1788T>A | Yes |  |
| fiuA | ferrichrome receptor FiuA | Deletion | Ser597fs | 1790delC | Yes | Insertion | Ser596fs | 1786dupA | Yes |  |
| fiuA | ferrichrome receptor FiuA | SNV | Ser596Arg | 1788T>A | Yes | SNV | Thr595Asn | 1784C>A | Yes |  |
| fiuA | ferrichrome receptor FiuA | Insertion | Ser596fs | 1786dupA | Yes | MNV |  | 1780_1781delinsTC | No |  |
| fiuA | ferrichrome receptor FiuA | SNV | Thr595Asn | 1784C>A | Yes | Insertion | Asn593fs | 1778_1779insGG | Yes |  |
| fiuA | ferrichrome receptor FiuA | MNV | - | 1780_1781delinsTC | No | MNV | Met592_Asn593delinsIleTyr | 1776_1777delinsAT | Yes |  |
| fiuA | ferrichrome receptor FiuA | Insertion | Asn593fs | 1778_1779insGG | Yes | Replacement | Met592fs | 1773delinsCG | Yes |  |
| fiuA | ferrichrome receptor FiuA | MNV | Met592_Asn593delinsIleTyr | 1776_1777delinsAT | Yes | - | - | - | - |  |
| fiuA | ferrichrome receptor FiuA | Replacement | Met592fs | 1773delinsCG | Yes | - | - | - | - |  |
| ostA | organic solvent tolerance protein OstA | SNV | - | 1545A>G | No | SNV | - | 1563C>T | No |  |
| ostA | organic solvent tolerance protein OstA | - | - | - | - | SNV | Glu519Asp | 1557A>T | Yes |  |
| PA0666 | anhydro-N-acetylmuramic acid kinase | - | - | - | - | SNV | Ala140Thr | 418G>A | Yes |  |
| PA0728 | bacteriophage integrase | MNV | Ala45Thr | 132_133delinsCA | Yes | - | - | - | - |  |
| PA0728 | bacteriophage integrase | SNV | - | 135G>A | No | - | - | - | - |  |
| PA0728 | bacteriophage integrase | SNV | - | 138A>G | No | - | - | - | - |  |
| PA0728 | bacteriophage integrase | Deletion | Cys49fs | 144delC | Yes | - | - | - | - |  |
| PA0728 | bacteriophage integrase | MNV | Cys49Gly | 144_145delinsTG | Yes | - | - | - | - |  |
| PA0728 | bacteriophage integrase | Insertion | Trp50fs | 147_148insG | Yes | - | - | - | - |  |
| PA0728 | bacteriophage integrase | Deletion | Thr51fs | 152delC | Yes | - | - | - | - |  |
| PA0728 | bacteriophage integrase | Insertion | Leu52fs | 154_155insA | Yes | - | - | - | - |  |
| PA0728 | bacteriophage integrase | MNV | Arg53Lys | 158_159delinsAA | Yes | - | - | - | - |  |
| PA0728 | bacteriophage integrase | SNV | - | 162G>C | No | - | - | - | - |  |
| PA0728 | bacteriophage integrase | SNV | - | 168C>T | No | - | - | - | - |  |
| PA0728 | bacteriophage integrase | SNV | - | 171T>C | No | - | - | - | - |  |
| PA0728 | bacteriophage integrase | MNV | Arg59Ser | 174_175delinsAA | Yes | - | - | - | - |  |
| PA0728 | bacteriophage integrase | SNV | - | 177T>G | No | - | - | - | - |  |
| PA0728 | bacteriophage integrase | SNV | - | 183G>C | No | - | - | - | - |  |
| PA0728 | bacteriophage integrase | SNV | - | 187T>C | No | - | - | - | - |  |
| PA0728 | bacteriophage integrase | MNV | Glu65Gln | 192_193delinsGC | Yes | - | - | - | - |  |
| PA0728 | bacteriophage integrase | SNV | Leu66Val | 196C>G | Yes | - | - | - | - |  |
| fliC | B-type flagellin | SNV | - | 21G>C | No | - | - | - | - |  |
| oprD | porin D | - | - | - | - | SNV | Gly183Asp | 548G>A | Yes |  |
| flgK | flagellar hook-associated protein FlgK | - | - | - | - | SNV | Ser461Thr | 1382G>C | Yes |  |
| flgK | flagellar hook-associated protein FlgK | - | - | - | - | MNV | Ala463Lys | 1387_1389delinsAAG | Yes |  |
| PA1509 | immunity protein, TplEi | - | - | - | - | Insertion | Glu204fs | 602dupC | Yes |  |
| pcoB | copper resistance protein B | - | - | - | - | SNV | His76Arg | 227A>G | Yes |  |
| PA2091 | hypothetical protein | - | - | - | - | Deletion | Ala16del | 46_48delGCG | Yes |  |
| PA2092 | major facilitator superfamily transporter | - | - | - | - | Insertion | - | - | - | - |
| pvdD | pyoverdine synthetase D | SNV |  | 4765T>C | No | - | - | - | - |  |
| pvdP | pyoverdine biosynthesis protein PvdP | - | - | - | - | SNV | - | 552T>G | No |  |
| pvdP | pyoverdine biosynthesis protein PvdP | - | - | - | - | SNV | - | 543T>C | No |  |
| pvdP | pyoverdine biosynthesis protein PvdP | - | - | - | - | SNV | Asn178Lys | 534C>G | Yes |  |
| pvdP | pyoverdine biosynthesis protein PvdP | - | - | - | - | SNV | Asn178His | 532A>C | Yes |  |
| pvdP | pyoverdine biosynthesis protein PvdP | - | - | - | - | MNV | Ser176_Pro177delinsGlnAla | 526_529delinsCAAG | Yes |  |
| pvdP | pyoverdine biosynthesis protein PvdP | - | - | - | - | SNV |  | 309T>C | No |  |
| PA2393 | dipeptidase | - | - | - | - | Replacement | Lys29fs | 86_88delinsC | Yes |  |
| PA2393 | dipeptidase | - | - | - | - | MNV | Phe31Ser | 91_93delinsAGT | Yes |  |
| PA2393 | dipeptidase | - | - | - | - | Deletion | Pro33fs | 98delC | Yes |  |
| PA2393 | dipeptidase | - | - | - | - | SNV | Pro33Leu | 98C>T | Yes |  |
| PA2393 | dipeptidase | - | - | - | - | SNV | Ser34Gly | 100A>G | Yes |  |
| PA2393 | dipeptidase | - | - | - | - | MNV | Glu35Ser | 103_105delinsAGC | Yes |  |
| PA2402 | peptide synthase | SNV | - | 1707A>G | No | SNV | - | 10324C>T | No |  |
| PA2402 | peptide synthase | SNV | - | 1605G>A | No | MNV | - | 10251_10252delinsTT | No |  |
| PA2402 | peptide synthase | - | - | - | - | SNV | - | 9933C>G | No |  |
| glyA2 | serine hydroxymethyltransferase | - | - | - | - | SNV | - | 189T>C | No |  |
| PA2435 | cation-transporting P-type ATPase | Deletion | His19del | 48_50delCCA | Yes | - | - | - | - |  |
| PA2462 | hypothetical protein | SNV | - | 14874G>C | No | SNV |  | 14877T>C | No |  |
| PA2462 | hypothetical protein | SNV | - | 14865G>C | No | SNV | Asp3596Gly | 10787A>G | Yes |  |
| PA2462 | hypothetical protein | SNV | Ile4954Leu | 14860A>C | Yes | SNV | - | 9318C>T | No |  |
| PA2462 | hypothetical protein | SNV | - | 14847G>A | No | MNV | Val2787Ala | 8360_8361delinsCG | Yes |  |
| PA2462 | hypothetical protein | SNV | - | 9273T>C | No | - | - | - | - |  |
| PA2462 | hypothetical protein | SNV | Ile3091Val | 9271A>G | Yes | - | - | - | - |  |
| PA2462 | hypothetical protein | SNV | Ala2866Thr | 8596G>A | Yes | - | - | - | - |  |
| PA2462 | hypothetical protein | SNV | Val2817Ile | 8449G>A | Yes | - | - | - | - |  |
| PA2462 | hypothetical protein | SNV | - | 963T>C | No | - | - | - | - |  |
| PA2462 | hypothetical protein | SNV | - | 930C>T | No | - | - | - | - |  |
| PA3053 | hydrolase | Deletion | Ser9_Leu10del | 25_30delAGCCTC | Yes | - | - | - | - |  |
| PA3494 | electron transport complex subunit E | Deletion | Gln231_Arg232del | 691_696delCAGCGC | Yes | - | - | - | - |  |
| wbpM | nucleotide sugar epimerase/dehydratase WbpM | - | - | - | - | Deletion | Val29fs | 81delT | Yes |  |
| wbpM | nucleotide sugar epimerase/dehydratase WbpM | - | - | - | - | SNV | Thr27Ala | 79A>G | Yes |  |
| wbpM | nucleotide sugar epimerase/dehydratase WbpM | - | - | - | - | Insertion | Thr27fs | 77_78insC | Yes |  |
| wbpM | nucleotide sugar epimerase/dehydratase WbpM | - | - | - | - | MNV | Val26Met | 75_76delinsCA | Yes |  |
| wbpM | nucleotide sugar epimerase/dehydratase WbpM | - | - | - | - | SNV | - | 72C>G | No |  |
| wbpM | nucleotide sugar epimerase/dehydratase WbpM | - | - | - | - | MNV | Ala24Thr | 69_70delinsGA | Yes |  |
| tufA | elongation factor Tu | - | - | - | - | SNV | - | 1038A>G | No |  |
| tufB | elongation factor Tu | SNV |  | 423C>T | No | - | - | - | - |  |
| pctB | chemotactic transducer PctB | - | - | - | - | SNV | - | 1338C>T | No |  |
| PA4503 | dipeptide ABC transporter permease DppB | SNV | - | 411T>C | No | - | - | - | - |  |
| PA4514 | iron transport outer membrane receptor | SNV | Thr193Ile | 578C>T | Yes | SNV | Thr193Ile | 578C>T | Yes |  |
| PA4514 | iron transport outer membrane receptor | Replacement | Asp190fs | 569_570delinsG | Yes | Replacement | Asp190fs | 569_570delinsG | Yes |  |
| PA4514 | iron transport outer membrane receptor | SNV | Gln189Leu | 566A>T | Yes | SNV | Gln189Leu | 566A>T | Yes |  |
| PA4514 | iron transport outer membrane receptor | Insertion | Gln189fs | 564dupG | Yes | Insertion | Gln189fs | 564dupG | Yes |  |
| pilA | type 4 fimbrial protein PilA | SNV | Asn32Asp | 94A>G | Yes | SNV | - | 60T>C | No |  |
| pilA | type 4 fimbrial protein PilA | SNV | - | 84C>G | No | SNV | - | 48T>C | No |  |
| pilA | type 4 fimbrial protein PilA | SNV | - | 81T>C | No | MNV | - | 24_25delinsTC | No |  |
| pilA | type 4 fimbrial protein PilA | - | - | - | - | SNV | - | 18C>T | No |  |
| pilA | type 4 fimbrial protein PilA | - | - | - | - | SNV | - | 15A>G | No |  |
| pilA | type 4 fimbrial protein PilA | - | - | - | - | SNV | - | 12A>G | No |  |
| PA4541 | Pseudomonas aeruginosa-derived large extracellular protease, LepA | SNV | Ala441Val | 1322C>T | Yes | SNV | Ser445Thr | 1334G>C | Yes |  |
| PA4541 | Pseudomonas aeruginosa-derived large extracellular protease, LepA | SNV | Ile444Val | 1330A>G | Yes | - | - | - | - |  |
| PA4541 | Pseudomonas aeruginosa-derived large extracellular protease, LepA | SNV | Ile444Met | 1332C>G | Yes | - | - | - | - |  |
| PA4541 | Pseudomonas aeruginosa-derived large extracellular protease, LepA | MNV | Ser445Thr | 1334_1335delinsCT | Yes | - | - | - | - |  |
| pilY1 | type 4 fimbrial biogenesis protein PilY1 | - | - | - | - | Deletion | Lys99fs | 294delG | Yes |  |
| PA4625 | cyclic diguanylate-regulated TPS partner A, CdrA | SNV |  | 3577T>C | No | SNV | Ala1590Ser | 4768G>T | Yes |  |
| PA4625 | cyclic diguanylate-regulated TPS partner A, CdrA | Insertion | Leu1172fs | 3513_3514insA | Yes | SNV | - | 4752T>C | No |  |
| PA4625 | cyclic diguanylate-regulated TPS partner A, CdrA | Deletion | Ser1171fs | 3511delA | Yes | SNV | - | 4224T>C | No |  |
| PA4625 | cyclic diguanylate-regulated TPS partner A, CdrA | - | - | - | - | SNV | - | 3783T>C | No |  |
| PA4625 | cyclic diguanylate-regulated TPS partner A, CdrA | - | - | - | - | SNV | Ala1196Val | 3587C>T | Yes |  |
| PA4625 | cyclic diguanylate-regulated TPS partner A, CdrA | - | - | - | - | SNV | - | 3477C>T | No |  |
| PA4625 | cyclic diguanylate-regulated TPS partner A, CdrA | - | - | - | - | SNV | - | 3462G>C | No |  |
| PA5088 | type VI secretion lipase immunity protein, Tli5b3 | SNV | Gly181Ser | 541G>A | Yes | SNV | - | 802T>C | No |  |
| PA5088 | type VI secretion lipase immunity protein, Tli5b3 | SNV | - | 528G>C | No | SNV | Gly181Ser | 541G>A | Yes |  |
| PA5088 | type VI secretion lipase immunity protein, Tli5b3 | SNV | - | 483A>T | No | SNV | - | 528G>C | No |  |
| PA5088 | type VI secretion lipase immunity protein, Tli5b3 | SNV | Arg160Lys | 479G>A | Yes | - | - | - | - |  |
| PA5088 | type VI secretion lipase immunity protein, Tli5b3 | SNV | Leu158Phe | 474A>T | Yes | - | - | - | - |  |
| PA5088 | type VI secretion lipase immunity protein, Tli5b3 | SNV | - | 472T>C | No | - | - | - | - |  |
| PA5088 | type VI secretion lipase immunity protein, Tli5b3 | SNV | - | 429C>G | No | - | - | - | - |  |
| PA5088 | type VI secretion lipase immunity protein, Tli5b3 | SNV | - | 426T>C | No | - | - | - | - |  |
| PA5088 | type VI secretion lipase immunity protein, Tli5b3 | SNV |  | 180T>G | No | - | - | - | - |  |
| PA5167 | C4-dicarboxylate-binding protein | - | - | - | - | Insertion | - | *3_*5dupAGA | No |  |
| algP | alginate regulatory protein AlgP | - | - | - | - | SNV | Val217Ala | 650T>C | Yes |  |

**Table S5.** Single nucleotide polymorphism (SNP) and other genome modifying events identified between the ancestry isolates and the progeny isolates using PAO1 as reference. SNV:Single Nucleotide Variation; MNV: Multi-Nucleotide Variation.
