# Supplementary material for: Pseudomonas aeruginosa modulates alginate biosynthesis and type VI secretion system in two critically ill COVID-19 patients
Source: Cell Biosci. 2022 Feb 9;12:14. doi: 10.1186/s13578-022-00748-z (PMC8827185; doi:10.1186/s13578-022-00748-z)
Supplement: Supplementary file 8 — Additional file 8: Table S6. Full list of DEGs in LYSZa3 comparing to LYSZa2 selected based on the criteria of fold change ≧ 4, adjusted p-value < 0.05 and base mean ≧ 20. [file 13578_2022_748_MOESM8_ESM.docx]

| **Gene** | **Product Name** | **Base Mean** | **Fold Change** | **Adj. p-value** | **LYSZa2 Mean** | **LYSZa3 Mean** |
| --- | --- | --- | --- | --- | --- | --- |
| *algD* | GDP-mannose 6-dehydrogenase AlgD | 27877.94 | 1091.52 | 0.00E+00 | 61.33 | 50065.00 |
| *algX* | alginate biosynthesis protein AlgX | 1967.25 | 256.42 | 2.31E-236 | 18.33 | 3478.67 |
| *algA* | phosphomannose isomerase / guanosine 5'-diphospho-D-mannose pyrophosphorylase | 5853.40 | 238.02 | 0.00E+00 | 59.00 | 10382.00 |
| *algE* | Alginate production outer membrane protein AlgE precursor | 2204.36 | 221.80 | 3.29E-252 | 24.00 | 3889.33 |
| *algF* | alginate o-acetyltransferase AlgF | 1648.75 | 211.59 | 3.00E-242 | 18.67 | 2924.00 |
| *algL* | poly(beta-d-mannuronate) lyase precursor AlgL | 2134.58 | 182.28 | 4.64E-245 | 28.00 | 3766.33 |
| *alg44* | alginate biosynthesis protein Alg44 | 1855.41 | 162.63 | 1.64E-285 | 27.33 | 3260.33 |
| *algJ* | alginate o-acetyltransferase AlgJ | 1033.36 | 154.76 | 2.12E-191 | 16.00 | 1783.33 |
| *algK* | alginate biosynthetic protein AlgK precursor | 1171.78 | 145.20 | 2.18E-233 | 19.33 | 2043.00 |
| *alg8* | alginate biosynthesis protein Alg8 | 2758.04 | 133.33 | 0.00E+00 | 49.67 | 4939.33 |
| *algI* | alginate o-acetyltransferase AlgI | 1457.21 | 59.29 | 1.83E-259 | 58.33 | 2501.67 |
| *algG* | alginate-c5-mannuronan-epimerase AlgG | 1682.20 | 16.89 | 1.73E-125 | 226.00 | 2793.33 |
| PA0736a |  | 81.00 | 9.97 | 1.32E-33 | 17.67 | 131.67 |
| PA1784 | hypothetical protein | 304.20 | 9.45 | 6.05E-84 | 70.33 | 498.67 |
| PA3403a |  | 293.19 | 8.85 | 5.78E-75 | 72.33 | 473.00 |
| PA4154 | conserved hypothetical protein | 875.11 | 8.60 | 4.85E-137 | 220.00 | 1421.00 |
| PA3733a |  | 1267.28 | 8.54 | 9.96E-131 | 319.33 | 2024.67 |
| PA0103 | probable sulfate transporter | 979.48 | 8.28 | 7.41E-119 | 254.00 | 1557.00 |
| PA0738 | conserved hypothetical protein | 34.70 | 7.99 | 6.12E-15 | 9.33 | 56.67 |
| PA0737 | hypothetical protein | 288.57 | 7.59 | 1.67E-70 | 81.67 | 461.67 |
| PA2167 | hypothetical protein | 129.26 | 7.37 | 1.03E-38 | 37.67 | 204.00 |
| PA1108 | probable major facilitator superfamily (MFS) transporter | 91.97 | 7.26 | 4.15E-32 | 26.67 | 143.33 |
| PA0062 | hypothetical protein | 457.48 | 7.22 | 1.77E-108 | 134.00 | 714.33 |
| PA3404 | probable outer membrane protein precursor | 209.66 | 6.83 | 3.97E-65 | 64.67 | 329.67 |
| PA2569 | hypothetical protein | 273.45 | 6.72 | 1.78E-65 | 86.33 | 434.67 |
| PA2046 | hypothetical protein | 399.20 | 6.56 | 1.01E-80 | 127.67 | 606.33 |
| PA2171 | hypothetical protein | 79.13 | 6.52 | 2.91E-18 | 25.67 | 117.33 |
| PA5182 | hypothetical protein | 5011.51 | 6.46 | 3.63E-210 | 1625.67 | 7796.00 |
| PA2778 | hypothetical protein | 1391.76 | 6.34 | 3.44E-90 | 455.00 | 2115.33 |
| PA2562 | hypothetical protein | 4476.87 | 6.04 | 2.04E-112 | 1540.67 | 6868.00 |
| PA1111 | hypothetical protein | 172.57 | 5.99 | 1.85E-40 | 59.67 | 262.67 |
| PA2168 | hypothetical protein | 37.91 | 5.99 | 1.73E-12 | 13.00 | 57.67 |
| PA2779 | hypothetical protein | 1096.58 | 5.90 | 2.62E-57 | 384.67 | 1634.00 |
| PA5212 | hypothetical protein | 2293.21 | 5.86 | 8.85E-112 | 809.67 | 3497.67 |
| PA2021 | hypothetical protein | 64.59 | 5.85 | 1.16E-20 | 22.67 | 98.33 |
| PA3902 | hypothetical protein | 2257.68 | 5.82 | 2.52E-115 | 798.33 | 3443.33 |
| PA2414 | L-sorbosone dehydrogenase | 669.10 | 5.73 | 5.46E-51 | 240.00 | 1003.67 |
| PA4390 | hypothetical protein | 1768.17 | 5.56 | 1.68E-160 | 649.33 | 2692.33 |
| PA3274 | hypothetical protein | 88.76 | 5.43 | 9.53E-21 | 32.67 | 131.00 |
| PA2148 | conserved hypothetical protein | 31.04 | 5.35 | 4.73E-09 | 12.00 | 49.67 |
| *glpD* | glycerol-3-phosphate dehydrogenase | 475.65 | 5.27 | 4.27E-02 | 183.67 | 833.00 |
| PA0102 | probable carbonic anhydrase | 3066.40 | 5.26 | 1.70E-68 | 1180.67 | 4520.33 |
| PA1404 | hypothetical protein | 53.68 | 5.26 | 3.13E-12 | 20.00 | 75.00 |
| PA2159 | conserved hypothetical protein | 43.75 | 5.26 | 9.15E-14 | 16.67 | 63.67 |
| PA2134 | hypothetical protein | 53.89 | 5.24 | 1.04E-15 | 21.00 | 81.00 |
| *plcR* | phospholipase accessory protein PlcR precursor | 85.72 | 5.05 | 1.63E-20 | 34.67 | 127.67 |
| PA2415 | hypothetical protein | 132.05 | 5.04 | 1.61E-26 | 52.67 | 195.33 |
| PA4153 | 2,3-butanediol dehydrogenase | 135.20 | 5.01 | 7.08E-29 | 55.00 | 209.33 |
| *ppgL* | periplasmic gluconolactonase, PpgL | 1520.07 | 5.00 | 2.35E-75 | 614.33 | 2255.67 |
| PA5526 | hypothetical protein | 771.92 | 5.00 | 7.81E-78 | 307.33 | 1115.67 |
| PA2485 | hypothetical protein | 183.48 | 4.91 | 2.25E-31 | 74.33 | 263.67 |
| *treA* | periplasmic trehalase precursor | 228.96 | 4.89 | 8.56E-27 | 94.00 | 331.33 |
| PA5183 | hypothetical protein | 863.33 | 4.88 | 2.95E-111 | 355.67 | 1285.00 |
| PA1870 | hypothetical protein | 133.89 | 4.80 | 9.07E-33 | 55.67 | 196.67 |
| PA4172 | probable nuclease | 75.13 | 4.74 | 1.44E-15 | 31.00 | 110.67 |
| PA3177 | diguanylate cyclase | 1482.89 | 4.62 | 1.92E-120 | 637.67 | 2158.67 |
| PA4713 | hypothetical protein | 299.89 | 4.60 | 1.11E-59 | 129.33 | 442.00 |
| PA4338 | hypothetical protein | 500.39 | 4.58 | 1.30E-68 | 217.67 | 734.33 |
| PA1592 | hypothetical protein | 3974.76 | 4.56 | 1.31E-113 | 1719.00 | 5704.00 |
| *poxB* | pyruvate dehydrogenase (cytochrome) | 1278.92 | 4.56 | 4.30E-93 | 555.67 | 1880.00 |
| *fecA* | Fe(III) dicitrate transport protein FecA | 1220.58 | 4.55 | 1.78E-106 | 532.00 | 1814.33 |
| PA3460 | probable acetyltransferase | 1920.10 | 4.55 | 2.35E-78 | 831.33 | 2757.00 |
| PA0990 | conserved hypothetical protein | 235.22 | 4.49 | 1.12E-22 | 104.00 | 345.33 |
| PA2158 | probable alcohol dehydrogenase (Zn-dependent) | 59.83 | 4.32 | 1.84E-12 | 26.67 | 81.33 |
| PA2176 | hypothetical protein | 144.23 | 4.29 | 1.43E-21 | 66.67 | 211.00 |
| PA0543 | hypothetical protein | 276.20 | 4.26 | 4.02E-33 | 127.33 | 389.67 |
| PA4345 | hypothetical protein | 747.22 | 4.25 | 9.15E-50 | 346.00 | 1084.67 |
| *osmC* | osmotically inducible protein OsmC | 1314.24 | 4.22 | 8.56E-50 | 610.00 | 1889.67 |
| PA2180 | hypothetical protein | 45.42 | 4.20 | 4.17E-11 | 21.00 | 64.33 |
| PA2172 | hypothetical protein | 73.42 | 4.12 | 1.18E-16 | 34.33 | 104.67 |
| PA3952 | hypothetical protein | 1646.51 | 4.12 | 6.70E-65 | 776.00 | 2339.67 |
| PA1889 | hypothetical protein | 1056.74 | 4.11 | 1.93E-77 | 500.00 | 1545.33 |
| PA0490 | hypothetical protein | 328.29 | 4.03 | 1.55E-48 | 157.33 | 463.67 |
| PA0329 | conserved hypothetical protein | 3795.42 | 4.02 | 5.30E-65 | 1827.33 | 5395.67 |
| PA3461 | conserved hypothetical protein | 736.41 | 4.02 | 1.89E-40 | 352.67 | 1033.67 |
| PA3369 | hypothetical protein | 114.41 | -4.11 | 3.73E-20 | 219.67 | 39.67 |
| PA0178 | probable two-component sensor | 417.91 | -4.17 | 5.59E-32 | 817.67 | 141.00 |
| *phzS* | flavin-containing monooxygenase | 412.68 | -4.24 | 1.93E-08 | 810.33 | 136.67 |
| *pilN* | type 4 fimbrial biogenesis protein PilN | 612.60 | -4.27 | 5.59E-82 | 1195.67 | 207.00 |
| PA3371 | hypothetical protein | 22.19 | -4.34 | 1.62E-05 | 45.00 | 8.00 |
| PA4131 | probable iron-sulfur protein | 418.39 | -4.38 | 7.94E-07 | 836.00 | 141.33 |
| *hcpB* | secreted protein Hcp | 107.95 | -4.46 | 1.17E-20 | 215.67 | 36.00 |
| PA1663 | Sfa2 | 305.43 | -4.46 | 2.74E-47 | 603.33 | 100.00 |
| PA1132 | hypothetical protein | 281.92 | -4.47 | 5.27E-42 | 553.33 | 92.33 |
| *phzM* | probable phenazine-specific methyltransferase | 311.70 | -4.48 | 6.26E-20 | 615.00 | 97.67 |
| PA1666 | Lip2 | 159.33 | -4.50 | 2.97E-24 | 319.00 | 52.67 |
| PA2362 | DotU3 | 22.49 | -4.54 | 4.95E-06 | 44.00 | 7.00 |
| *pctC* | chemotactic transducer PctC | 1130.92 | -4.57 | 2.79E-101 | 2236.33 | 365.33 |
| *pcrH* | regulatory protein PcrH | 26.72 | -4.58 | 8.42E-07 | 54.33 | 9.00 |
| PA1660 | HsiG2 | 346.81 | -4.63 | 1.56E-59 | 686.67 | 110.33 |
| PA1662 | clpV2 | 1014.01 | -4.63 | 3.41E-44 | 2020.67 | 318.00 |
| PA1661 | HsiH2 | 246.25 | -4.65 | 1.19E-39 | 488.33 | 77.67 |
| PA2747 | hypothetical protein | 201.48 | -4.72 | 1.01E-37 | 398.67 | 62.33 |
| PA0047 | hypothetical protein | 260.39 | -4.82 | 7.55E-45 | 520.00 | 80.33 |
| PA0573 | hypothetical protein | 56.40 | -4.85 | 1.74E-14 | 113.33 | 17.67 |
| PA2462 | hypothetical protein | 2908.99 | -5.12 | 5.75E-140 | 5888.67 | 848.00 |
| PA0046 | hypothetical protein | 226.20 | -5.15 | 6.71E-36 | 452.33 | 63.67 |
| PA2867 | probable chemotaxis transducer | 1969.65 | -5.16 | 2.59E-150 | 3980.33 | 567.67 |
| PA0045 | hypothetical protein | 571.61 | -5.17 | 1.32E-52 | 1152.67 | 168.00 |
| PA2069 | probable carbamoyl transferase | 216.56 | -5.47 | 4.07E-24 | 443.33 | 58.33 |
| PA1659 | HsiF2 | 163.52 | -5.59 | 1.38E-34 | 334.00 | 43.00 |
| PA0179 | probable two-component response regulator | 210.49 | -5.69 | 6.80E-44 | 432.67 | 55.33 |
| PA1657 | HsiB2 | 842.21 | -6.21 | 6.69E-109 | 1743.67 | 204.33 |
| PA1658 | HsiC2 | 1839.66 | -6.47 | 4.92E-141 | 3824.67 | 437.00 |
| *phzB1* | probable phenazine biosynthesis protein | 181.58 | -6.94 | 2.98E-07 | 380.33 | 38.00 |
| *phzC1* | phenazine biosynthesis protein PhzC | 45.69 | -6.98 | 4.95E-04 | 95.67 | 10.00 |
| PA0122 | rahU | 494.56 | -7.37 | 7.03E-58 | 1052.67 | 104.67 |
| PA4738 | conserved hypothetical protein | 435.05 | -7.58 | 6.95E-67 | 916.00 | 90.67 |
| *phzA1* | probable phenazine biosynthesis protein | 36.17 | -8.03 | 2.80E-13 | 76.33 | 7.00 |
| PA4739 | conserved hypothetical protein | 2298.93 | -8.99 | 4.58E-93 | 4980.67 | 412.33 |
| PA2364 | Lip3 | 32.22 | -9.20 | 6.01E-13 | 70.33 | 5.67 |
| PA4843 | GcbA | 946.57 | -9.36 | 1.88E-182 | 2061.67 | 162.33 |
| PA5481 | hypothetical protein | 899.21 | -9.92 | 5.73E-72 | 1965.33 | 147.33 |
| *pchA* | salicylate biosynthesis isochorismate synthase | 533.10 | -10.50 | 3.64E-118 | 1173.00 | 82.33 |
| *pchC* | pyochelin biosynthetic protein PchC | 93.62 | -14.20 | 2.71E-32 | 209.00 | 10.67 |
| PA5482 | hypothetical protein | 22.83 | -16.86 | 7.36E-10 | 52.00 | 2.33 |
| *pchD* | pyochelin biosynthesis protein PchD | 276.99 | -16.94 | 3.27E-80 | 628.67 | 27.33 |
| *rocA1* | Two-component response regulator RocA1 | 228.52 | -19.83 | 4.22E-75 | 525.00 | 19.33 |
| *pchB* | salicylate biosynthesis protein PchB | 89.04 | -20.58 | 6.52E-33 | 203.67 | 7.33 |
| *fptA* | Fe(III)-pyochelin outer membrane receptor precursor | 1374.54 | -22.59 | 6.45E-213 | 3153.00 | 103.33 |
| PA4219 | AmpO | 317.41 | -24.25 | 3.13E-97 | 730.33 | 22.33 |
| PA4220 | hypothetical protein | 44.86 | -24.88 | 4.28E-17 | 102.67 | 3.00 |
| PA4218 | AmpP | 363.01 | -26.61 | 5.62E-104 | 838.00 | 23.33 |
| PA4222 | probable ATP-binding component of ABC transporter | 581.79 | -26.86 | 3.57E-160 | 1346.67 | 37.00 |
| PA1913 | hypothetical protein | 197.65 | -29.06 | 1.56E-57 | 463.67 | 11.67 |
| *pchF* | pyochelin synthetase | 3869.66 | -37.31 | 0.00E+00 | 9056.67 | 178.67 |
| *pchE* | dihydroaeruginoic acid synthetase | 2807.91 | -39.43 | 1.82E-270 | 6568.67 | 121.67 |
| PA4223 | probable ATP-binding component of ABC transporter | 689.15 | -48.13 | 1.39E-159 | 1620.33 | 25.00 |
| *pchG* | pyochelin biosynthetic protein PchG | 622.41 | -51.41 | 1.04E-144 | 1462.00 | 21.00 |

**Table S6.** Full list of DEGs in LYSZa3 comparing to LYSZa2 selected based on the criteria of fold change ≧4, adjusted p-value<0.05 and base mean ≧ 20.
