# Supplementary material for: Pseudomonas aeruginosa modulates alginate biosynthesis and type VI secretion system in two critically ill COVID-19 patients
Source: Cell Biosci. 2022 Feb 9;12:14. doi: 10.1186/s13578-022-00748-z (PMC8827185; doi:10.1186/s13578-022-00748-z)
Supplement: Supplementary file 9 — Additional file 9: Table S7. Full list of DEGs in LYSZa6 comparing to LYSZa5 selected based on the criteria of fold change ≧ 4, adjusted p-value < 0.05 and base mean ≧ 20. [file 13578_2022_748_MOESM9_ESM.docx]

| **Gene** | **Product Name** | **Base Mean** | **Fold**  **Change** | **Adj. p-value** | **LYSZa5 Mean** | **LYSZa6 Mean** |
| --- | --- | --- | --- | --- | --- | --- |
| *algD* | GDP-mannose 6-dehydrogenase AlgD | 2956.90 | 138.79 | 2.47E-170 | 39.33 | 6375.00 |
| *algX* | alginate biosynthesis protein AlgX | 271.80 | 45.97 | 1.05E-75 | 10.67 | 585.67 |
| *algL* | poly(beta-d-mannuronate) lyase precursor AlgL | 283.61 | 44.85 | 1.98E-74 | 11.33 | 609.00 |
| *narK1* | nitrite extrusion protein 1 | 1381.36 | 39.16 | 7.23E-142 | 65.00 | 2947.67 |
| *algJ* | alginate o-acetyltransferase AlgJ | 118.03 | 32.97 | 3.93E-33 | 6.33 | 248.67 |
| *narK2* | nitrite extrusion protein 2 | 455.06 | 26.44 | 5.77E-78 | 31.33 | 954.67 |
| *algE* | Alginate production outer membrane protein AlgE precursor | 254.48 | 25.19 | 1.11E-61 | 18.33 | 535.33 |
| *algA* | phosphomannose isomerase / guanosine 5'-diphospho-D-mannose pyrophosphorylase | 481.88 | 21.52 | 9.22E-64 | 39.00 | 1000.67 |
| PA0529 | conserved hypothetical protein | 1664.12 | 20.61 | 1.05E-136 | 146.00 | 3529.00 |
| PA2176 | hypothetical protein | 68.05 | 18.88 | 1.02E-26 | 6.33 | 142.00 |
| *algF* | alginate o-acetyltransferase AlgF | 152.00 | 18.68 | 7.50E-42 | 14.33 | 314.67 |
| PA5526 | hypothetical protein | 773.33 | 15.69 | 1.26E-56 | 85.00 | 1647.33 |
| *alg8* | alginate biosynthesis protein Alg8 | 292.51 | 14.91 | 1.09E-59 | 34.67 | 599.33 |
| *alg44* | alginate biosynthesis protein Alg44 | 195.56 | 14.43 | 3.38E-61 | 23.67 | 403.00 |
| *dctA* | C4-dicarboxylate transport protein | 178.34 | 13.39 | 4.04E-48 | 23.00 | 363.67 |
| *osmC* | osmotically inducible protein OsmC | 659.89 | 11.10 | 2.01E-63 | 100.33 | 1368.67 |
| PA2021 | hypothetical protein | 52.52 | 10.96 | 5.53E-18 | 8.33 | 109.00 |
| PA5026 | hypothetical protein | 177.04 | 10.62 | 1.20E-29 | 29.00 | 369.00 |
| PA4154 | conserved hypothetical protein | 404.21 | 10.47 | 7.04E-82 | 66.00 | 817.67 |
| *algK* | alginate biosynthetic protein AlgK precursor | 135.65 | 10.35 | 6.37E-39 | 21.67 | 272.67 |
| *mexB* | Resistance-Nodulation-Cell Division (RND) multidrug efflux transporter MexB | 34450.24 | 10.09 | 7.40E-56 | 5812.00 | 68626.33 |
| *oprM* | Major intrinsic multiple antibiotic resistance efflux outer membrane protein OprM precursor | 14781.14 | 10.07 | 4.63E-66 | 2478.33 | 29481.33 |
| PA0273 | probable major facilitator superfamily (MFS) transporter | 187.10 | 9.84 | 1.84E-30 | 33.67 | 378.00 |
| PA0062 | hypothetical protein | 247.08 | 9.68 | 9.40E-40 | 43.00 | 505.67 |
| PA2414 | L-sorbosone dehydrogenase | 270.17 | 9.39 | 5.22E-58 | 48.33 | 535.33 |
| PA4611 | hypothetical protein | 935.23 | 9.00 | 2.36E-10 | 176.00 | 1919.33 |
| PA3403a |  | 166.05 | 8.99 | 4.95E-38 | 31.67 | 333.67 |
| PA2415 | hypothetical protein | 39.73 | 8.64 | 1.83E-13 | 7.67 | 77.67 |
| *mexA* | Resistance-Nodulation-Cell Division (RND) multidrug efflux membrane fusion protein MexA precursor | 11812.38 | 8.63 | 4.48E-95 | 2257.33 | 23426.00 |
| PA5475 | hypothetical protein | 6997.11 | 8.51 | 6.01E-37 | 1398.33 | 14050.67 |
| *algI* | alginate o-acetyltransferase AlgI | 218.14 | 8.40 | 7.47E-47 | 43.00 | 427.33 |
| *moaB1* | molybdopterin biosynthetic protein B1 | 215.12 | 8.02 | 1.63E-52 | 44.00 | 420.67 |
| *rfaD* | ADP-L-glycero-D-mannoheptose 6-epimerase | 4012.59 | 7.97 | 1.33E-62 | 849.33 | 7935.33 |
| PA2260 | hypothetical protein | 62.86 | 7.93 | 1.36E-21 | 13.00 | 123.00 |
| PA0736a |  | 73.07 | 7.35 | 1.11E-18 | 16.67 | 145.00 |
| *ppyR* | psl and pyoverdine operon regulator, PpyR | 29.75 | 7.26 | 1.33E-10 | 6.67 | 58.33 |
| PA0530 | probable class III pyridoxal phosphate-dependent aminotransferase | 699.23 | 7.09 | 4.28E-82 | 162.33 | 1358.67 |
| *moeA1* | molybdenum cofactor biosynthetic protein A1 | 248.69 | 6.96 | 1.49E-36 | 58.00 | 475.33 |
| PA0103 | probable sulfate transporter | 394.80 | 6.87 | 1.57E-42 | 94.33 | 752.33 |
| PA5027 | hypothetical protein | 1145.32 | 6.79 | 2.53E-46 | 283.33 | 2200.67 |
| PA2134 | hypothetical protein | 26.15 | 6.65 | 1.37E-09 | 6.33 | 50.33 |
| PA4352 | conserved hypothetical protein | 7855.63 | 6.63 | 8.47E-22 | 1949.67 | 15316.67 |
| *arcD* | arginine/ornithine antiporter | 18210.07 | 6.59 | 2.42E-21 | 4487.67 | 35051.00 |
| PA2486 | Pseudomonas type III repressor gene C, PtrC | 39.38 | 6.30 | 1.78E-12 | 10.33 | 75.67 |
| PA3274 | hypothetical protein | 45.29 | 6.27 | 1.70E-12 | 11.67 | 88.33 |
| PA2046 | hypothetical protein | 166.92 | 6.19 | 9.68E-37 | 43.00 | 320.00 |
| PA1784 | hypothetical protein | 109.39 | 6.18 | 7.97E-28 | 28.33 | 208.33 |
| PA3572 | hypothetical protein | 508.73 | 6.10 | 5.09E-08 | 142.33 | 985.00 |
| *adhA* | alcohol dehydrogenase | 4327.62 | 5.95 | 8.77E-45 | 1176.00 | 8169.67 |
| PA2136 | hypothetical protein | 39.09 | 5.90 | 2.10E-11 | 10.67 | 73.67 |
| PA3309 | conserved hypothetical protein | 13326.06 | 5.83 | 6.68E-17 | 3681.67 | 25716.33 |
| PA5525 | probable transcriptional regulator | 1049.73 | 5.77 | 1.20E-39 | 288.00 | 2016.00 |
| *arcA* | arginine deiminase | 6852.67 | 5.77 | 1.52E-26 | 1886.00 | 12676.00 |
| PA1429 | probable cation-transporting P-type ATPase | 3461.28 | 5.76 | 1.06E-26 | 974.00 | 6554.33 |
| PA0200 | hypothetical protein | 637.98 | 5.70 | 1.84E-05 | 179.67 | 1248.33 |
| PA3069 | hypothetical protein | 370.25 | 5.68 | 1.41E-31 | 104.33 | 707.33 |
| PA1196 | transcriptional regulator DdaR | 668.32 | 5.67 | 1.24E-84 | 188.00 | 1260.00 |
| PA3902 | hypothetical protein | 1145.83 | 5.56 | 3.62E-44 | 324.00 | 2191.00 |
| PA4610 | hypothetical protein | 411.49 | 5.53 | 1.17E-21 | 122.00 | 788.33 |
| PA3613 | hypothetical protein | 4056.71 | 5.53 | 4.76E-39 | 1182.00 | 7557.33 |
| PA3273 | hypothetical protein | 32.87 | 5.49 | 7.79E-10 | 9.67 | 62.00 |
| PA0526 | hypothetical protein | 69.90 | 5.47 | 6.08E-15 | 21.00 | 132.33 |
| *narG* | respiratory nitrate reductase alpha chain | 494.15 | 5.45 | 4.74E-54 | 143.00 | 916.67 |
| PA3614 | hypothetical protein | 2335.24 | 5.45 | 3.21E-73 | 675.00 | 4342.33 |
| PA5424 | conserved hypothetical protein | 1715.57 | 5.36 | 1.64E-29 | 511.67 | 3263.00 |
| PA2562 | hypothetical protein | 2551.06 | 5.31 | 1.55E-88 | 755.67 | 4785.33 |
| PA1673 | hypothetical protein | 2803.88 | 5.30 | 1.65E-24 | 841.67 | 5356.33 |
| *ccpR* | cytochrome c551 peroxidase precursor | 1330.90 | 5.18 | 2.19E-28 | 414.00 | 2440.67 |
| PA2501 | hypothetical protein | 130.67 | 5.17 | 1.20E-21 | 40.33 | 247.00 |
| PA3459 | probable glutamine amidotransferase | 2201.04 | 5.16 | 1.25E-75 | 660.00 | 4072.00 |
| PA2175 | hypothetical protein | 28.17 | 5.04 | 2.65E-07 | 8.67 | 52.33 |
| PA0052 | hypothetical protein | 80.95 | 4.95 | 1.23E-18 | 25.00 | 148.67 |
| PA0449 | hypothetical protein | 431.36 | 4.82 | 1.88E-51 | 138.33 | 796.33 |
| PA0737 | hypothetical protein | 127.94 | 4.75 | 1.58E-20 | 42.00 | 234.67 |
| PA2485 | hypothetical protein | 140.25 | 4.74 | 1.52E-16 | 45.67 | 261.00 |
| PA3458 | probable transcriptional regulator | 536.66 | 4.72 | 2.37E-60 | 176.67 | 982.00 |
| PA4351 | OlsA | 1216.79 | 4.69 | 1.15E-06 | 401.67 | 2214.33 |
| *xdhA* | xanthine dehydrogenase | 134.38 | 4.61 | 6.99E-20 | 45.33 | 243.67 |
| PA2026 | conserved hypothetical protein | 272.73 | 4.59 | 6.36E-20 | 89.67 | 495.33 |
| PA0462 | hypothetical protein | 2186.87 | 4.59 | 2.34E-21 | 720.33 | 4063.33 |
| PA1592 | hypothetical protein | 3727.91 | 4.58 | 3.86E-22 | 1295.33 | 6844.33 |
| PA4918 | nicotinamidase, PcnA | 488.69 | 4.57 | 8.52E-31 | 162.67 | 902.67 |
| PA0531 | probable glutamine amidotransferase | 245.60 | 4.51 | 1.03E-32 | 82.33 | 447.33 |
| *algG* | alginate-c5-mannuronan-epimerase AlgG | 356.62 | 4.45 | 4.15E-53 | 121.33 | 644.33 |
| *plcR* | phospholipase accessory protein PlcR precursor | 39.30 | 4.44 | 2.01E-10 | 13.33 | 71.00 |
| PA1111 | hypothetical protein | 96.37 | 4.43 | 1.88E-18 | 34.00 | 175.67 |
| PA3404 | probable outer membrane protein precursor | 103.65 | 4.39 | 8.26E-20 | 35.67 | 185.33 |
| *ccoO2* | Cytochrome c oxidase, cbb3-type, CcoO subunit | 443.86 | 4.39 | 3.57E-43 | 153.00 | 794.33 |
| PA0141 | conserved hypothetical protein | 1850.99 | 4.36 | 3.54E-22 | 661.33 | 3289.33 |
| PA2753 | hypothetical protein | 1103.67 | 4.35 | 1.11E-05 | 381.00 | 2047.00 |
| PA2779 | hypothetical protein | 635.94 | 4.34 | 2.15E-34 | 226.00 | 1132.67 |
| PA0990 | conserved hypothetical protein | 150.52 | 4.32 | 1.05E-21 | 52.67 | 268.00 |
| PA1789 | hypothetical protein | 2350.12 | 4.32 | 1.44E-49 | 839.33 | 4210.00 |
| PA4577 | hypothetical protein | 1028.16 | 4.31 | 1.78E-06 | 369.00 | 1884.67 |
| PA5212 | hypothetical protein | 1202.56 | 4.30 | 2.66E-20 | 438.67 | 2123.33 |
| PA1641 | hypothetical protein | 61.46 | 4.30 | 1.35E-13 | 21.67 | 111.67 |
| PA2569 | hypothetical protein | 192.79 | 4.30 | 9.98E-23 | 68.00 | 351.67 |
| *nrdD* | class III (anaerobic) ribonucleoside-triphosphate reductase subunit, NrdD | 259.12 | 4.29 | 3.25E-30 | 92.67 | 465.33 |
| PA4311 | conserved hypothetical protein | 559.56 | 4.29 | 1.61E-45 | 197.00 | 997.33 |
| PA4153 | 2,3-butanediol dehydrogenase | 80.00 | 4.28 | 2.82E-17 | 28.33 | 144.67 |
| PA3733a |  | 642.42 | 4.26 | 7.36E-24 | 223.33 | 1171.00 |
| PA2754 | conserved hypothetical protein | 1005.85 | 4.26 | 8.01E-06 | 357.00 | 1823.33 |
| PA3796 | hypothetical protein | 653.38 | 4.23 | 5.52E-29 | 235.33 | 1156.67 |
| PA2411 | probable thioesterase | 114.76 | 4.18 | 3.71E-14 | 40.33 | 202.00 |
| PA1746 | hypothetical protein | 1528.34 | 4.16 | 1.25E-38 | 562.00 | 2765.00 |
| PA2883 | hypothetical protein | 392.09 | 4.15 | 9.88E-14 | 146.33 | 710.67 |
| *pvdH* | L-2,4-diaminobutyrate:2-ketoglutarate 4-aminotransferase, PvdH | 88.03 | 4.13 | 2.35E-10 | 30.67 | 153.33 |
| PA2815 | probable acyl-CoA dehydrogenase | 1534.18 | 4.13 | 4.49E-37 | 560.67 | 2703.33 |
| PA2261 | probable 2-ketogluconate kinase | 72.74 | 4.13 | 2.85E-13 | 25.67 | 129.33 |
| PA2177 | probable sensor/response regulator hybrid | 232.14 | 4.12 | 2.43E-37 | 84.67 | 415.00 |
| PA2412 | conserved hypothetical protein | 30.55 | 4.11 | 1.99E-06 | 11.00 | 53.33 |
| PA0102 | probable carbonic anhydrase | 2111.31 | 4.10 | 1.03E-44 | 755.00 | 3773.33 |
| PA2805 | hypothetical protein | 858.97 | 4.07 | 4.00E-05 | 323.67 | 1551.33 |
| *nirF* | heme d1 biosynthesis protein NirF | 56.70 | 4.00 | 1.96E-10 | 20.33 | 99.00 |
| PA3293 | hypothetical protein | 177.98 | -4.01 | 6.74E-29 | 265.33 | 78.67 |
| PA0099 | type VI effector protein | 40.13 | -4.04 | 1.68E-09 | 61.00 | 17.67 |
| PA1395 | hypothetical protein | 197.94 | -4.05 | 7.70E-29 | 296.33 | 86.67 |
| *clpV1* | ClpV1 | 5315.67 | -4.08 | 7.48E-33 | 7854.33 | 2309.33 |
| PA4490 | MagC | 571.22 | -4.09 | 4.34E-51 | 848.33 | 248.67 |
| PA3906 | co-chaperone, co-TecT | 743.85 | -4.10 | 6.51E-22 | 1139.33 | 319.67 |
| *fptA* | Fe(III)-pyochelin outer membrane receptor precursor | 296.92 | -4.11 | 3.58E-17 | 432.67 | 129.33 |
| PA4332 | SadC | 685.72 | -4.14 | 9.89E-23 | 1034.00 | 300.67 |
| PA3292 | hypothetical protein | 172.80 | -4.15 | 1.40E-24 | 263.00 | 75.33 |
| PA3904 | PAAR4 | 1457.06 | -4.17 | 3.79E-33 | 2178.00 | 631.67 |
| PA0172 | SiaA | 689.30 | -4.21 | 6.54E-55 | 1044.00 | 294.33 |
| PA3325 | conserved hypothetical protein | 232.46 | -4.22 | 5.38E-25 | 349.33 | 98.00 |
| PA0122 | rahU | 376.55 | -4.25 | 8.85E-25 | 590.67 | 158.00 |
| PA0048 | probable transcriptional regulator | 169.91 | -4.28 | 6.31E-25 | 255.00 | 71.00 |
| *cysA* | sulfate transport protein CysA | 757.22 | -4.29 | 1.86E-02 | 997.00 | 319.00 |
| *cysW* | sulfate transport protein CysW | 285.74 | -4.33 | 1.53E-02 | 379.00 | 117.67 |
| PA2503 | hypothetical protein | 793.46 | -4.33 | 1.89E-65 | 1203.33 | 331.00 |
| PA2702 | Tse2 | 280.90 | -4.35 | 2.50E-29 | 419.33 | 117.33 |
| *pchF* | pyochelin synthetase | 616.79 | -4.36 | 3.76E-20 | 900.33 | 259.33 |
| PA2774 | Tse4 | 296.61 | -4.37 | 4.85E-26 | 461.67 | 122.33 |
| PA3931 | conserved hypothetical protein | 432.54 | -4.39 | 7.79E-03 | 586.33 | 174.67 |
| PA4689 | hypothetical protein | 4144.48 | -4.43 | 5.15E-48 | 6340.00 | 1677.00 |
| PA2592 | probable periplasmic spermidine/putrescine-binding protein | 1134.11 | -4.46 | 1.82E-32 | 1749.67 | 460.00 |
| PA3021 | hypothetical protein | 637.22 | -4.46 | 1.01E-35 | 974.00 | 255.33 |
| PA1541 | probable drug efflux transporter | 41.87 | -4.48 | 9.71E-05 | 60.67 | 17.00 |
| PA4192 | probable ATP-binding component of ABC transporter | 52.55 | -4.53 | 2.42E-02 | 70.33 | 20.67 |
| PA4222 | probable ATP-binding component of ABC transporter | 125.80 | -4.57 | 9.46E-12 | 183.33 | 51.00 |
| PA2464 | hypothetical protein | 884.65 | -4.58 | 8.34E-26 | 1337.67 | 359.67 |
| PA0041 | probable hemagglutinin | 2536.00 | -4.60 | 5.82E-78 | 3870.67 | 1005.33 |
| PA1133 | hypothetical protein | 156.08 | -4.60 | 3.21E-24 | 236.67 | 62.33 |
| PA5114 | hypothetical protein | 3080.53 | -4.63 | 8.50E-73 | 4708.00 | 1216.33 |
| PA2775 | Tsi4 | 133.81 | -4.71 | 7.24E-19 | 212.67 | 52.33 |
| *vgrG1* | VgrG1 | 2637.35 | -4.74 | 1.56E-50 | 3969.33 | 1016.67 |
| PA1639 | hypothetical protein | 632.83 | -4.79 | 1.08E-74 | 971.67 | 243.00 |
| PA2539 | conserved hypothetical protein | 387.11 | -4.82 | 8.29E-49 | 590.00 | 147.00 |
| PA0938 | Wzz2 | 3475.47 | -4.85 | 7.57E-55 | 5374.33 | 1329.33 |
| PA0093 | Tse6 | 947.79 | -4.91 | 3.88E-46 | 1469.00 | 360.00 |
| *glpF* | glycerol uptake facilitator protein | 397.14 | -4.92 | 4.63E-16 | 573.00 | 149.00 |
| PA1396 | probable two-component sensor | 720.62 | -4.94 | 4.86E-29 | 1119.67 | 274.00 |
| PA3905 | type VI effector chaperone for Tox-Rease, TecT | 1073.56 | -4.95 | 1.10E-53 | 1671.00 | 403.00 |
| PA4223 | probable ATP-binding component of ABC transporter | 124.25 | -5.03 | 2.62E-13 | 186.33 | 46.67 |
| PA2538 | hypothetical protein | 118.82 | -5.03 | 3.63E-22 | 181.00 | 44.00 |
| PA1869 | Acp1 | 422.64 | -5.04 | 2.12E-17 | 677.67 | 157.00 |
| *nalC* | NalC | 802.40 | -5.06 | 4.39E-73 | 1260.67 | 293.00 |
| PA3327 | probable non-ribosomal peptide synthetase | 4055.93 | -5.11 | 2.22E-48 | 6321.67 | 1459.33 |
| PA3580 | conserved hypothetical protein | 297.69 | -5.13 | 7.64E-16 | 435.00 | 108.00 |
| *ibpA* | heat-shock protein IbpA | 583.44 | -5.17 | 2.95E-22 | 873.33 | 212.00 |
| PA4219 | AmpO | 65.90 | -5.19 | 8.85E-12 | 100.33 | 24.00 |
| PA2581 | hypothetical protein | 1471.29 | -5.25 | 1.65E-31 | 2307.00 | 521.00 |
| PA0082 | TssA1 | 1449.46 | -5.32 | 1.76E-55 | 2265.00 | 515.00 |
| PA2540 | conserved hypothetical protein | 1095.49 | -5.32 | 1.13E-38 | 1707.00 | 386.33 |
| PA4843 | GcbA | 1252.21 | -5.35 | 1.12E-39 | 1965.00 | 438.67 |
| PA2594 | conserved hypothetical protein | 175.80 | -5.36 | 9.32E-36 | 272.67 | 61.67 |
| PA2703 | Tsi2 | 98.26 | -5.56 | 5.99E-24 | 156.67 | 33.00 |
| *phzC1* | phenazine biosynthesis protein PhzC | 26.15 | -5.68 | 3.01E-09 | 41.33 | 8.67 |
| PA1134 | hypothetical protein | 83.57 | -5.79 | 3.96E-22 | 133.67 | 27.33 |
| PA0094 | EagT6 | 251.06 | -5.79 | 1.43E-38 | 401.67 | 82.00 |
| PA3484 | Tse3 | 544.56 | -5.86 | 1.50E-54 | 869.33 | 174.33 |
| *sbp* | sulfate-binding protein precursor | 413.50 | -5.90 | 1.73E-04 | 587.33 | 133.00 |
| *stk1* | Stk1 | 263.85 | -5.90 | 1.38E-56 | 423.00 | 84.67 |
| *pchG* | pyochelin biosynthetic protein PchG | 95.75 | -5.94 | 5.44E-19 | 148.33 | 31.00 |
| PA4318 | hypothetical protein | 569.60 | -5.94 | 2.36E-71 | 893.67 | 182.33 |
| PA2376 | probable transcriptional regulator | 95.34 | -6.26 | 9.90E-27 | 154.67 | 29.33 |
| PA2203 | probable amino acid permease | 54.10 | -6.51 | 4.83E-03 | 76.00 | 16.00 |
| *pchE* | dihydroaeruginoic acid synthetase | 539.02 | -6.51 | 5.41E-37 | 852.67 | 161.00 |
| *femI* | ECF sigma factor, FemI | 36.15 | -6.56 | 1.95E-13 | 59.00 | 10.67 |
| PA3850 | hypothetical protein | 1060.38 | -6.61 | 1.60E-49 | 1707.67 | 310.00 |
| PA2462 | hypothetical protein | 5984.60 | -6.67 | 1.54E-100 | 9779.00 | 1732.67 |
| PA5033 | hypothetical protein | 993.22 | -6.71 | 2.00E-83 | 1624.00 | 285.67 |
| PA4690 |  | 799.03 | -6.80 | 1.98E-69 | 1288.00 | 228.67 |
| PA3328 | probable FAD-dependent monooxygenase | 693.70 | -6.81 | 6.72E-32 | 1113.00 | 192.67 |
| PA5266 | VgrG6 | 396.48 | -6.81 | 6.50E-47 | 654.33 | 113.33 |
| PA3329 | hypothetical protein | 718.36 | -6.84 | 8.94E-33 | 1145.00 | 199.00 |
| PA1667 | HsiJ2 | 678.54 | -6.99 | 2.41E-100 | 1119.67 | 188.67 |
| PA1656 | HsiA2 | 3257.03 | -7.01 | 4.84E-80 | 5349.67 | 911.33 |
| PA4195 | probable binding protein component of ABC transporter | 150.36 | -7.03 | 9.20E-05 | 221.00 | 41.67 |
| PA3330 | probable short chain dehydrogenase | 739.38 | -7.12 | 4.39E-35 | 1186.33 | 198.33 |
| PA3331 | cytochrome P450 | 929.44 | -7.23 | 8.64E-32 | 1502.00 | 244.33 |
| PA2450 | hypothetical protein | 315.28 | -7.25 | 5.59E-32 | 504.00 | 86.33 |
| PA1791 | hypothetical protein | 1670.17 | -7.29 | 1.65E-47 | 2753.33 | 444.33 |
| PA0563 | conserved hypothetical protein | 4269.95 | -7.31 | 2.35E-24 | 6852.67 | 1175.33 |
| PA1132 | hypothetical protein | 847.09 | -7.47 | 1.88E-51 | 1374.67 | 225.33 |
| *qteE* | quorum threshold expression element, QteE | 118.00 | -7.64 | 9.87E-35 | 199.00 | 30.33 |
| PA5482 | hypothetical protein | 43.16 | -7.76 | 1.00E-12 | 73.00 | 11.00 |
| PA5481 | hypothetical protein | 815.71 | -7.79 | 7.22E-80 | 1334.00 | 206.33 |
| PA0040 | conserved hypothetical protein | 290.96 | -7.80 | 2.78E-53 | 479.33 | 73.67 |
| *glpT* | glycerol-3-phosphate transporter | 2624.89 | -7.92 | 8.54E-10 | 4087.33 | 659.00 |
| PA3332 | conserved hypothetical protein | 398.44 | -8.32 | 3.39E-30 | 652.00 | 92.33 |
| *desB* | acyl-CoA delta-9-desaturase, DesB | 216.23 | -8.35 | 1.80E-06 | 330.00 | 51.67 |
| PA0989 | hypothetical protein | 243.72 | -8.57 | 2.42E-60 | 400.33 | 56.67 |
| PA2463 | hypothetical protein | 449.82 | -8.78 | 1.00E-74 | 760.67 | 102.33 |
| PA0045 | hypothetical protein | 1674.38 | -8.80 | 1.97E-67 | 2785.00 | 380.67 |
| PA1662 | clpV2 | 2538.08 | -8.98 | 3.88E-108 | 4332.33 | 564.33 |
| PA4317 | hypothetical protein | 1420.91 | -9.63 | 4.65E-132 | 2371.00 | 296.33 |
| PA0047 | hypothetical protein | 968.35 | -9.68 | 1.15E-71 | 1634.33 | 200.00 |
| PA1661 | HsiH2 | 693.51 | -9.91 | 1.55E-110 | 1186.67 | 142.00 |
| *glpK* | glycerol kinase | 1988.47 | -10.10 | 3.65E-13 | 3200.33 | 397.33 |
| *phzA1* | probable phenazine biosynthesis protein | 44.23 | -10.33 | 7.65E-14 | 73.33 | 8.67 |
| PA1666 | Lip2 | 340.97 | -10.48 | 2.48E-72 | 589.00 | 65.67 |
| *stp1* | Stp1 | 327.95 | -10.61 | 1.67E-70 | 562.67 | 62.67 |
| PA0046 | hypothetical protein | 762.12 | -10.68 | 1.69E-64 | 1294.67 | 143.67 |
| *hcpA* | secreted protein Hcp | 72.34 | -10.75 | 7.25E-26 | 125.33 | 13.67 |
| *phzB1* | probable phenazine biosynthesis protein | 89.65 | -10.84 | 3.60E-29 | 150.00 | 16.67 |
| *fabH2* | 3-oxoacyl-[acyl-carrier-protein] synthase III | 843.14 | -10.89 | 2.01E-104 | 1420.33 | 156.00 |
| PA1665 | Fha2 | 840.73 | -10.96 | 1.12E-108 | 1456.67 | 155.33 |
| PA1660 | HsiG2 | 907.03 | -11.05 | 1.78E-125 | 1551.67 | 167.33 |
| PA1669 | IcmF2 | 2052.74 | -11.08 | 6.20E-107 | 3547.33 | 375.67 |
| *hcpB* | secreted protein Hcp | 204.27 | -11.30 | 8.81E-47 | 358.00 | 36.67 |
| PA1668 | DotU2 | 487.58 | -11.30 | 1.39E-105 | 836.33 | 88.00 |
| PA4738 | conserved hypothetical protein | 932.69 | -12.21 | 3.32E-53 | 1629.00 | 157.67 |
| PA4889 | probable oxidoreductase | 358.65 | -12.53 | 7.06E-07 | 562.33 | 60.00 |
| PA3722 | hypothetical protein | 956.36 | -12.73 | 3.40E-123 | 1678.00 | 153.67 |
| PA1663 | Sfa2 | 883.17 | -12.92 | 4.47E-74 | 1556.67 | 139.33 |
| *glpD* | glycerol-3-phosphate dehydrogenase | 2178.49 | -13.26 | 3.50E-04 | 3277.00 | 344.33 |
| PA4739 | conserved hypothetical protein | 4018.09 | -13.54 | 3.27E-13 | 6820.33 | 636.33 |
| PA3334 | Acp3 | 236.04 | -13.79 | 1.82E-73 | 415.67 | 35.33 |
| PA1657 | HsiB2 | 2539.11 | -14.39 | 1.32E-112 | 4445.00 | 366.00 |
| PA1913 | hypothetical protein | 315.66 | -14.85 | 9.38E-60 | 568.33 | 43.67 |
| PA3335 | hypothetical protein | 648.59 | -16.04 | 1.91E-174 | 1138.00 | 84.33 |
| PA1659 | HsiF2 | 480.65 | -17.06 | 4.25E-89 | 843.33 | 59.00 |
| PA1658 | HsiC2 | 5746.91 | -17.11 | 4.82E-66 | 10179.00 | 695.67 |
| PA2204 | probable binding protein component of ABC transporter | 1442.52 | -18.55 | 6.90E-13 | 2342.67 | 160.67 |
| PA3661 | hypothetical protein | 94.63 | -25.16 | 2.45E-35 | 170.67 | 8.00 |
| PA4596 | EsrC | 3532.24 | -29.21 | 7.28E-111 | 6357.00 | 265.00 |
| PA0277 | conserved hypothetical protein | 1824.61 | -33.13 | 1.19E-146 | 3343.00 | 118.33 |
| *oprJ* | Multidrug efflux outer membrane protein OprJ precursor | 17765.55 | -280.92 | 0.00E+00 | 32713.67 | 140.67 |
| *mexD* | Resistance-Nodulation-Cell Division (RND) multidrug efflux transporter MexD | 50537.74 | -435.11 | 0.00E+00 | 93877.33 | 259.00 |
| *mexC* | Resistance-Nodulation-Cell Division (RND) multidrug efflux membrane fusion protein MexC precursor | 23581.84 | -751.35 | 0.00E+00 | 44395.00 | 69.33 |

**Table S7.** Full list of DEGs in LYSZa6 comparing to LYSZa5 selected based on the criteria of fold change ≧ 4, adjusted p-value<0.05 and base mean ≧ 20.
