# Supplementary material for: Pseudomonas aeruginosa modulates alginate biosynthesis and type VI secretion system in two critically ill COVID-19 patients
Source: Cell Biosci. 2022 Feb 9;12:14. doi: 10.1186/s13578-022-00748-z (PMC8827185; doi:10.1186/s13578-022-00748-z)
Supplement: Supplementary file 10 — Additional file 10: Table S8. Common DEGs identified in LYSZa3 and LYSZa6. Filtering criteria are fold change ≧ 4, adjusted p-value < 0.05 and base mean ≧ 20. [file 13578_2022_748_MOESM10_ESM.docx]

|  |  | **LYSZa6 vs LYSZa5** | | | | | **LYSZa3 vs LYSZa2** | | | | |
| --- | --- | --- | --- | --- | --- | --- | --- | --- | --- | --- | --- |
| **Gene** | **Product** | **Base Mean** | **Fold Change** | **Adj. p-value** | **LYSZa5 Mean** | **LYSZa6 Mean** | **Base Mean** | **Fold Change** | **Adj. p-value** | **LYSZa2 Mean** | **LYSZa3 Mean** |
| ***alg44*** | alginate biosynthesis protein Alg44 | 195.56 | 14.43 | 3.38E-61 | 23.67 | 403.00 | 1855.41 | 162.63 | 1.64E-285 | 27.33 | 3260.33 |
| ***alg8*** | alginate biosynthesis protein Alg8 | 292.51 | 14.91 | 1.09E-59 | 34.67 | 599.33 | 2758.04 | 133.33 | 0.00E+00 | 49.67 | 4939.33 |
| ***algA*** | phosphomannose isomerase / guanosine 5'-diphospho-D-mannose pyrophosphorylase | 481.88 | 21.52 | 9.22E-64 | 39.00 | 1000.67 | 5853.40 | 238.02 | 0.00E+00 | 59.00 | 10382.00 |
| ***algD*** | GDP-mannose 6-dehydrogenase AlgD | 2956.90 | 138.79 | 2.47E-170 | 39.33 | 6375.00 | 27877.94 | 1091.52 | 0.00E+00 | 61.33 | 50065.00 |
| ***algE*** | Alginate production outer membrane protein AlgE precursor | 254.48 | 25.19 | 1.11E-61 | 18.33 | 535.33 | 2204.36 | 221.80 | 3.29E-252 | 24.00 | 3889.33 |
| ***algF*** | alginate o-acetyltransferase AlgF | 152.00 | 18.68 | 7.50E-42 | 14.33 | 314.67 | 1648.75 | 211.59 | 3.00E-242 | 18.67 | 2924.00 |
| ***algG*** | alginate-c5-mannuronan-epimerase AlgG | 356.62 | 4.45 | 4.15E-53 | 121.33 | 644.33 | 1682.20 | 16.89 | 1.73E-125 | 226.00 | 2793.33 |
| ***algI*** | alginate o-acetyltransferase AlgI | 218.14 | 8.40 | 7.47E-47 | 43.00 | 427.33 | 1457.21 | 59.29 | 1.83E-259 | 58.33 | 2501.67 |
| ***algJ*** | alginate o-acetyltransferase AlgJ | 118.03 | 32.97 | 3.93E-33 | 6.33 | 248.67 | 1033.36 | 154.76 | 2.12E-191 | 16.00 | 1783.33 |
| ***algK*** | alginate biosynthetic protein AlgK precursor | 135.65 | 10.35 | 6.37E-39 | 21.67 | 272.67 | 1171.78 | 145.20 | 2.18E-233 | 19.33 | 2043.00 |
| ***algL*** | poly(beta-d-mannuronate) lyase precursor AlgL | 283.61 | 44.85 | 1.98E-74 | 11.33 | 609.00 | 2134.58 | 182.28 | 4.64E-245 | 28.00 | 3766.33 |
| ***algX*** | alginate biosynthesis protein AlgX | 271.80 | 45.97 | 1.05E-75 | 10.67 | 585.67 | 1967.25 | 256.42 | 2.31E-236 | 18.33 | 3478.67 |
| ***fptA*** | Fe(III)-pyochelin outer membrane receptor precursor | 296.92 | -4.11 | 3.58E-17 | 432.67 | 129.33 | 1374.54 | -22.59 | 6.45E-213 | 3153.00 | 103.33 |
| ***glpD*** | glycerol-3-phosphate dehydrogenase | 2178.49 | -13.26 | 3.50E-04 | 3277.00 | 344.33 | 475.65 | 5.27 | 4.27E-02 | 183.67 | 833.00 |
| ***hcpB*** | secreted protein Hcp | 204.27 | -11.30 | 8.81E-47 | 358.00 | 36.67 | 107.95 | -4.46 | 1.17E-20 | 215.67 | 36.00 |
| ***osmC*** | osmotically inducible protein OsmC | 659.89 | 11.10 | 2.01E-63 | 100.33 | 1368.67 | 1314.24 | 4.22 | 8.56E-50 | 610.00 | 1889.67 |
| **PA0045** | hypothetical protein | 1674.38 | -8.80 | 1.97E-67 | 2785.00 | 380.67 | 571.61 | -5.17 | 1.32E-52 | 1152.67 | 168.00 |
| **PA0046** | hypothetical protein | 762.12 | -10.68 | 1.69E-64 | 1294.67 | 143.67 | 226.20 | -5.15 | 6.71E-36 | 452.33 | 63.67 |
| **PA0047** | hypothetical protein | 968.35 | -9.68 | 1.15E-71 | 1634.33 | 200.00 | 260.39 | -4.82 | 7.55E-45 | 520.00 | 80.33 |
| **PA0062** | hypothetical protein | 247.08 | 9.68 | 9.40E-40 | 43.00 | 505.67 | 457.48 | 7.22 | 1.77E-108 | 134.00 | 714.33 |
| **PA0102** | probable carbonic anhydrase | 2111.31 | 4.10 | 1.03E-44 | 755.00 | 3773.33 | 3066.40 | 5.26 | 1.70E-68 | 1180.67 | 4520.33 |
| **PA0103** | probable sulfate transporter | 394.80 | 6.87 | 1.57E-42 | 94.33 | 752.33 | 979.48 | 8.28 | 7.41E-119 | 254.00 | 1557.00 |
| **PA0122** | rahU | 376.55 | -4.25 | 8.85E-25 | 590.67 | 158.00 | 494.56 | -7.37 | 7.03E-58 | 1052.67 | 104.67 |
| **PA0736a** |  | 73.07 | 7.35 | 1.11E-18 | 16.67 | 145.00 | 81.00 | 9.97 | 1.32E-33 | 17.67 | 131.67 |
| **PA0737** | hypothetical protein | 127.94 | 4.75 | 1.58E-20 | 42.00 | 234.67 | 288.57 | 7.59 | 1.67E-70 | 81.67 | 461.67 |
| **PA0990** | conserved hypothetical protein | 150.52 | 4.32 | 1.05E-21 | 52.67 | 268.00 | 235.22 | 4.49 | 1.12E-22 | 104.00 | 345.33 |
| **PA1111** | hypothetical protein | 96.37 | 4.43 | 1.88E-18 | 34.00 | 175.67 | 172.57 | 5.99 | 1.85E-40 | 59.67 | 262.67 |
| **PA1132** | hypothetical protein | 847.09 | -7.47 | 1.88E-51 | 1374.67 | 225.33 | 281.92 | -4.47 | 5.27E-42 | 553.33 | 92.33 |
| **PA1592** | hypothetical protein | 3727.91 | 4.58 | 3.86E-22 | 1295.33 | 6844.33 | 3974.76 | 4.56 | 1.31E-113 | 1719.00 | 5704.00 |
| **PA1657** | HsiB2 | 2539.11 | -14.39 | 1.32E-112 | 4445.00 | 366.00 | 842.21 | -6.21 | 6.69E-109 | 1743.67 | 204.33 |
| **PA1658** | HsiC2 | 5746.91 | -17.11 | 4.82E-66 | 10179.00 | 695.67 | 1839.66 | -6.47 | 4.92E-141 | 3824.67 | 437.00 |
| **PA1659** | HsiF2 | 480.65 | -17.06 | 4.25E-89 | 843.33 | 59.00 | 163.52 | -5.59 | 1.38E-34 | 334.00 | 43.00 |
| **PA1660** | HsiG2 | 907.03 | -11.05 | 1.78E-125 | 1551.67 | 167.33 | 346.81 | -4.63 | 1.56E-59 | 686.67 | 110.33 |
| **PA1661** | HsiH2 | 693.51 | -9.91 | 1.55E-110 | 1186.67 | 142.00 | 246.25 | -4.65 | 1.19E-39 | 488.33 | 77.67 |
| **PA1662** | clpV2 | 2538.08 | -8.98 | 3.88E-108 | 4332.33 | 564.33 | 1014.01 | -4.63 | 3.41E-44 | 2020.67 | 318.00 |
| **PA1663** | Sfa2 | 883.17 | -12.92 | 4.47E-74 | 1556.67 | 139.33 | 305.43 | -4.46 | 2.74E-47 | 603.33 | 100.00 |
| **PA1666** | Lip2 | 340.97 | -10.48 | 2.48E-72 | 589.00 | 65.67 | 159.33 | -4.50 | 2.97E-24 | 319.00 | 52.67 |
| **PA1784** | hypothetical protein | 109.39 | 6.18 | 7.97E-28 | 28.33 | 208.33 | 304.20 | 9.45 | 6.05E-84 | 70.33 | 498.67 |
| **PA1913** | hypothetical protein | 315.66 | -14.85 | 9.38E-60 | 568.33 | 43.67 | 197.65 | -29.06 | 1.56E-57 | 463.67 | 11.67 |
| **PA2021** | hypothetical protein | 52.52 | 10.96 | 5.53E-18 | 8.33 | 109.00 | 64.59 | 5.85 | 1.16E-20 | 22.67 | 98.33 |
| **PA2046** | hypothetical protein | 166.92 | 6.19 | 9.68E-37 | 43.00 | 320.00 | 399.20 | 6.56 | 1.01E-80 | 127.67 | 606.33 |
| **PA2134** | hypothetical protein | 26.15 | 6.65 | 1.37E-09 | 6.33 | 50.33 | 53.89 | 5.24 | 1.04E-15 | 21.00 | 81.00 |
| **PA2176** | hypothetical protein | 68.05 | 18.88 | 1.02E-26 | 6.33 | 142.00 | 144.23 | 4.29 | 1.43E-21 | 66.67 | 211.00 |
| **PA2414** | L-sorbosone dehydrogenase | 270.17 | 9.39 | 5.22E-58 | 48.33 | 535.33 | 669.10 | 5.73 | 5.46E-51 | 240.00 | 1003.67 |
| **PA2415** | hypothetical protein | 39.73 | 8.64 | 1.83E-13 | 7.67 | 77.67 | 132.05 | 5.04 | 1.61E-26 | 52.67 | 195.33 |
| **PA2462** | hypothetical protein | 5984.60 | -6.67 | 1.54E-100 | 9779.00 | 1732.67 | 2908.99 | -5.12 | 5.75E-140 | 5888.67 | 848.00 |
| **PA2485** | hypothetical protein | 140.25 | 4.74 | 1.52E-16 | 45.67 | 261.00 | 183.48 | 4.91 | 2.25E-31 | 74.33 | 263.67 |
| **PA2562** | hypothetical protein | 2551.06 | 5.31 | 1.55E-88 | 755.67 | 4785.33 | 4476.87 | 6.04 | 2.04E-112 | 1540.67 | 6868.00 |
| **PA2569** | hypothetical protein | 192.79 | 4.30 | 9.98E-23 | 68.00 | 351.67 | 273.45 | 6.72 | 1.78E-65 | 86.33 | 434.67 |
| **PA2779** | hypothetical protein | 635.94 | 4.34 | 2.15E-34 | 226.00 | 1132.67 | 1096.58 | 5.90 | 2.62E-57 | 384.67 | 1634.00 |
| **PA3274** | hypothetical protein | 45.29 | 6.27 | 1.70E-12 | 11.67 | 88.33 | 88.76 | 5.43 | 9.53E-21 | 32.67 | 131.00 |
| **PA3403a** |  | 166.05 | 8.99 | 4.95E-38 | 31.67 | 333.67 | 293.19 | 8.85 | 5.78E-75 | 72.33 | 473.00 |
| **PA3404** | probable outer membrane protein precursor | 103.65 | 4.39 | 8.26E-20 | 35.67 | 185.33 | 209.66 | 6.83 | 3.97E-65 | 64.67 | 329.67 |
| **PA3733a** |  | 642.42 | 4.26 | 7.36E-24 | 223.33 | 1171.00 | 1267.28 | 8.54 | 9.96E-131 | 319.33 | 2024.67 |
| **PA3902** | hypothetical protein | 1145.83 | 5.56 | 3.62E-44 | 324.00 | 2191.00 | 2257.68 | 5.82 | 2.52E-115 | 798.33 | 3443.33 |
| **PA4153** | 2,3-butanediol dehydrogenase | 80.00 | 4.28 | 2.82E-17 | 28.33 | 144.67 | 135.20 | 5.01 | 7.08E-29 | 55.00 | 209.33 |
| **PA4154** | conserved hypothetical protein | 404.21 | 10.47 | 7.04E-82 | 66.00 | 817.67 | 875.11 | 8.60 | 4.85E-137 | 220.00 | 1421.00 |
| **PA4219** | AmpO | 65.90 | -5.19 | 8.85E-12 | 100.33 | 24.00 | 317.41 | -24.25 | 3.13E-97 | 730.33 | 22.33 |
| **PA4222** | probable ATP-binding component of ABC transporter | 125.80 | -4.57 | 9.46E-12 | 183.33 | 51.00 | 581.79 | -26.86 | 3.57E-160 | 1346.67 | 37.00 |
| **PA4223** | probable ATP-binding component of ABC transporter | 124.25 | -5.03 | 2.62E-13 | 186.33 | 46.67 | 689.15 | -48.13 | 1.39E-159 | 1620.33 | 25.00 |
| **PA4738** | conserved hypothetical protein | 932.69 | -12.21 | 3.32E-53 | 1629.00 | 157.67 | 435.05 | -7.58 | 6.95E-67 | 916.00 | 90.67 |
| **PA4739** | conserved hypothetical protein | 4018.09 | -13.54 | 3.27E-13 | 6820.33 | 636.33 | 2298.93 | -8.99 | 4.58E-93 | 4980.67 | 412.33 |
| **PA4843** | GcbA | 1252.21 | -5.35 | 1.12E-39 | 1965.00 | 438.67 | 946.57 | -9.36 | 1.88E-182 | 2061.67 | 162.33 |
| **PA5212** | hypothetical protein | 1202.56 | 4.30 | 2.66E-20 | 438.67 | 2123.33 | 2293.21 | 5.86 | 8.85E-112 | 809.67 | 3497.67 |
| **PA5481** | hypothetical protein | 815.71 | -7.79 | 7.22E-80 | 1334.00 | 206.33 | 899.21 | -9.92 | 5.73E-72 | 1965.33 | 147.33 |
| **PA5482** | hypothetical protein | 43.16 | -7.76 | 1.00E-12 | 73.00 | 11.00 | 22.83 | -16.86 | 7.36E-10 | 52.00 | 2.33 |
| **PA5526** | hypothetical protein | 773.33 | 15.69 | 1.26E-56 | 85.00 | 1647.33 | 771.92 | 5.00 | 7.81E-78 | 307.33 | 1115.67 |
| ***pchE*** | dihydroaeruginoic acid synthetase | 539.02 | -6.51 | 5.41E-37 | 852.67 | 161.00 | 2807.91 | -39.43 | 1.82E-270 | 6568.67 | 121.67 |
| ***pchF*** | pyochelin synthetase | 616.79 | -4.36 | 3.76E-20 | 900.33 | 259.33 | 3869.66 | -37.31 | 0.00E+00 | 9056.67 | 178.67 |
| ***pchG*** | pyochelin biosynthetic protein PchG | 95.75 | -5.94 | 5.44E-19 | 148.33 | 31.00 | 622.41 | -51.41 | 1.04E-144 | 1462.00 | 21.00 |
| ***phzA1*** | probable phenazine biosynthesis protein | 44.23 | -10.33 | 7.65E-14 | 73.33 | 8.67 | 36.17 | -8.03 | 2.80E-13 | 76.33 | 7.00 |
| ***phzB1*** | probable phenazine biosynthesis protein | 89.65 | -10.84 | 3.60E-29 | 150.00 | 16.67 | 181.58 | -6.94 | 2.98E-07 | 380.33 | 38.00 |
| ***phzC1*** | phenazine biosynthesis protein PhzC | 26.15 | -5.68 | 3.01E-09 | 41.33 | 8.67 | 45.69 | -6.98 | 4.95E-04 | 95.67 | 10.00 |
| ***plcR*** | phospholipase accessory protein PlcR precursor | 39.30 | 4.44 | 2.01E-10 | 13.33 | 71.00 | 85.72 | 5.05 | 1.63E-20 | 34.67 | 127.67 |

**Table S8**. Common DEGs identified in LYSZa3 and LYSZa6. Filtering criteria are fold change ≧ 4, adjusted p-value<0.05 and base mean≧20.
