# Supplementary material for: Pseudomonas aeruginosa modulates alginate biosynthesis and type VI secretion system in two critically ill COVID-19 patients
Source: Cell Biosci. 2022 Feb 9;12:14. doi: 10.1186/s13578-022-00748-z (PMC8827185; doi:10.1186/s13578-022-00748-z)
Supplement: Supplementary file 11 — Additional file 11: Table S9. Detailed information of metagenomics data of the 4 respiratory samples. [file 13578_2022_748_MOESM11_ESM.docx]

| **Sample ID** | **Raw data** | | | **Clean data** | | | **Ratio of reads aligned to the human genome (%)** | **Reads after removal of human host** | | |
| --- | --- | --- | --- | --- | --- | --- | --- | --- | --- | --- |
|  | **Data**  **(Mb)** | **Number of reads** | **Average length**  **(bp)** | **Data**  **(Mb)** | **Number of reads** | **Average length**  **(bp)** |  | **Data**  **(Mb)** | **Number of reads** | **Average length(bp)** |
| LYSZa2 | 955.98 | 19,119,689 | 50 | 821.71 | 19,109,462 | 43 | 72.2% | 228.8 | 1,982,459 | 44 |
| LYSZa3 | 1091.85 | 21,837,036 | 50 | 938.37 | 21,822,522 | 43 | 60.1% | 375.0 | 3,249,664 | 44 |
| LYSZa5 | 1220.17 | 24,403,296 | 50 | 1048.54 | 24,384,664 | 43 | 75.1% | 261.2 | 2,263,437 | 44 |
| LYSZa6 | 1022.91 | 20,458,281 | 50 | 878.39 | 20,427,599 | 43 | 69.5% | 268.1 | 2,322,818 | 44 |

Table S9. Detailed information of metagenomics data of the 4 sputum samples.
