# Supplementary material for: Pseudomonas aeruginosa modulates alginate biosynthesis and type VI secretion system in two critically ill COVID-19 patients
Source: Cell Biosci. 2022 Feb 9;12:14. doi: 10.1186/s13578-022-00748-z (PMC8827185; doi:10.1186/s13578-022-00748-z)
Supplement: Supplementary file 12 — Additional file 12: Table S10. Primers used for RT-PCR tests. [file 13578_2022_748_MOESM12_ESM.docx]

| Gene | Primers | |
| --- | --- | --- |
| *VgrG1* | F | AGATCCGCATGGAGGACAAG |
|  | R | ACCAACAGGGTGTCGTTGAG |
| *hcpA* | F | TGATGGTCCAGGGCTTCAAC |
|  | R | AGGACGGTGGTGTAGTAGTG |
| *hsiC2* | F | CACATCTACACCGCCGAATAC |
|  | R | AGCTTTCCAGGCCGAAGAAC |
| *clpV2* | F | GCGTTTCATCACCACCATC |
|  | R | CCACCTCGTCCAGCAATATC |
| *pscN* | F | AGCATCGCCGAGTATTTCC |
|  | R | TTCCACCAGTACGGTGTAG |
| *pscF* | F | GCAGATATTCAACCCCAACC |
|  | R | GATGTTGTAGATGACCGACC |
| *popD* | F | AATATTCCCTGGCGGCTAC |
|  | R | GAATCAGCAGAAGCTCGAC |

**Table S10** Primers used for RT-PCR tests.
